# Conserved role of Ovo in germline development in mouse and *Drosophila*

**DOI:** 10.1038/srep40056

**Published:** 2017-01-06

**Authors:** Makoto Hayashi, Yuko Shinozuka, Shuji Shigenobu, Masanao Sato, Michihiko Sugimoto, Seiji Ito, Kuniya Abe, Satoru Kobayashi

**Affiliations:** 1 Life Science Center of Tsukuba Advanced Research Alliance (TARA Center), University of Tsukuba, Tsukuba, Ibaraki 305-8577, Japan; 2 Graduate School of Life and Environmental Sciences, University of Tsukuba, Tsukuba, Ibaraki 305-8572, Japan; 3 Functional Genomics Facility, NIBB Core Research Facilities, National Institute for Basic Biology, Nishigonaka 38, Myodaiji, Okazaki 444-8585, Japan; 4 Laboratory of Applied Molecular Entomology, Division of Applied Bioscience, Graduate School of Agriculture, Hokkaido University, Sapporo, 060-8589, Japan; 5 Mammalian Genome Dynamics, RIKEN BioResource Center, Tsukuba, Ibaraki 305-0074, Japan; 6 Department of Medical Chemistry, Kansai Medical University, Moriguchi, 570-8506, Japan

## Abstract

*Ovo*, which encodes a transcription factor with Zn-finger domains, is evolutionarily conserved among animals. In *Drosophila*, in addition to its zygotic function for egg production, maternal *ovo* activity is required in primordial germ cells (PGCs) for expression of germline genes such as *vasa* and *nanos*. In this study, we found that maternal Ovo accumulates in PGC nuclei during embryogenesis. In these cells, *ovo* serves a dual function: activation of genes expressed predominantly in PGCs, and conversely suppression of somatic genes. Reduction of *ovo* activity in PGCs makes them unable to develop normally into germ cells of both sexes. In mice, knockout of the *ovo* ortholog, *Ovol2*, which is expressed in PGCs, decreases the number of PGCs during early embryogenesis. These data strongly suggest that *ovo* acts as part of an evolutionarily conserved mechanism that regulates germline development in animals.

The germline is the only cell lineage destined to produce the next generation, while the soma gives rise to the body tissues. In animals, two distinct modes of germline establishment have been reported, “preformation” and “epigenesis“^1^,^2^. In the animal species with the preformation mode of germline establishment, maternal factors directing germline fate are localized in a specialized ooplasm, or germ plasm, that is partitioned into primordial germ cells (PGCs)^3^. By contrast, in certain species with the epigenesis mode, PGCs are specified by inductive signals secreted from the surrounding tissues. For example, in mice, bone morphogenetic proteins (BMPs) and Wnt signaling are both essential for PGC induction in the epiblast^4^,^5^,^6^,^7^. Irrespective of the modes of the germline establishment, zygotic expression of *vasa* (*vas*), *nanos* (*nos*), and *piwi* is evident in the PGCs of a variety of animal groups^1^,^8^. Therefore, it is possible that the mechanism underlying the activation of germline genes is conserved throughout the animal species.

To elucidate how germline gene expression is activated in PGCs, we have identified the pertinent maternal transcription factors in *Drosophila*, which uses the preformation mode of germline formation^9^. In this species, the germ plasm is localized in the posterior pole of cleavage embryos (stage 1–2) and is subsequently partitioned into PGCs, also called pole cells (stage 3–4). PGCs pass through the midgut epithelium to associate with the somatic components of the gonads (stage 9–12), and then aggregate with each other to form the embryonic gonads (stage 14). Following embryonic development, PGCs differentiate into germ cells (i.e., mature oocytes or sperm) within the gonads. Expression of *vas* begins in PGCs at around stage 9, and continues in germline cells^10^,^11^. By contrast, *piwi* and *nos* transcripts are both maternally supplied in PGCs, and their zygotic expression is evident in PGCs around stage 15^9^ (BDGP: http://www.fruitfly.org). The germline-specific expression of these genes is thought to be activated by maternal factors localized in the germ plasm. Accordingly, we identified maternal mRNAs that are enriched in the germ plasm and encode transcription factors^9^, and then performed RNA interference (RNAi)-dependent knockdown experiments in order to determine their contribution to germline-specific *vas* and *nos* expression in PGCs. The results revealed six transcripts required for *vas* and/or *nos* gene expression^9^.

*Ovo*, one of these six maternal transcripts, belongs to a family of genes encoding DNA-binding proteins with C_2_H_2_ zinc-finger domains^9^,^12^. *Ovo* is evolutionarily conserved across a wide range of animals^13^. In *Drosophila, ovo* produces three alternate isoforms: Ovo-A and Ovo-B function as a negative and positive transcriptional regulator in the germline during oogenesis, respectively^14^, whereas Svb is required for epidermal differentiation^15^,^16^. During oogenesis, Ovo-B activates transcription of *ovo* itself and *ovarian tumor* (*otu*) by binding their promoter regions, and this transcriptional activation is required for production of mature oocytes^17^,^18^. Thus, in the absence of zygotic *ovo* activity, female adults never produce progeny; consequently, genetic approaches have failed to clarify the role of maternal *ovo* in progeny. To overcome this problem, *ovo*-targeting dsRNA was injected into early embryos; however, this approach caused embryonic lethality due to the lack of epidermal differentiation, which also requires zygotic *ovo* function^9^. Thus, it remains unclear whether maternal *ovo* function is required for the normal development of the germline.

Irrespective of the two modes of germline formation, *ovo* orthologs are widely conserved among animal species^13^. For example, three *ovo* orthologs have been identified in the mouse nuclear genome^13^. The zinc-finger domains of *Ovol1, Ovol2/MOVO*, and *Ovol3* share 74%, 68%, and 58% amino-acid identity, respectively, with *Drosophila ovo*^19^,^20^. Previous work demonstrated that *Ovol2* only partially rescues the defect in *Drosophila* oogenesis caused by *ovo* mutation^20^, suggesting that the *ovo* gene family may exert similar functions in the germline in different animal species. However, the role of *ovo* orthologs in germline development in mouse embryos remains unclear.

Here we show that, in *Drosophila*, maternal Ovo-B protein is predominantly expressed in PGC nuclei. Its function is required in PGCs to activate the gene expression highly enriched in the germline, and conversely to suppress the somatic genes. Furthermore, Ovo-B must be intact for proper development of the germline both in females and males. Moreover, in mice, *ovol2* is zygotically expressed in PGCs, and its knockdown decreases the number of PGCs at early embryogenesis. Therefore, we propose that *ovo* plays an evolutionarily conserved role in the regulation of germline development in both the fruit fly and mouse.

## Results and Discussion

### Ovo-B is the major isoform in PGCs of *Drosophila* embryos

**Figure 1:**
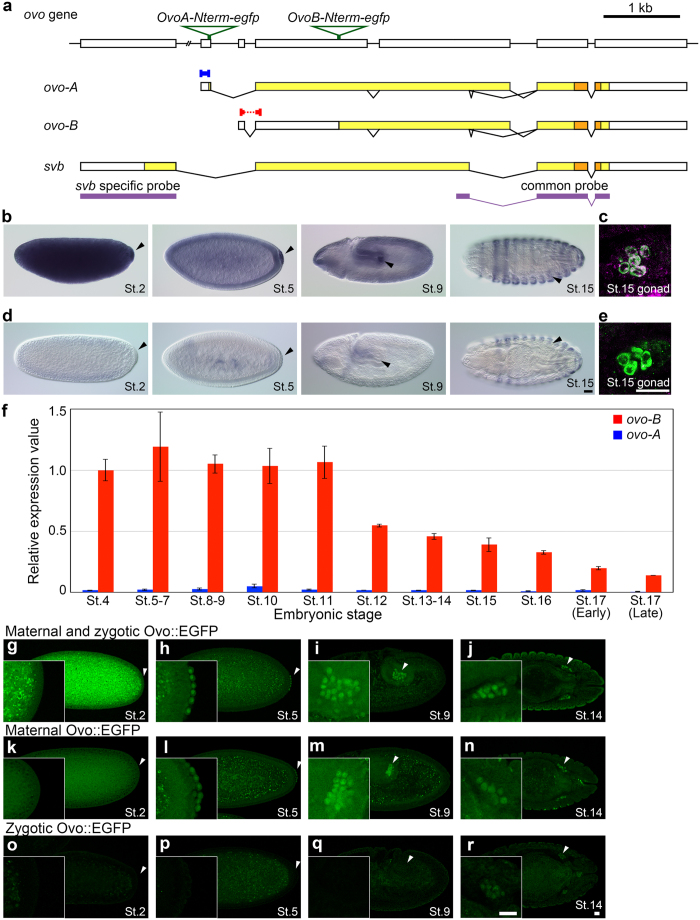
Structure of the *ovo* gene and its expression during embryogenesis. (**a**) Genomic organization of the *ovo* locus, which encodes three transcripts: *ovo-A, ovo-B*, and *svb*. Exons (boxes), introns (straight lines), protein coding regions (yellow boxes), and zinc-finger DNA-binding domains (orange boxes) are shown. Regions corresponding to the probes for *in situ* hybridization (purple bars) and the *ovo-A–* and *ovo-B*–specific region amplified by qRT-PCR analysis (blue and red bars, respectively) are also indicated. Sites where *Egfp* was inserted are indicated by green triangles. The *ovoA-Nterm-egfp* and *ovoB-Nterm-egfp* knock-in alleles contain the gene in the N-terminal regions of the Ovo-A and Ovo-B proteins, respectively. (**b**–**e**) Expression of *ovo* transcripts during embryogenesis. Probes used for *in situ* hybridization are indicated in (**a**). Embryos (**b** and **d**) and gonads (**c** and **e**) were hybridized with a common probe (**b** and **c**) and an *svb*-specific probe (**d** and **e**). Gonads (**c** and **e**) were double-stained for *ovo* or *svb* transcript (magenta) and Vasa protein (green), a marker for germ plasm and PGCs. The developmental stage of each embryo is shown in lower right. Arrowheads show germ plasm and PGCs. Scale bars: 20 μm. (**f**) The levels of *ovo-A* (blue) and *ovo-B* transcript (red) relative to the expression level of *ovo-B* in st.4 PGCs were determine by qRT-PCR, and plotted against the developmental stages of embryogenesis. Means ± SEM of three biological replicates are shown. (**g**–**r**) Expression of maternal and/or zygotic Ovo protein during embryogenesis. The Ovo-EGFP fusion protein encoded by *ovoB-Nterm-egfp* was detected using an anti-GFP antibody (green). Developmental stages are shown at lower right. Insets show close-up images of germ plasm and PGCs. Maternal and zygotic Ovo-EGFP protein was detected in embryos derived from females homozygous for *ovoB-Nterm-egfp* mated with males hemizygous for *ovoB-Nterm-egfp* (**g**–**j**). Maternal Ovo-EGFP was detected in embryos with YFP staining, derived from *ovoB-Nterm-egfp/FM7c, Dfd-GMR-nvYFP* females mated with *y w* males (**k**–**n**). Zygotic expression of Ovo-EGFP was detected in only female embryos produced from *y w* females mated with *ovoB-Nterm-egfp* hemizygous males, since the *ovo* gene is on X chromosome. Scale bars: 20 μm.

The *ovo* locus encodes three proteins, Ovo-A, Ovo-B, and Svb, which share a common C-terminal region containing Zn-finger DNA-binding domains (Fig. 1a). The signal detected by common probes for the three transcripts encoding Ovo-A, Ovo-B, and Svb is distributed almost uniformly in cleavage and syncytial blastodermal embryos from stage 2 to stage 4 (Fig. 1b). Subsequently, the signal decreases in the somatic region and is enriched in PGCs of the cellular blastodermal embryos at stage 5 (Fig. 1b). The signal remains detectable in PGCs until the end of embryogenesis (Fig. 1b,c). This spatio-temporal expression of *ovo* RNA is compatible with observations reported previously^9^,^21^,^22^. To determine which isoform is expressed in PGCs, we performed whole-mount *in situ* hybridization (WISH) and quantitative RT-PCR (qRT-PCR). WISH analysis using an *svb*-specific probe detected signal in the epidermis, but not in early embryos or PGCs (Fig. 1d,e). This observation strongly suggests that the *ovo-A* and/or *ovo-B* transcripts, but not *svb*, are present in PGCs. However, we could not confirm this by WISH using probes specific to *ovo-A* and *ovo-B*, presumably because the probes were too short (127 and 78 bases, respectively) to detect signals in embryos. qRT-PCR analysis detected both *ovo-A* and *ovo-B* transcripts in PGCs (Fig. 1f). In these experiments, we generated cDNA pools from PGCs of embryos at 11 different stages^23^,^24^, and amplified cDNAs using primer sets specific to *ovo-A* and *ovo-B* (Fig. 1a) (see Supplementary Materials and Methods). We detected *ovo-B* transcript in PGCs throughout the embryonic stages. Expression levels of *ovo-B* remained constant from stage 4 to stage 11, but decreased from stage 12 and thereafter to approximately 10% of peak levels at stage 17. By contrast, the level of *ovo-A* transcript was less than 5% of that of *ovo-B* throughout the embryonic stages. Thus, *ovo-B* is the major isoform of *ovo* transcripts in PGCs.

Next, we examined the expression of Ovo protein in PGCs. Because we were unable to raise an antibody against Ovo protein, we inserted a tag sequence encoding an enhanced green fluorescent protein (EGFP) into the N-terminus of the Ovo-B coding region (*ovoB-Nterm-egfp*) (Fig. 1a), enabling us to detect Ovo-B protein using an anti-GFP antibody. Knock-in of GFP at this site produced a GFP fusion of Ovo-A and Svb as well as Ovo-B. Given that *ovo-B* is the major isoform of *ovo* transcript in PGCs, it is reasonable to assume that the vast majority of the GFP signal detected in PGCs represents Ovo-B expression. This idea is further supported by our observation that GFP fused to the Ovo-A–specific region was undetectable in PGCs throughout embryogenesis (Supplementary Fig. S1).

In embryos produced from females homozygous for *ovoB-Nterm-egfp*, the GFP signal was ubiquitously distributed throughout embryos at stage 2 (Fig. 1g). Subsequently, the signal started to accumulate in nuclei of PGCs after stage 4, and this accumulation was maintained in the nuclei of PGCs until at least stage 16 (Fig. 1h–j). By contrast, the GFP signal was decreased in the soma, and barely detectable in their nuclei except for a subset of epidermal cells, where Svb was zygotically expressed from stage 12 onward^16^ (Fig. 1j).

The *ovo-B* transcript is supplied maternally to PGCs, and its levels decreased during embryogenesis (Fig. 1f), suggesting that zygotically expressed *ovo-B* transcript makes little, if any, contribution to the production of Ovo protein in PGCs. To confirm this, we examined zygotic expression of GFP in PGCs from the paternally transmitted *ovoB-Nterm-egfp* allele, which produced only a very weak GFP signal in female PGCs after stage 14 (Fig. 1o–r). This expression pattern is compatible with the previous finding that zygotic *ovo* expression is detectable in PGCs at stage 17 in a female specific manner^25^. By contrast, GFP expression from the maternal *ovoB-Nterm-egfp* allele was detectable in PGCs from stage 4 until at least stage 16 (Fig. 1k–n). Taken together, our observations show that Ovo-B protein produced from the maternal *ovo-B* transcript is dominant in PGCs during embryogenesis.

### Function of maternal Ovo-B is required for both female and male germline development in *Drosophila*

Next, we examined the role of maternal Ovo-B in PGCs. Because oogenesis is completely arrested in the ovaries in the absence of *ovo-B* function^14^,^26^, we were unable to obtain embryos lacking maternal *ovo-B* activity. Furthermore, knockdown of maternal *ovo-B* by injection of *ovo*-targeting dsRNA caused embryonic lethality due to the lack of epidermal differentiation^9^. Consequently, we were unable to examine the post-embryonic phenotypes caused by knockdown of maternal Ovo-B function. To overcome these problems, we used Ovo-A to specifically reduce Ovo-B activity in PGCs; this strategy was based on the prior observation that the Ovo-A isoform antagonizes Ovo-B and represses the transcription induced by Ovo-B during oogenesis^14^.

We conducted two types of Ovo-B knockdown experiments, each involving a different duration of *ovo-A* over-expression. *ovo-A* expression was induced using the *nos-Gal4-VP16* (*nos-Gal4*) transgene, which expresses the transcriptional activator Gal4-VP16 under the control of the germline-specific *nos* promoter^11^. In embryos derived from females with *nos-Gal4*, the transcript is maternally supplied and partitioned into PGCs via the *nos* 3′UTR^11^. In PGCs, Gal4-VP16 produced from the maternal transcript (maternal nos-Gal4) activated UAS-dependent gene expression from stage 11 until at least the end of embryogenesis (Supplementary Fig. S2a). By contrast, zygotic expression of nos-Gal4 (zygotic nos-Gal4) activated UAS-dependent gene expression in the germline from stage 15 to adulthood (Supplementary Fig. S2b). We induced *UASp-Ovo-A* expression in the germline by maternal and zygotic nos-Gal4 from stage 11 onward, and by maternal nos-Gal4 from stage 11 to at least the end of embryogenesis.

**Figure 2:**
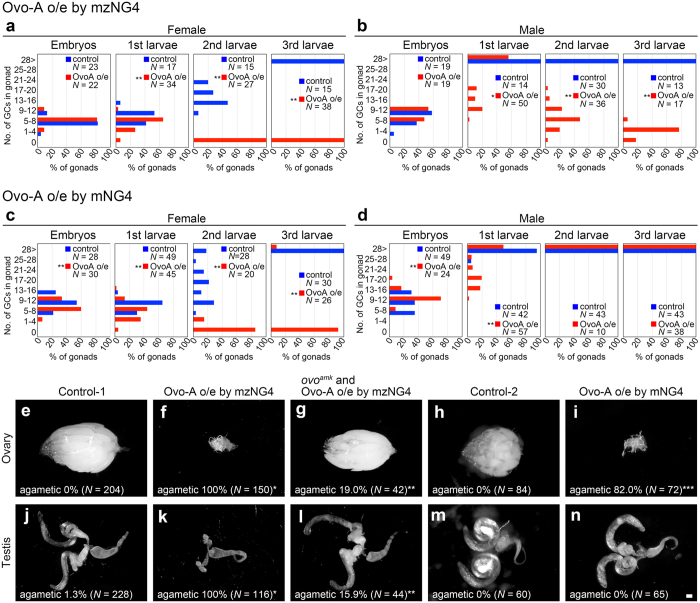
Germline-specific expression of Ovo-A causes germline loss in males and females. (**a**–**d**) Distribution of the number of Vasa-positive germline cells per gonad of Ovo-A–expressing (red) and control (blue) females (**a** and **c**) and males (**b** and **d**) at embryonic stage 15–16, the first, second, and third instar larval stages. Ovo-A was expressed under the control of mzNG4 in progeny derived from *nos-Gal4* homozygous females mated with *UASp-Ovo-A* (*line#4-2*) homozygous males (red bars in **a** and **b**). Progeny derived from *nos-Gal4* homozygous females mated with *y w* males were used as controls (blue bars in **a** and **b**). Furthermore, Ovo-A was overexpressed under the control of mNG4 in YFP-positive progeny derived from *nos-Gal4/TM6B, Dfd-GMR-nvYFP* females mated with *UASp-Ovo-A* (*line#4-2*) homozygous males (red bars in c and d). YFP-positive progeny derived from *nos-Gal4/TM6B, Dfd-GMR-nvYFP* females mated with *y w* males were used as controls (blue bars in **c** and **d**). *N*: the number of gonads observed. Significance of differences relative to controls was calculated using Fisher’s exact test (*P < 0.05, **P < 0.01). (**e**–**n**) Ovaries (**e**–**i**) and testes (**j**–**n**) of adult progeny derived from *nos-Gal4* homozygous females mated with *y w* males (control-1) (**e** and **j**), *nos-Gal4* homozygous females mated with *UASp-Ovo-A* (*line#4-2*) homozygous males (**f** and **k**), or *ovo*^*amk*^ *and nos-Gal4* homozygous females mated with *UASp-Ovo-A* (*line#4-2*) homozygous males (**g** and **l**); and YFP-positive adult progenies derived from *nos-Gal4/TM6B, Dfd-GMR-nvYFP* females mated with *y w* males (control-2) (h and m) or *nos-Gal4/TM6B, Dfd-GMR-nvYFP* females mated with *UASp-Ovo-A* (*line#4-2*) homozygous males (**i** and **n**). The *ovo*^*amk*^ transgene expresses only Ovo-B under the control of the *ovo-B* promoter^14^. Ovaries and testes were stained with an anti-Vasa antibody, and the gonads without Vasa signal were categorized as agametic. The percentage of agametic gonads is shown at the bottom of each panel. *N*: the number of gonads examined. Significance of difference relative to control-1 (*), Ovo-A o/e by mzNG4 (**), or control-2 (***) was calculated by Fischer’s exact test (*P* < 0.01). Scale bar: 100 μm.

Using this system, we first examined germline phenotypes resulting from Ovo-A expression under the control of maternal and zygotic nos-Gal4 (mzNG4). Ovo-A–expressing PGCs normally migrated into the gonads, and the number of these cells was almost identical to that observed in control embryos (Fig. 2a,b, Supplementary Fig. S3a, b). However, the number of Ovo-A–expressing germline cells during larval development was significantly reduced relative to controls (Fig. 2a,b, Supplementary Fig. S3a,b); consequently, larvae of both sexes developed into agametic adults (Fig. 2f,k, Supplementary Fig. S3e,f). Since maternal Ovo-A activity is required for germline development^14^, we cannot exclude the possibility that the above mentioned germline phenotype is caused by the upregulation of Ovo-A rather than the reduction of Ovo-B activity by Ovo-A overexpression. However, we found that Ovo-B expression from the *ovo*^*amk*^ transgene^14^ was able to rescue the agametic phenotype caused by Ovo-A expression (Fig. 2g,l, Supplementary Fig. S3e,f). This clearly indicates that this phenotype is caused by the reduction of Ovo-B activity. Therefore, Ovo-B activity is essential for germline development.

A mutation that depletes zygotic Ovo-B activity results in female-specific germline loss after the third instar larval stage^27^,^28^. This defect obviously differs from that caused by Ovo-A induction by mzNG4, which induces germline loss as early as the first instar larval stage in both sexes (Fig. 2a,b, Supplementary Fig. S3a,b). Thus, it is reasonable to speculate that maternal, rather than zygotic, Ovo-B is required in germline cells for their development in both males and females. To confirm this, we decreased maternal Ovo-B activity by inducing Ovo-A in PGCs under the control of maternal nos-Gal4 (mNG4). In females, Ovo-A expression decreased the number of germline cells during larval development (Fig. 2c, Supplementary Fig. S3c), resulting in adult sterility (Fig. 2i, Supplementary Fig. S3e). This phenotype is consistent with that caused by Ovo-A expression under the control of mzNG4 (Fig. 2a,f, Supplementary Fig. S3a,e). By contrast, in males, a subtle but statistically significant decrease in the number of germline cells was evident in first-instar larvae (Fig. 2d). However, the number of germline cells was restored to control levels after the second instar larval stage (Fig. 2d). These weak phenotypes may result from poor expression of Ovo-A in PGCs due to the half dose of maternal nos-Gal4 driver (see legend of Fig. 2). Together, these observations suggest that maternal Ovo-B is required in PGCs for normal development in females and males.

### Maternal Ovo-B activates expression of genes highly enriched in PGCs and suppresses somatic gene expression in PGCs

Because Ovo-B protein acts as a transcriptional activator^14^ and is present in nuclei of PGCs during embryogenesis (Fig. 1h–j), we speculated that maternal Ovo-B plays an important role in regulating gene expression in PGCs. To investigate this possibility, we compared transcriptomes from Ovo-B knockdown (Ovo-B KD) and control PGCs, which were isolated at stage 16 by fluorescence-activated cell sorting (FACS) from embryos expressing Ovo-A under the regulation of mzNG4 and control embryos, respectively. In our microarray analysis (see Supplementary Materials and Methods), a total of 13,167 genes were expressed in either control PGCs, Ovo-B KD PGCs, or both. Among them, 401 genes were down-regulated and 510 genes were up-regulated in Ovo-B KD PGCs (Supplementary Tables S1 and S2).

We performed Gene Ontology (GO) enrichment analysis of the genes down- or up-regulated in Ovo-B KD PGCs. Among the genes down-regulated in Ovo-B KD PGCs, no GO category was statistically significantly enriched (Supplementary Table S3). By contrast, the genes up-regulated in Ovo-B KD PGCs were enriched for GO terms associated with development of somatic tissues and organs (q-value < 0.05) (Supplementary Table S4), suggesting that Ovo-B represses expression of genes involved in somatic development in PGCs.

**Figure 3:**
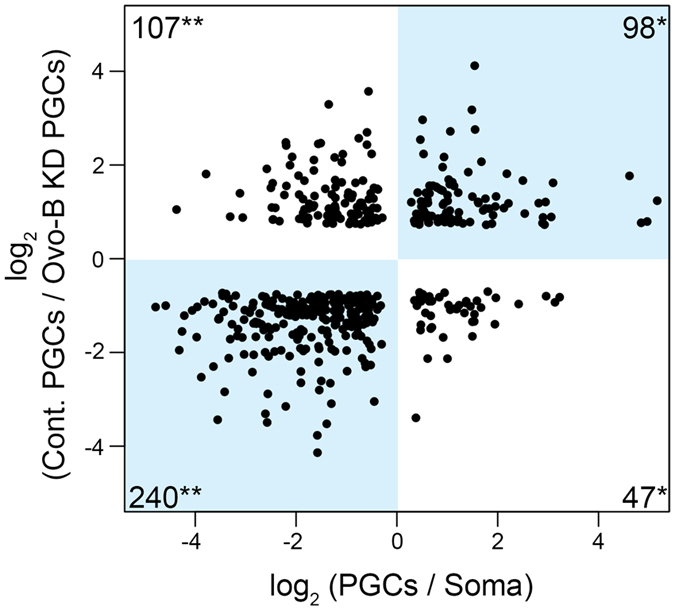
Gene expression significantly changed both in the comparison between control and Ovo-B KD PGCs, and between PGCs and whole embryos. The x-axis shows the log_2_ fold change in gene expression between PGCs and whole embryos. The y-axis shows the log_2_ fold change in gene expression between control PGCs and Ovo-B KD PGCs. Spots represent genes whose expression levels differed significantly between controls and Ovo-B KD PGCs (q-value < 0.05), and between PGCs and whole embryos (q-value < 0.05). When multiple microarray probes mapped to a single gene, the average value of the log_2_ fold change was used. The numbers of genes plotted are shown in the corners of each quadrant. Among 145 PGC-enriched genes, which are plotted in the right half, more genes were down-regulated than up-regulated by Ovo-B KD [*P < 0.01 (Fisher’s exact test)]. By contrast, among 347 soma-enriched genes, plotted in the left half, more genes were up-regulated than down-regulated by Ovo-B KD [**P < 0.01 (Fisher’s exact test)].

This is further supported by our observation that genes expressed mainly in somatic cells (soma-enriched genes) were up-regulated in Ovo-B KD PGCs, whereas genes expressed predominantly in PGCs (PGC-enriched genes) were down-regulated. We first selected 347 soma-enriched and 145 PGC-enriched genes by comparing the transcriptomes of stage-16 PGCs and whole embryos (see Supplementary Materials and Methods). Among 347 soma-enriched genes, 240 were up-regulated in Ovo-B KD (Fig. 3, Supplementary Table S5). By contrast, among 145 PGC-enriched genes, 98 were down-regulated in Ovo-B KD PGCs (Fig. 3, Supplementary Table S6).

The above data show that maternal Ovo-B plays an important role in activating expression of PGC-enriched genes, as well as in repressing somatic gene expression in PGCs. At least five genes (*CG13741, CG14838, cup, fal*, and *piwi*), whose expression patterns are annotated only with the anatomical ontology terms “germ cells and/or gonads” at stage 13–16 in the BDGP WISH database, were down-regulated in Ovo-B KD PGCs (Supplementary Table S1). According to the BDGP database, the four genes other than *fal* are specifically expressed in the germline at stage 13–16, and *cup* and *piwi* are both involved in germline development^29^,^30^, suggesting that Ovo-B is required to activate the “germline genes” in PGCs.

Three genes, *vas, nos*, and *piwi*, are regarded as the germline genes whose expression is commonly observed in the germline in multiple animal species^1^,^8^. Because probes for detecting endogenous *vas* and *nos* transcripts were unavailable in the microarrays we used (see Supplementary Materials and Methods), we monitored the expression of these genes by qRT-PCR. Our analysis revealed no significant difference in the relative expression level of *vas* between control PGCs [mean ± SEM in log_2_ (the number of biological replicates: *N*): 0 ± 0.27 (*N* = 3)] and Ovo-B KD PGCs [0.11 ± 0.14 (*N* = 3); *P* > 0.05 (Student’s *t*-test)]. Similarly, we observed no significant difference in the relative expression of *nos* between control PGCs [0 ± 0.08 (*N* = 3)] and Ovo-B KD PGCs [0.28 ± 0.22 (*N* = 3); *P* > 0.05]. However, injection of dsRNA against *ovo* into early embryos, which causes more severe defect in germline development during embryogenesis than Ovo-A overexpression does^31^, is able to suppress zygotic expression of *vas* and *nos* in PGCs^9^. Furthermore, our present microarray data shows that *piwi* expression in PGCs was also affected by Ovo-B KD (Supplementary Table S1). Based on these findings, we conclude that in PGCs, maternal Ovo-B acts as a transcriptional activator for *vas, nos*, and *piwi*, which are known to be expressed predominantly in the germline of a wide range of animal species. This led us to speculate that *ovo* plays an evolutionarily conserved role in germline development.

### Mouse *Ovol2* is required for normal development of PGCs

**Figure 4:**
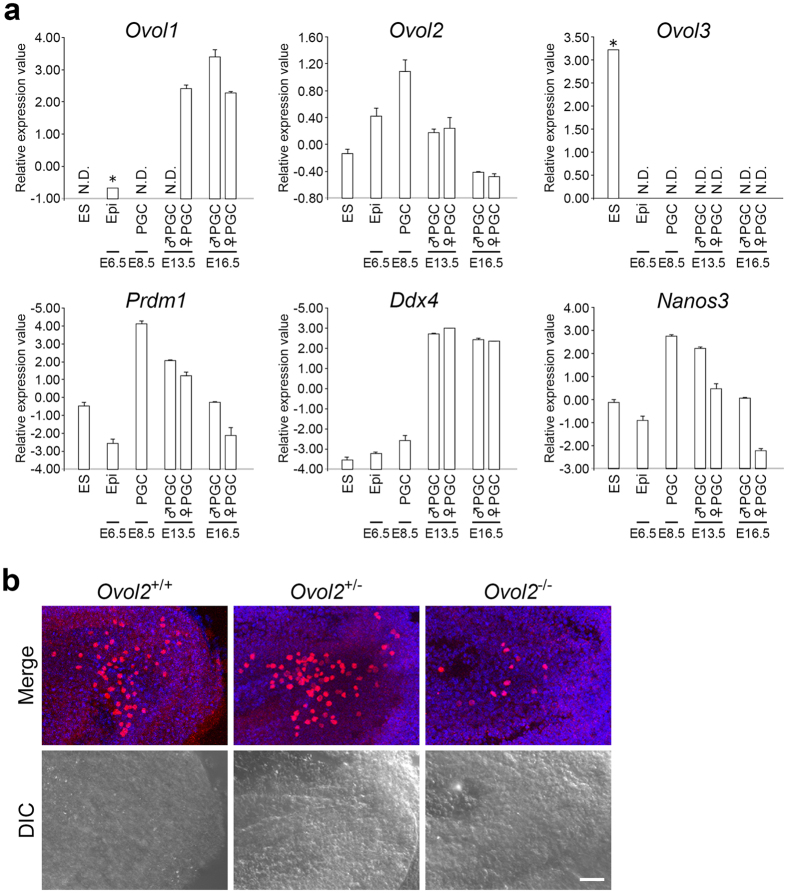
*Ovol2* is expressed in mouse PGCs and is required for their development. (**a**) Relative expression levels (log_2_ scale) of three mouse *ovo*-like genes (*Ovol1, Ovol2*, and *Ovol3*) and three germline markers (*Prdm1, Ddx4*, and *Nanos3*) were plotted against cell type (ES cells, epiblast, and PGCs) at the indicated embryonic stages. Data represent means ± SEM of two biological replicates. *Indicates that the microarray signal was detected in only one of two replicates. N.D. Indicates that no signal was detected in either replicate. Successful isolation of PGCs using FACS was confirmed by expression of the early PGC maker *Prdm1* (E8.5) and the late PGC makers *Ddx4* and *Nanos3* (E13.5 and E16.5). (**b**) PGCs stained with an anti-Oct4 antibody (red) and the nuclei stained with TO-PRO-3 (blue) in mouse E8.0 wild type (*Ovol2*^+/+^), heterozygous (*Ovol2*^+/−^) or homozygous (*Ovol2*^−/−^) embryos for *Ovol2*. Upper panels show fluorescence images, and lower panels show the corresponding differential interference contrast (DIC) images. Scale bar: 50 μm.

Mouse has three *ovo* orthologs, *Ovol1, Ovol2*, and *Ovol3*; the zinc-finger domains of these genes exhibit significant sequence similarity with *Drosophila ovo*^19^,^20^. To investigate whether an *ovo* ortholog is required for germline development in mouse embryos, we focused on *Ovol2*, which was clearly expressed in early PGCs (Fig. 4a). *Ovol2* was expressed in epiblasts of E6.5 embryos, as described previously^32^, and in PGCs isolated from E8.5 embryos; its expression level declined thereafter (Fig. 4a). Later, in spermatocytes, expression of Ovol2 protein is detected again^33^. By contrast, *Ovol3* expression was undetectable in the PGCs at E8.5, E13.5, and E16.5 (Fig. 4a). We detected *Ovol1* expression in PGCs only after E13.5 (Fig. 4a), suggesting that this gene plays a minimal role, if any, in the formation and early development of PGCs. In males, *Ovol1* is required in the germline at later stages for pachytene progression of meiotic spermatogenic cells^34^. In females, deletion of *Ovol1* gene causes reduced fertility, but this is due to somatic defects, i.e., structural abnormalities in the urogenital tract^34^.

To determine whether *Ovol2* mutation affects early germline development in mouse embryos, we counted PGCs in E8.0–E8.5 embryos, in which *Ovol2* is highly expressed (Fig. 4a). Even in the absence of *Ovol2* activity, somatic development proceeded normally, and the morphology of entire embryo and the neural and mesodermal tissues seemed to be intact at E8.0^32^,^35^. However, significantly fewer PGCs were detected by immunostaining for OCT4, a marker for PGCs, in *Ovol2* null embryos than in controls (Fig. 4b). For example, we examined embryos with three to eight pairs of somites in both control (+/−) and null mutant (−/−) embryos, and found that the null mutants contained far fewer OCT4-positive PGCs [number of PGCs ± SEM (number of embryos examined: *N*): 25.0 ± 7.5 (*N* = 6)] than controls [104.5 ± 20.5 (*N* = 4); *P* < 0.01 (Student *t*-test)]. Subsequently, the mutant embryos exhibited lethality by E10.5 due to defects in neural, gut, and heart development, and vascular angiogenesis^32^,^35^. Consequently, we were unable to follow the developmental fate of PGCs in later-stage mutant embryos.

### Evolutionarily conserved role of *ovo* in germline development in *Drosophila* and mice

The observations described above indicate that, in the absence of *Ovol2* activity, the number of PGCs was reduced in the embryos with three to eight somites (Fig. 4b). This defect resembled the phenotype observed in *Drosophila* expressing Ovo-A in PGCs (Fig. 2 and Supplementary Fig. S3). Accordingly, we propose that *ovo* plays an evolutionarily conserved role in germline development in these two animal species.

In mice, BMPs and Wnt are both required to induce PGCs in the epiblast of early embryos^4^,^5^,^6^,^7^. *Ovol2* acts downstream of the BMP signaling pathway to regulate the cell fate decision between neuroectoderm and mesendoderm^36^. In addition, the Wnt signaling pathway is required to induce *Ovol2* in the embryonic stem cells (ESC) and the colorectal cancer cells^36^,^37^. Therefore, it is reasonable to speculate that *Ovol2* is a downstream target for BMP and Wnt signaling pathways during PGC induction in epiblast. This is compatible with our data that *Ovol2* expression is upregulated in E8.5 PGCs compared with those in epiblasts and ESCs (Fig. 4a). The transcription factors (TFs), Blimp1, Prdm14, and Tfap2c, direct epiblast-like-cells (EpiLCs) into a PGC state and are also key downstream components of the BMP and Wnt pathways in the process of PGC induction^6^,^38^; therefore, *Ovol2* may act synergistically with these TFs to facilitate PGC formation.

During mouse PGC formation, these TFs activate germline and pluripotent genes, repress somatic genes, and reconstitute epigenetic programs^38^. However, the genetic network regulating PGC formation appears to be diverse even among mammals^39^. For example, reduction of *Prdm14* does not affect human PGC formation^40^, but is sufficient to induce PGC fate in mice^38^. Furthermore, *Prdm14* is not conserved in *Drosophila* genome (OrthoDB ver.9: http://www.orthodb.org), and neither expression nor function of *Blimp1* and *Tfap2c* orthologs has been reported in fly PGCs (FlyBase: http://flybase.org). These observations suggest that Blimp1, Prdm14, and Tfap2c do not act as a conserved set of TFs directing germline gene expression. By contrast, we show here that in *Drosophila*, maternal Ovo-B activates expression of genes highly enriched in PGCs and conversely suppresses somatic gene expression, thereby contributing to determination of “germness”. The *ovo* gene family is conserved across animal species, and encodes transcription factors regulating gene expression in various differentiation processes^13^. In light of the fact that an *ovo* ortholog is required for PGC formation in mice, we propose that Ovo acts as part of an evolutionarily conserved mechanism regulating the genetic network of germline development. In the future, it would be of particular interest to identify genes downstream of *Ovol2* in mouse PGCs, and to determine whether these genes also act downstream of Ovo-B in the *Drosophila* germline. Our data provide an important first step toward elucidation of a mechanism of germline gene regulation common to a wide range of animal species.

## Materials and Methods

### *Drosophila* stocks

*Nos-Gal4-VP16* (*nos-Gal4*)^11^ was used to induce expression of *UASp-Ovo-A (line#4-2, line#7, line#8)* in the germline. The *ovo*^*amk*^ transgene was used to rescue the agametic phenotype caused by Ovo-A overexpression. *ovo*^*amk*^ carries the *ovo* genomic fragment expressing only *ovo-B* mRNA under the control of *ovo-B* promoter^14^. To examine the duration of *UASp-Ovo-A* expression driven by *nos-Gal4, P{UASp-GFPS65C-aTub84B}3* (*UASp-GFP*) (Bloomington Stock center; #7373) was used. To determine the genotypes of embryos derived from females hemizygous for *nos-Gal4* and females hemizygous for *ovoB-Nterm-egfp* (see *EGFP knock-in mediated by CRISPR/Cas9*), *nos-Gal4/TM6B, P{Dfd-GMR-nvYFP}4* and *ovoB-Nterm-egfp/FM7c, P{Dfd-GMR-nvYFP}1* were used, respectively. For EGFP knock-in mediated by CRISPR/Cas9, *y*^*2*^ *cho*^2^ *v*^*1*^; *Sp/CyO, P{nos-Cas9}2 A* (NIG-Fly; CAS-0004) and *P{nos-phiC31\int.NLS}X; attP40(II)* (NIG-Fly; TBX-0002) were used. Flies were maintained on a standard *Drosophila* medium at 25 °C.

### Transgenes

The 5′- and 3′-fragments of *ovo-A* cDNA were amplified from an embryonic cDNA library^41^. Primer pairs ovoA5′-Fw/ovoA5′-Rv and ovoA3′-Fw/ovoA3′-Rv (Supplementary Table S7) were used for amplifying the 5′- and 3′-fragments, respectively. The amplified fragments were ligated using the endogenous *Not* I site and inserted into the *Kpn*I/*Hind*III sites of pBS-KS nos3′UTR^42^. Next, the *Kpn* I/*Xba* I fragment containing the *ovo-A-nos*3′UTR hybrid gene was cloned into pUASp^43^. Germline transformation was performed using *y w* embryos as recipients. Three independent *w*^+^ transformants were mated with *y w* females to establish a homozygous stock of *UASp-Ovo-A*.

### EGFP knock-in mediated by CRISPR/Cas9

See Supplementary Materials and Methods for details of the EGFP knock-in procedure.

### mRNA and protein expression analysis

See Supplementary Materials and Methods for details of the procedures used for *in situ* hybridization, quantitative RT-PCR to detect the *ovo-A* and *ovo-B* isoforms, immunostaining, microarray analysis of Ovo-B KD and control PGCs, microarray analysis of PGCs and whole embryos, and quantitative RT-PCR to detect *vas* and *nos.*

### Mouse *ovo*-like gene analysis

Microarray analysis of purified mouse PGCs was performed as described^44^. Expression levels of *ovo*-like genes and other germline markers were extracted from the microarray data. Microarray data were deposited in GEO under Accession No. GSE82020.

To count PGCs in mouse embryos, we used null-mutant mice lacking *Ovol2* exon 3, which encodes the first and second of four zinc-finger domains^35^. Oct4-positive PGCs in mouse embryos were counted as follows. Embryos of early somite stage (E8.0–8.5) were dissected and immunostained with anti-OCT4 antibody (Santa Cruz Biotechnology) as described^45^. Z-stack confocal images were taken from each embryo using a Zeiss 510, and optical slices were analyzed using the ImageJ software. OCT4-positive PGCs were detected, and their X-Y positions in each section were recorded. PGCs with the same (or almost the same) X-Y positions were considered to be identical, and total PGCs were counted by manual inspection of all optical sections.

All mouse experiments conformed to the Guide for the Care and Use of Laboratory Animals and were approved by the Institutional Committee of Laboratory Animal Experimentation of RIKEN BioResource Center.

## Additional Information

**How to cite this article**: Hayashi, M. *et al*. Conserved role of Ovo in germline development in mouse and *Drosophila. Sci. Rep.* **7**, 40056; doi: 10.1038/srep40056 (2017).

**Publisher's note:** Springer Nature remains neutral with regard to jurisdictional claims in published maps and institutional affiliations.

## Supplementary Material

Supplementary Information
