# Supplementary material for: Conserved role of Ovo in germline development in mouse and Drosophila
Source: Sci Rep. 2017 Jan 6;7:40056. doi: 10.1038/srep40056 (PMC5216385; doi:10.1038/srep40056)
Supplement: Supplementary Information [file srep40056-s1.pdf]

Supplementary Information

Conserved role of Ovo in germline development in mouse and *Drosophila*

Makoto Hayashi[#,1,2], Yuko Shinozuka[#,1], Shuji Shigenobu[3], Masanao Sato[4], Michihiko Sugimoto[†,5], Seiji Ito[6], Kuniya Abe[2,5] and Satoru Kobayashi[1,2]*

[1]Life Science Center of Tsukuba Advanced Research Alliance (TARA Center), University of Tsukuba, Tsukuba, Ibaraki 305-8577, Japan

[2]Graduate School of Life and Environmental Sciences, University of Tsukuba, Tsukuba, Ibaraki 305-8572, Japan.

[3] Functional Genomics Facility, NIBB Core Research Facilities, National Institute for Basic Biology, Nishigonaka 38, Myodaiji, Okazaki 444-8585, Japan

[4] Laboratory of Applied Molecular Entomology, Division of Applied Bioscience, Graduate School of Agriculture, Hokkaido University, Sapporo, 060-8589, Japan

[5]Mammalian Genome Dynamics, RIKEN BioResource Center, Tsukuba, Ibaraki 305-0074, Japan

[6]Department of Medical Chemistry, Kansai Medical University, Moriguchi, 570-8506, Japan

#Contributed equally to this work.

*Corresponding author: Satoru Kobayashi, E-mail: skob@tara.tsukuba.ac.jp

†Present Address: Division of Developmental Genetics, Institute of Resource Development and Analysis, Kumamoto University, 2-2-1 Honjo, Kumamoto, 860-0811, Japan

Key Words: primordial germ cells, germ line, germ plasm, *Drosophila*, mice

Short Title: Role of Ovo in germline development

Supplementary Materials and Methods

EGFP knock-in mediated by CRISPR/Cas9

The clustered regularly interspaced short palindromic repeat (CRISPR)/ CRISPR-associated endonuclease (Cas9) system was used to generate enhanced green fluorescent protein (EGFP) knock-in alleles of the *ovo* locus by expressing the guide RNA (gRNA) and Cas9 in the presence of an EGFP knock-in construct. Using CRISPR Optimal Target Finder[1], the EGFP knock-in sites were determined to be just downstream of the start codons of the Ovo-A and Ovo-B protein-coding region. The gRNA expression vectors were constructed as described[2]. The double-stranded DNAs with target sequences for *ovo-A* and *ovo-B* were constructed using pairs of complementary oligonucleotides, ovoA-egfp-sgRNA-Fw/ovoA-egfp-sgRNA-Rv and ovoB-egfp-sgRNA-Fw/ovoB-egfp-sgRNA-Rv (Supplementary Table S7), respectively, and cloned into *Bbs*I-digested pBFv-U6.2 vector[2]. These gRNA expression vectors were transformed into y^1 v^1 *nos-phiC31*; *attP40* (TBX-0002) to establish the gRNA-expressing fly lines U6.2-OvoA-Nterm and U6.2-OvoB-Nterm, as described[2].

The EGFP knock-in construct contains the EGFP-coding region flanked by the regions 1

kb upstream and downstream of the Ovo-A or Ovo-B start codon. The EGFP coding sequence was amplified from pEGFP-N1 (Clontech) using primers pEGFP-N1-Fw and pEGFP-N1-Rv (Supplementary Table S7). The genomic regions upstream and downstream of the Ovo-A start codon was amplified from genomic DNA of *y²* *cho²* *v¹* (TBX-0004) using primer pairs ovoA-up1kb-Fw/ovoA-up1kb-Rv and ovoA-dwn1kb-Fw/ovoA-dwn1kb-Rv (Supplementary Table S7). Similarly, the genomic regions upstream and downstream of Ovo-B start codon were amplified using primer pairs ovoB-up1kb-Fw/ovoB-up1kb-Rv and ovoB-dwn1kb-Fw/ovoB-dwn1kb-Rv (Supplementary Table S7). Using Gibson Assembly (New England BioLabs), the upstream, EGFP-coding, and downstream regions were assembled in that order in a pBluescript II SK(+) fragment amplified using primer pairs pBlue-Fw and pBlue-Rv (Supplementary Table S7). Approximately 0.1 nl of solution containing the *ovo-A* or *ovo-B* knock-in constructs (150 ng/μl in distilled water) was injected into the embryos derived from *y²* *cho²* *v¹; Sp/CyO, P{nos-Cas9, y+, v+}2A* (CAS-0004) females mated with U6.2-OvoA-Nterm or U6.2-OvoB-Nterm males, respectively. Progeny harboring the EGFP insertion at the *ovo-A* or *ovo-B* target site was screened by PCR, and used to establish stock lines.

In situ hybridization

Whole-mount *in situ* hybridization of embryos was performed as described[3,4] with minor modifications as follows. cDNAs corresponding to the common and *svb*-specific regions were amplified from an embryonic cDNA library[5] using primer pairs ovo-probe-Fw/ovo-probe-Rv and svb-probe-Fw/svb-probe-Rv (Supplementary Table S7), respectively. These cDNAs were inserted between the T7 and T3 promoter regions of pBluescript II SK(+). Digoxigenin (DIG)-labeled antisense RNA probes were synthesized with T3 RNA polymerase (Roche) using fragments amplified from these plasmids using the T7 and T3 primers.

Signals were detected using either an alkaline phosphatase–conjugated anti-DIG Fab fragment (Roche) or a horseradish peroxidase–conjugated anti-DIG antibody (Roche). In the latter case, signal was amplified using the TSA Biotin System and Streptavidin-Texas Red (PerkinElmer). Double staining was performed as described[4].

Quantitative RT-PCR (qRT-PCR) for *ovo-A* and *ovo-B* transcripts

qRT-PCR was performed as described [6] with minor modifications as follows. cDNA

templates for qRT-PCR were synthesized from RNA extracted from PGCs. PGCs were sorted from embryos at 11 different developmental stages [Stage (St.) 4: 1 h 20 min – 2 h 20 min after egg laying (AEL); St.5–7: 2 h 20 min – 3 h 20 min AEL; St.8–9: 3 h 20 min – 4 h 20 min AEL; St.10: 4 h 20 min – 5 h 20 min AEL; St.11: 5 h 20 min – 7 h 20 min AEL; St.12: 7 h 20 min – 9 h 20 min AEL; St.13 – 14: 9 h 20 min – 11 h 20 min AEL; St.15: 11 h 20 min – 13 h 20 min AEL; St.16: 13 h 20 min – 16 h 20min AEL; St.17 (early): 16 h 20 min – 19 h 20 min AEL; St.17 (late): 19 h 20 min – 20 h AEL][7]. Primer pairs used for amplifying *ovo-A*– and *ovo-B*–specific regions (Fig. 1a) were ovo-A-Fw/ovo-A-Rv and ovo-B-Fw/ovo-B-Rv (Supplementary Table S8), respectively. For amplification of *rp49*, primer pair rp49-Fw/p49-Rv (Supplementary Table S8) was used.

The absolute amounts of *ovo-A* and *ovo-B* transcripts were determined using external standards containing cDNAs whose nucleotide sequences are identical to those of the target sequences. This approach ensures equivalent amplification of standards and target sequences. Standard curves for each isoform were generated using a dilution series of *Xba*I-digested pBluescript II SK(+) containing an *ovo-A* or *ovo-B* cDNA fragment. cDNA fragments corresponding to *ovo-A* and *ovo-B* were amplified from an embryonic cDNA library[5] using

primer pairs ovoA-sp-Fw/ovoA-sp-Rv and ovoB-sp-Fw/ovoB-sp-Rv (Supplementary Table S7), respectively. Quantitation was performed using the ABI PRISM7000 Sequence Detection System (Applied Biosystems) and the QuantiTect SYBR Green PCR Kit (QIAGEN). Data were analyzed using Sequence Detection System Software 1.1 (Applied Biosystems) and Microsoft Excel (Microsoft). Comparing the C_T values of the *ovo-A* and *ovo-B* transcripts with standard curves allowed determination of absolute amounts. The amounts of target transcripts in PGCs from the 11 embryonic stages were normalized against the C_T values of the control gene (*rp49*). Three independent experiments using independent pools of PGCs were carried out.

Immunostaining

Immunofluorescence staining of embryos and larval gonads was carried out as described[3,4]. Larval gonads were dissected from first (36±2 h AEL), second (60±2 h AEL), and wandering third instar larvae. The following primary antibodies were used at the indicated dilutions: rabbit anti-Vasa (1:1500), rabbit anti-GFP (1:500, A11122; Life Technologies), chick anti-Vasa (1:500), mouse anti-Sxl [1:25, M18; Developmental Studies

Hybridoma Bank (DSHB)], and mouse anti-Fas3 antibody (1:50, 7G10; DSHB). For primary antibody detection, the following secondary antibodies were used: Alexa Fluor 488–conjugated goat anti-rabbit IgG (1:1000; Molecular Probe), Alexa Fluor 546–conjugated goat anti-chick IgY (1:500; Molecular Probe), and Alexa Fluor 568– or Alexa Fluor 647–conjugated goat anti-mouse IgG antibody (1:300; Molecular Probe). Stained embryos and gonads were mounted in Vectashield (Vector Laboratories) and observed under a confocal microscope (Leica Microsystems).

Microarray analysis of Ovo-B KD PGCs

Using fluorescence-activated cell sorting (FACS) as described[6], PGCs for microarray analysis were isolated from St.16 embryos (13 h 20 min – 16 h 20 min AEL) derived from females homozygous for *EGFP-vasa* and *nos-Gal4* mated with *UASp-Ovo-A* (*line#4-2*) (Ovo-B KD) or *y w* males (control). Total RNA was isolated from PGCs using the RNeasy Plus Micro kit (QIAGEN). cDNA was synthesized and amplified from 10 ng of total RNA from each sample using the Ovation PicoSL WTA System (NuGEN). The cDNAs were labeled with Cy3 using NimbleGen One-Color Labeling Kit (Roche). *Drosophila* V2 oligo

microarrays, which contain probes for 13,936 genes (GPL17080) were hybridized with 1.65 μg of Cy3-labeled cDNAs and washed using the Gene Expression Wash Buffer Kit (Agilent Technologies). The hybridized arrays were scanned using the G2505C Microarray Scanner (Agilent Technologies). Signal data were extracted using Feature Extraction ver.10.7 (Agilent Technologies). Microarray data were deposited in GEO under Accession No. GSE81857.

Raw values were normalized across samples using quantile normalization. For each probe, the following linear model was fitted to the normalized expression values:

$$E_{ij} \sim G_i + R_j + \varepsilon_{ij}$$

where E is $\log_2$ signal intensity of the normalized expression value; G and R are genotype and replicate effects, respectively; and ε is the residual. Significance was calculated for G_i. P-values were corrected for multiple tests using the Storey false discovery rate to obtain q-values[8]. The expression of endogenous *vas* and *nos* could not be examined: the microarrays we used contain no probe for the *vas* transcript, and the *nos* probe is designed to hybridize with the *nos* 3'UTR region, which allows detection of the transcript over-expressed from the *UASp-Ovo-A* transgene (see "Transgene" in Materials and Methods) as well as the endogenous *nos* transcript.

Gene ontology (GO) analysis for the genes down- and up-regulated in Ovo-B KD PGCs was performed by using DAVID 6.7 (https://david.ncifcrf.gov). Spatio-temporal expression of the genes down-regulated in Ovo-B KD PGCs were determined by using BDGP WISH database (http://www.fruitfly.org).

Microarray analysis of PGCs and whole embryos

To identify PGC-enriched genes, gene expression was compared between PGCs and whole embryos by microarray analysis. PGCs were isolated from St.16 *EGFP-vasa* embryos (13 h 20 min – 16 h 20 min AEL) by FACS as described[6]. Whole *EGFP-vasa* embryos were collected at various stages (1 h 20 min – 13 h 20 min AEL). Total RNA was extracted from isolated PGCs and whole embryos using the RNeasy Mini kit (QIAGEN). Extracted RNA was amplified as cRNA labeled with cyanine-3 CTP (Cy3) or cyanine-5 CTP (Cy5) using the Agilent Low RNA Input Fluorescent Linear Amplification Kit (Agilent Technologies). Custom-made 22K 60-mer oligo microarrays (Agilent Technologies) that contain 21,925 probes representing almost all of the predicted *Drosophila* transcripts were used (GPL4336). Each array was hybridized with 1 μg of both Cy3-labeled and Cy5-labeled cRNA and washed

according to the Agilent protocol. Dye-swap experiments were also carried out. Subsequently, arrays were scanned on a G2565BA Microarray Scanner System (Agilent Technologies). Signal data were extracted using the Feature Extraction 7.1 software (Agilent Technologies). Data obtained from four independent biological replicates were deposited in GEO under Accession No. GSE83460.

Log$_2$ ratio values from two-color microarray experiments were normalized across samples from all developmental stages, and the estimated expression values of each gene at each developmental stage were obtained using the *limma* package in R (https://bioconductor.org/packages/release/bioc/html/limma.html). Using the lmscFit function, the following model was fitted to the normalized log$_2$ expression values:

$$E_{ij} \sim C_i + D_j + \varepsilon_{ij}$$

where E is log$_2$ signal intensity of the normalized expression value; C and D are cell types [PGCs at 11 developmental stages and whole embryos (soma)] and dye effects, respectively; and ε is the residual. The sums of intercept and C_i for each probe of stage-16 PGCs and soma were used as estimated expression values. Significance was calculated for differences between the estimated values of stage-16 PGCs and soma. The P-values were corrected for

multiple tests using Holm's method.

Quantitative RT-PCR for *vas* and *nos*

Because our microarray analysis could not detect expression of endogenous *vas* and *nos* (see above), the expression levels of *vas* and *nos* were analyzed by qRT-PCR. cDNAs synthesized for microarray analysis of Ovo-B KD and control PGCs (see above) were used as templates for qRT-PCR. *vas* and *nos* were amplified using primer pairs vas-Fw/vas-Rv and nos-Fw/nos-Rv (Supplementary Table S8), respectively. *CG14967* was used as an internal control because its expression was not affected by Ovo-B KD in PGCs. *CG14967* was amplified using primer pair CG14967-Fw/CG14967-Rv (Supplementary Table S8).

Quantitation was performed using the Light Cycler 480 system (Roche) and the QuantiTect SYBR Green RT-PCR Kit (QIAGEN). Thermal cycling conditions were as follows: 50°C for 2 min and 95°C for 15 min, followed by 45 cycles of 15 sec at 94°C, 30 sec at 55°C, and 30 sec at 72°C. Data were analyzed using the Light Cycler Software (Roche) and Microsoft Excel (Microsoft). Relative expression values of target genes were normalized against the internal control (*CG14967*). Three independent experiments were carried out

using independent pools of PGCs.

Supplementary Figures

Supplementary Figure S1. Expression of maternal and zygotic Ovo-A protein during embryogenesis.

Embryos were stained for GFP (green), Vasa (red), and nuclear DNA (DAPI, blue) at the indicated stages. The Ovo-A-EGFP fusion protein (green) was not detectable in PGCs (red) of embryos derived from the females homozygous for *ovoA-Nterm-egfp* mated with males hemizygous for *ovoA-Nterm-egfp*. In lower panels, only GFP signals (green) are shown. Scale bar: 20 μm.

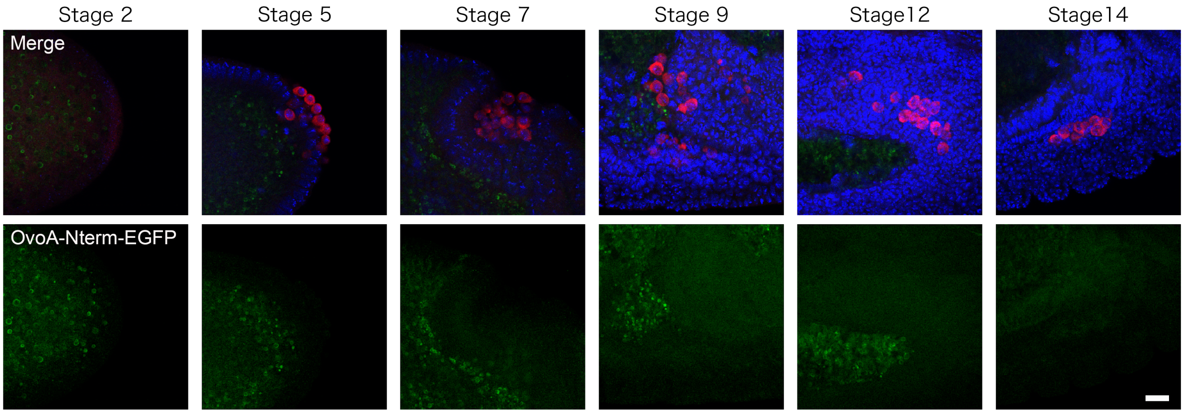

Supplementary Figure S1

Supplementary Figure S2. UAS-dependent gene expression induced by maternal or zygotic nos-Gal4.

(a and b) YFP-positive embryos derived from *nos-Gal4/TM6B, Dfd-GMR-nvYFP* females mated with males homozygous for *UASp-GFP* (a) and the embryos derived from *UASp-GFP* homozygous females mated with males homozygous for *nos-Gal4* (b) were stained for GFP (green), Vasa (red), and nuclear DNA (DAPI, blue). In lower panels, only GFP signals (green) are shown. GFP expression driven by maternal nos-Gal4 was detected in PGCs (red) from stage 11 until stage 16 (a). By contrast, GFP expression driven by zygotic nos-Gal4 was detected in PGCs (red) from stage 15 (b) to adulthood (data not shown). Scale bar: 20 μm.

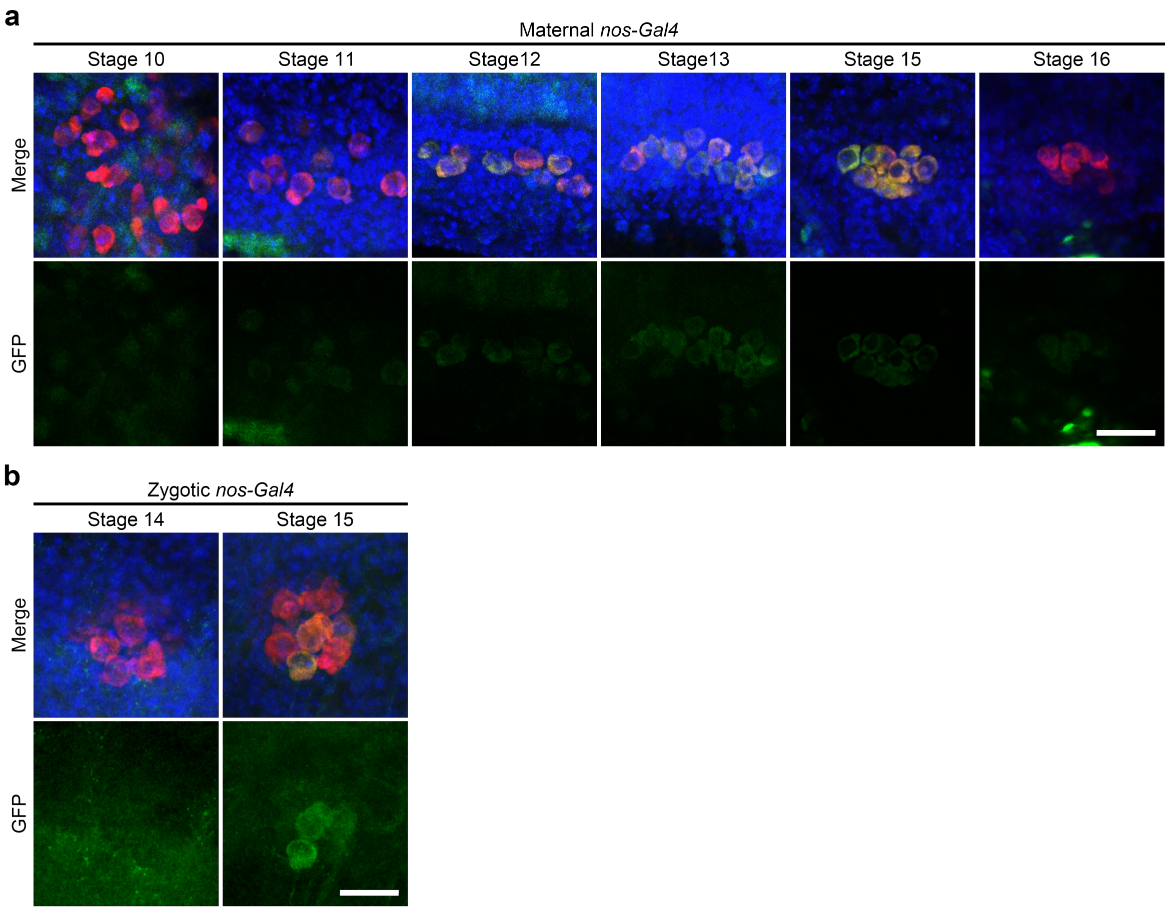

Supplementary Figure S2

Supplementary Figure S3. Ovo-A overexpression caused a loss of germline cells.

(a–d) Distribution of the number of Vasa-positive germline cells per gonad of Ovo-A–expressing (red and orange) females (a and c) and males (b and d) at embryonic stage 15–16, spanning the first through third instar larval stages. Ovo-A was expressed under the control of mzNG4 in progeny derived females homozygous for *nos-Gal4* mated with males homozygous for *UASp-Ovo-A* [line#7 (red) and line#8 (orange)] (a and b). Furthermore, Ovo-A was over-expressed under the control of mNG4 in YFP-positive progeny derived from *nos-Gal4/TM6B, Dfd-GMR-nvYFP* females mated with males homozygous for *UASp-Ovo-A* (*line#7*) (red bars in c and d). As a control, progeny derived from females homozygous for *nos-Gal4* homozygous mated with *y w* males (blue bars in Fig. 2a and b) and YFP-positive progeny derived from *nos-Gal4/TM6B, Dfd-GMR-nvYFP* females mated with *y w* males were used (blue bars in Fig. 2c and d). Significance of difference relative to controls (Fig. 2a–d) was calculated using Fisher's exact test (* $P < 0.05$, ** $P < 0.01$). *N*: the number of gonads observed. (e–f) Percentages of agametic (red) and non-agametic (blue) ovaries (e) and testes (f) of adult progeny derived from females homozygous for *nos-Gal4* mated with males homozygous for *UASp-Ovo-A* (*line#7 or line#8*) (Ovo-A o/e by mzNG4) or from females homozygous for *ovo*^{amk} *and nos-Gal4* mated with males homozygous for *UASp-Ovo-A* (*line#7 or line#8*) (*ovo*^{amk} and Ovo-A o/e by mzNG4), and from YFP-positive adult progeny derived from *nos-Gal4/TM6B, Dfd-GMR-nvYFP* females mated with males homozygous *UASp-Ovo-A* (*line#7*) (Ovo-A o/e by mNG4) are shown. The number of gonads examined is shown in parentheses. Gonads without Vasa staining were categorized as agametic. Significance of differences vs. control-1 (Fig. 2e and j) (* $P < 0.01$), “Ovo-A o/e by mzNG4” (** $P < 0.01$), or control-2 (Fig. 2h and m) (*** $P < 0.01$, $^{\#}$ $P > 0.05$) was calculated by Fischer's exact test.

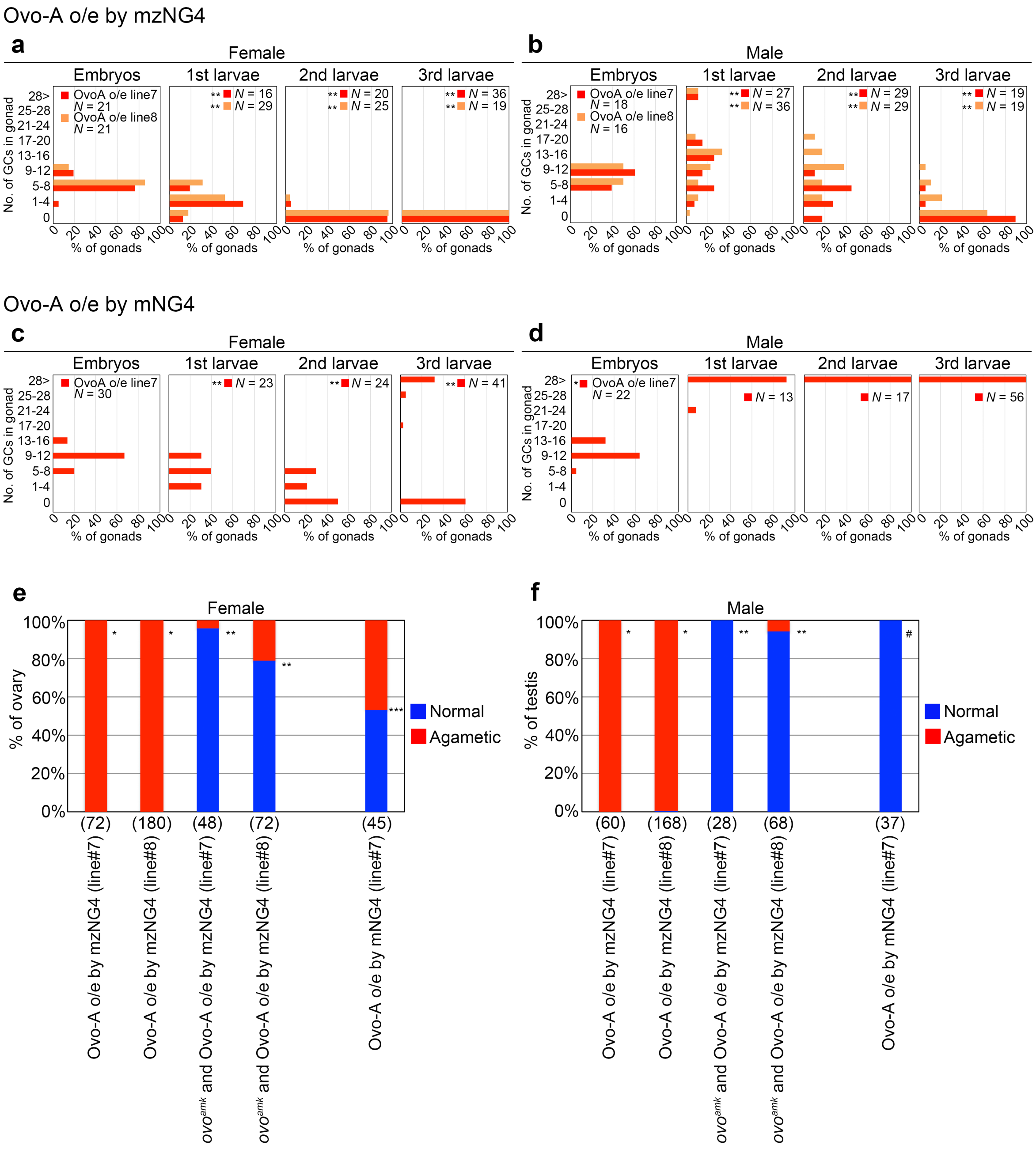

Supplementary Figure S3

Supplementary Table S1. List of genes down-regulated in Ovo-B KD PGCs

* This table shows the names of genes and probes whose expression levels were significantly down-regulated in Ovo-A–expressing PGCs in comparison with control PGCs (q-value < 0.05; see SI Materials and Methods).

† Using a linear model, the intercept and genotype effect (G_i) values were calculated for each probe (see SI Materials and Methods). The intercept value represents the estimated expression value in one of the biological replicates of Ovo-A–expressing PGCs in log_2 scale. The genotype effect value represents the estimated fold change (control PGCs / Ovo-A–expressing PGCs) in log_2 scale.

‡ The five genes, of which expression patterns are annotated only with ontology terms "germ cells and/or gonads" at stage 13-16 in BDGP WISH database.

Gene Name*	Feature Number (GPL17080)	Probe Name*	Intercept†	Genotype effect [Fold Change: log_2(Cont./Ovo-A oe)]†	p-value	q-value
Aac11	37457	A_09_P080016	10.32157	0.98231	1.654.E-03	0.03114
	40715	A_09_P080016	10.31244	1.05273	1.545.E-03	0.03029
	41427	A_09_P136550	8.35646	1.22363	4.017.E-04	0.01553
	25414	A_09_P146835	10.64688	1.05329	7.077.E-04	0.02035
aay	30941	A_09_P033236	7.23632	2.15550	1.432.E-05	0.00614
	3792	A_09_P033236	7.27678	2.30735	9.398.E-06	0.00581
Acf1	5530	A_09_P079851	9.71238	0.89283	1.453.E-03	0.02919
adp	33391	A_09_P041196	9.88703	0.81339	2.403.E-03	0.03686
	20001	A_09_P041196	10.04108	0.89528	1.940.E-03	0.03363
alpha-Est10	3628	A_09_P030956	6.50639	1.87697	3.636.E-05	0.00750
	25877	A_09_P030956	6.58602	1.93596	2.321.E-04	0.01246
	19336	A_09_P209670	7.74439	2.39547	6.884.E-05	0.00827
alpha-Est3	33886	A_09_P030966	6.43497	1.85167	6.267.E-05	0.00827
	29917	A_09_P030966	6.25633	1.92696	4.205.E-05	0.00750
	31459	A_09_P197385	8.42126	1.36907	1.976.E-04	0.01176
	33181	A_09_P197645	8.23335	1.52312	8.114.E-05	0.00873
alpha-Est4	19915	A_09_P030971	7.01767	0.84143	3.341.E-03	0.04240
alpha-Est9	10290	A_09_P030996	5.28864	1.02240	4.124.E-03	0.04693
Ank2	2723	A_09_P004301	3.01758	2.37078	1.332.E-03	0.02801
	9154	A_09_P004301	3.48722	3.07716	2.112.E-06	0.00581
	12337	A_09_P185680	4.28029	2.99650	2.371.E-04	0.01260
	31220	A_09_P207160	4.65697	1.48125	1.192.E-03	0.02657
Aprt	33149	A_09_P041311	6.75051	1.63924	1.035.E-04	0.00938
	29686	A_09_P041311	6.82298	1.69535	6.288.E-05	0.00827
ase	9137	A_09_P041351	5.12327	1.41384	5.405.E-04	0.01786
	7010	A_09_P041351	4.89700	1.61071	3.822.E-04	0.01519
asparagine-synthetase	35336	A_09_P064326	5.42830	1.32233	3.867.E-04	0.01526
	34717	A_09_P064326	5.66289	1.37769	4.485.E-04	0.01634
Asph	33022	A_09_P008181	8.36778	1.86614	2.896.E-05	0.00710
	28912	A_09_P008181	8.18493	1.95720	2.085.E-05	0.00643
	735	A_09_P008186	8.72008	2.18435	2.987.E-05	0.00710
	17356	A_09_P008186	8.68104	2.26378	6.561.E-05	0.00827
Atg8b	20994	A_09_P076271	5.92939	1.31333	1.638.E-03	0.03107
aub	18373	A_09_P041376	9.69325	1.17719	8.977.E-04	0.02299
	24248	A_09_P041376	9.65453	1.19538	1.365.E-03	0.02826
barr	5137	A_09_P030186	9.28323	0.77964	3.954.E-03	0.04588
beat-Ib	8305	A_09_P065511	1.95102	2.52072	5.015.E-05	0.00761
	906	A_09_P108505	4.85534	0.96449	2.125.E-03	0.03511
Best3	42707	A_09_P056206	4.01208	0.99697	3.650.E-03	0.04435
	41783	A_09_P113085	3.27630	1.08598	9.924.E-04	0.02419
BicC	20485	A_09_P041451	7.66174	0.76115	3.416.E-03	0.04279
	28078	A_09_P041451	7.50782	0.86565	2.743.E-03	0.03884
	38759	A_09_P166245	8.27535	0.87552	1.767.E-03	0.03209
bwa	43918	A_09_P128290	7.41807	1.47779	1.289.E-04	0.01012
cal1	13459	A_09_P076046	11.03067	0.75157	4.219.E-03	0.04735
Cam	2143	A_09_P041566	12.49561	1.34875	7.138.E-04	0.02042

	23064	A_09_P041566	12.59724	1.42995	5.398.E-04	0.01786
	39478	A_09_P044926	12.51778	0.97765	1.032.E-03	0.02456
	4351	A_09_P045311	12.60792	1.32715	1.144.E-03	0.02602
	31138	A_09_P049811	11.97452	1.17526	5.413.E-04	0.01786
	6141	A_09_P138090	7.82437	0.96985	1.027.E-03	0.02455
	7059	A_09_P203390	6.00147	1.75227	6.741.E-05	0.00827
CAP	27189	A_09_P006051	9.34594	0.94458	1.404.E-03	0.02859
	15393	A_09_P006051	9.33066	1.08496	5.486.E-04	0.01805
caps	38870	A_09_P033216	4.42489	1.27413	6.940.E-04	0.02015
	19437	A_09_P033216	4.58418	1.51769	3.556.E-04	0.01468
Cda4	32802	A_09_P015706	7.49458	0.94063	4.365.E-03	0.04825
	18799	A_09_P015706	7.55046	1.00867	2.428.E-03	0.03690
cdc16	11243	A_09_P104455	9.51242	0.84461	2.313.E-03	0.03637
CDC45L	20767	A_09_P148800	7.26631	0.73759	4.172.E-03	0.04708
Cdk4	4238	A_09_P031536	9.67385	1.26860	6.744.E-04	0.01989
	37990	A_09_P031536	9.33360	1.37437	6.246.E-04	0.01917
	1291	A_09_P121720	6.55510	1.17213	5.669.E-04	0.01842
CG10006	26681	A_09_P056061	5.01362	2.01777	1.144.E-04	0.00959
	42361	A_09_P056061	4.69425	2.59110	2.876.E-04	0.01353
	27343	A_09_P128945	6.24677	1.63540	2.585.E-04	0.01293
CG10165	21343	A_09_P023521	7.45813	1.39715	2.843.E-03	0.03952
CG10183	40671	A_09_P070711	2.48554	1.37429	4.268.E-03	0.04763
CG10200	5827	A_09_P007801	4.75034	0.85217	1.944.E-03	0.03364
CG10747	11506	A_09_P023676	8.42671	0.90017	3.455.E-03	0.04299
	16108	A_09_P023676	8.30227	0.99061	1.978.E-03	0.03392
CG10752	32402	A_09_P055546	0.70207	1.20820	9.231.E-04	0.02338
CG10863	10365	A_09_P079556	8.48285	1.55662	1.285.E-04	0.01012
	23583	A_09_P079556	8.16307	1.71623	2.152.E-04	0.01210
CG10920	19187	A_09_P067961	4.20115	0.77743	3.803.E-03	0.04484
CG11095	11846	A_09_P038546	7.20069	0.82508	4.150.E-03	0.04697
	28679	A_09_P038546	6.83729	0.88011	2.257.E-03	0.03604
CG11298	44605	A_09_P026426	4.33022	0.76351	3.468.E-03	0.04303
CG11357	23699	A_09_P052756	8.01929	0.91966	3.062.E-03	0.04079
	31359	A_09_P052756	7.93358	0.98132	1.899.E-03	0.03334
CG11368	33157	A_09_P063806	1.23176	4.26079	8.741.E-04	0.02267
	40236	A_09_P063806	1.52214	4.42414	4.080.E-05	0.00750
CG11382	43541	A_09_P062866	5.12054	2.75692	1.209.E-05	0.00598
CG11409	27879	A_09_P076921	8.77723	1.04320	8.052.E-04	0.02169
	2094	A_09_P076921	8.99133	1.09515	6.269.E-04	0.01922
CG11638	9106	A_09_P062786	8.78851	1.53144	1.784.E-04	0.01142
	20495	A_09_P062786	8.73855	1.59887	1.047.E-04	0.00943
	33878	A_09_P199575	8.15624	1.72822	1.853.E-04	0.01155
CG12001	5986	A_09_P036121	5.94409	0.76267	3.752.E-03	0.04465
CG12050	2128	A_09_P023941	9.50208	0.81181	3.004.E-03	0.04034
	14556	A_09_P023941	9.93627	0.92850	1.298.E-03	0.02759
CG12106	2728	A_09_P068481	5.10372	1.31586	3.847.E-04	0.01525
	7318	A_09_P068481	5.35448	1.35478	6.986.E-04	0.02022
CG12325	27057	A_09_P006231	8.21833	1.39413	2.741.E-04	0.01328
CG12344	30433	A_09_P006236	8.64540	1.15872	1.181.E-03	0.02646
	27637	A_09_P006236	8.60240	1.23824	6.559.E-04	0.01965
CG12355	38115	A_09_P063616	6.59449	1.54356	1.563.E-04	0.01068
	32158	A_09_P063616	6.68681	1.58538	1.058.E-04	0.00943
CG12426	18317	A_09_P161150	1.82124	3.79012	7.916.E-05	0.00859
CG12477	24914	A_09_P113955	9.47612	0.76651	3.675.E-03	0.04451
CG12499	1544	A_09_P070216	11.52412	0.75358	4.512.E-03	0.04910
	4622	A_09_P070216	11.78475	0.76818	4.077.E-03	0.04661
CG12546	583	A_09_P035811	1.42836	1.36016	1.527.E-03	0.03005
CG12637	19560	A_09_P037416	5.77028	1.37289	1.730.E-03	0.03201
CG12680	2111	A_09_P067141	7.82616	1.72783	1.950.E-04	0.01174
	19203	A_09_P067141	7.85123	1.85752	1.390.E-04	0.01036
CG12689	33280	A_09_P067926	4.50617	1.65832	2.422.E-03	0.03688
	9996	A_09_P067926	4.47451	1.79117	2.747.E-03	0.03888
CG12708	23190	A_09_P039076	2.33542	0.80594	2.481.E-03	0.03718
CG12744	38336	A_09_P005866	9.92984	1.65729	8.590.E-05	0.00882
	25426	A_09_P005866	9.81996	1.67930	6.193.E-05	0.00827

	13616	A_09_P150055	9.63559	1.73275	5.628.E-05	0.00791
CG1275	44337	A_09_P028681	5.71315	1.41303	2.081.E-03	0.03490
	25209	A_09_P028681	5.77021	1.65111	1.747.E-03	0.03204
CG12851	12656	A_09_P027781	8.20663	1.17127	3.427.E-04	0.01453
	4842	A_09_P027781	8.20895	1.20807	3.530.E-04	0.01463
CG12972	33256	A_09_P035431	7.30774	1.52066	9.672.E-04	0.02396
	66	A_09_P035431	7.23510	1.59600	9.887.E-04	0.02417
CG12991	29187	A_09_P213420	7.21005	0.77672	2.969.E-03	0.04018
CG12994	41226	A_09_P216825	7.23972	1.01152	8.678.E-04	0.02262
CG13202	34832	A_09_P006451	3.14683	0.98959	2.920.E-03	0.03996
	39724	A_09_P006451	3.27793	1.16248	3.127.E-03	0.04112
CG13350	28127	A_09_P007501	7.67760	0.85758	2.086.E-03	0.03494
	34758	A_09_P007501	7.64939	0.86338	2.701.E-03	0.03841
CG13511	36301	A_09_P026586	3.98298	0.99281	2.956.E-03	0.04013
CG13560	6946	A_09_P027071	2.78546	0.93835	3.326.E-03	0.04235
CG13594	28178	A_09_P027631	6.96266	0.80652	2.772.E-03	0.03903
CG13690	9136	A_09_P017941	9.75245	0.91053	1.776.E-03	0.03209
CG13741‡	24688	A_09_P005567	9.64566	1.18152	8.008.E-04	0.02168
CG13784	7720	A_09_P020291	2.88692	0.82950	2.580.E-03	0.03785
	41019	A_09_P020291	2.68332	1.01805	2.096.E-03	0.03496
CG13822	15227	A_09_P070721	5.59888	0.80389	4.647.E-03	0.04987
	23383	A_09_P070721	5.61122	0.87476	2.986.E-03	0.04025
CG1387	4865	A_09_P068226	6.04485	1.48945	1.775.E-03	0.03209
	12252	A_09_P068226	6.04129	1.61466	3.151.E-03	0.04125
CG14050	37880	A_09_P210345	7.82902	1.16320	4.568.E-03	0.04943
CG14059	13851	A_09_P128920	5.42325	0.71772	4.271.E-03	0.04764
CG14085	30359	A_09_P034551	6.37753	0.94123	3.046.E-03	0.04065
CG14186	278	A_09_P034866	4.37595	2.17817	3.642.E-05	0.00750
	21623	A_09_P034866	4.50048	2.36274	2.085.E-05	0.00643
CG14305	33080	A_09_P068916	5.13187	1.00760	2.322.E-03	0.03637
	24010	A_09_P068916	5.05873	1.24524	5.868.E-04	0.01866
CG14342	22558	A_09_P112510	1.38007	1.28992	5.408.E-04	0.01786
CG14416	38376	A_09_P062791	5.33423	0.88506	4.697.E-03	0.04995
	16939	A_09_P062791	5.24178	0.93274	2.144.E-03	0.03536
CG1444	35764	A_09_P068016	11.43826	1.32939	1.848.E-04	0.01154
	16966	A_09_P068016	11.68812	1.40244	1.549.E-04	0.01068
CG14512	4728	A_09_P060841	8.02411	0.89620	1.944.E-03	0.03364
	6849	A_09_P060841	7.98078	0.95377	2.021.E-03	0.03429
CG14516	7384	A_09_P060846	7.40871	2.71779	7.148.E-06	0.00581
CG14668	42775	A_09_P036316	4.87505	1.09886	1.928.E-03	0.03358
CG14694	36795	A_09_P073631	10.42576	0.86087	3.291.E-03	0.04210
	2159	A_09_P073631	10.56933	0.89052	2.514.E-03	0.03746
CG14764	44666	A_09_P024961	2.93409	3.48030	7.736.E-05	0.00851
	42721	A_09_P024961	2.69037	3.66118	4.456.E-05	0.00750
CG14805	912	A_09_P076491	9.33523	0.71733	4.311.E-03	0.04785
	11313	A_09_P076491	9.22642	0.84775	2.602.E-03	0.03798
CG14835	4468	A_09_P053571	1.70484	2.17139	1.103.E-05	0.00591
CG14837	19097	A_09_P053626	6.69840	1.16946	3.632.E-04	0.01472
	37544	A_09_P053626	6.80898	1.25208	3.102.E-04	0.01411
CG14838‡	33383	A_09_P053641	4.72046	1.34161	6.599.E-04	0.01967
	43585	A_09_P053641	4.86660	1.34592	4.846.E-04	0.01690
	4557	A_09_P117265	4.28782	2.59786	1.772.E-04	0.01142
CG14913	37482	A_09_P021886	4.02092	0.90925	2.720.E-03	0.03857
CG15034	41344	A_09_P067896	6.95372	1.50397	1.084.E-04	0.00943
	5850	A_09_P144060	7.30473	1.17709	2.574.E-03	0.03783
CG15035	33094	A_09_P067911	5.77756	1.24637	1.855.E-03	0.03297
CG15036	42245	A_09_P063801	5.99876	2.83734	4.205.E-04	0.01584
	43385	A_09_P113580	5.17574	2.52366	6.432.E-04	0.01950
CG15144	26909	A_09_P022991	4.66569	1.24310	2.114.E-03	0.03507
CG15172	7144	A_09_P023331	9.66750	1.23175	3.242.E-04	0.01440
	9622	A_09_P162685	10.29422	1.24415	4.009.E-04	0.01552
CG15312	14203	A_09_P037271	7.08468	1.27998	9.190.E-04	0.02333
	19078	A_09_P037271	7.06280	1.38899	3.221.E-04	0.01438
	21538	A_09_P195200	5.79425	0.89138	1.618.E-03	0.03094
	20020	A_09_P214965	7.15731	1.31451	5.104.E-04	0.01734

CG15390	12051	A_09_P018536	8.28966	1.51980	1.796.E-04	0.01142
	21055	A_09_P018536	8.15180	1.59605	1.192.E-04	0.00980
CG15400	32621	A_09_P018716	3.51669	1.58721	6.741.E-04	0.01989
	16566	A_09_P216915	8.27649	0.94987	4.018.E-03	0.04634
CG15506	16580	A_09_P061021	2.61563	0.80156	2.990.E-03	0.04025
CG15572	2566	A_09_P066971	2.59007	0.96962	3.910.E-03	0.04569
CG15771	44341	A_09_P067336	7.65806	1.34059	2.259.E-04	0.01239
	7416	A_09_P067336	7.91294	1.37098	2.079.E-04	0.01192
CG1640	32178	A_09_P038366	9.30690	0.80168	2.825.E-03	0.03942
	4906	A_09_P038366	9.39363	0.86754	1.772.E-03	0.03209
CG1673	27187	A_09_P038386	8.64794	0.73661	4.682.E-03	0.04995
	22794	A_09_P038386	8.69915	0.76097	3.934.E-03	0.04582
CG16833	28216	A_09_P050501	7.08749	1.19826	5.935.E-04	0.01879
	23891	A_09_P161615	3.25370	1.46113	2.069.E-04	0.01192
	39017	A_09_P197290	7.84370	1.36405	1.943.E-04	0.01173
CG16838	1901	A_09_P033406	7.94629	0.73732	4.236.E-03	0.04742
	4888	A_09_P033406	7.96409	0.79137	2.920.E-03	0.03996
CG16898	40382	A_09_P025566	2.16321	2.39160	3.706.E-03	0.04460
	26228	A_09_P025566	1.88183	2.87866	1.024.E-04	0.00938
CG17029	29010	A_09_P033316	4.94824	1.02396	1.028.E-03	0.02455
CG17075	28088	A_09_P118285	7.99250	0.95769	1.709.E-03	0.03195
CG1753	43202	A_09_P040971	6.69148	2.43304	5.858.E-06	0.00581
CG17571	44331	A_09_P126350	1.83533	0.83594	2.949.E-03	0.04013
CG17636	9435	A_09_P077966	8.13010	0.80174	3.751.E-03	0.04465
	7517	A_09_P171165	7.46929	0.82360	2.812.E-03	0.03934
CG17802	7181	A_09_P068646	6.53298	0.87903	2.533.E-03	0.03764
CG17928	39996	A_09_P022831	7.14362	1.05821	1.386.E-03	0.02851
	9762	A_09_P112685	6.96353	1.25084	8.677.E-04	0.02262
CG18155	18781	A_09_P067891	10.15933	1.07579	4.008.E-03	0.04631
	12601	A_09_P137120	8.82721	1.08436	1.641.E-03	0.03107
CG18636	44992	A_09_P022716	0.77727	1.47986	5.090.E-04	0.01733
CG1958	37468	A_09_P067866	8.37277	1.18549	1.251.E-03	0.02712
	14676	A_09_P067866	8.75643	1.23114	2.954.E-03	0.04013
CG2111	43370	A_09_P037411	3.59408	2.51368	1.144.E-03	0.02602
	14692	A_09_P037411	3.23965	2.59484	9.361.E-04	0.02356
CG30022	19229	A_09_P057369	5.93213	1.05377	4.079.E-03	0.04661
CG30089	3445	A_09_P050306	5.11611	1.55347	2.279.E-04	0.01245
CG30184	11484	A_09_P147580	5.13381	1.00448	2.913.E-03	0.03996
CG30273	4503	A_09_P057981	6.20755	0.81082	3.127.E-03	0.04112
CG3036	34049	A_09_P019351	7.95892	2.41206	4.397.E-05	0.00750
	35772	A_09_P019351	7.74602	2.48934	2.547.E-05	0.00683
CG30409	31581	A_09_P058351	7.18754	0.76087	4.039.E-03	0.04642
CG30497	7567	A_09_P179170	8.24992	1.29809	1.037.E-03	0.02464
CG31086	4224	A_09_P204735	6.30585	1.12084	1.151.E-03	0.02606
CG31098	3207	A_09_P058986	3.81235	1.27262	2.038.E-03	0.03442
CG31145	5102	A_09_P183695	3.17269	2.72927	3.993.E-05	0.00750
	44052	A_09_P225500	2.55129	1.58768	2.713.E-03	0.03850
CG31262	30835	A_09_P208875	1.72845	1.62930	7.217.E-04	0.02050
CG31313	7121	A_09_P059581	5.84213	1.33832	7.218.E-04	0.02050
CG31381	30202	A_09_P056601	6.95506	0.82645	3.486.E-03	0.04308
CG31472	20911	A_09_P059946	6.76970	2.23508	3.310.E-05	0.00750
CG31473	35831	A_09_P059951	6.30880	1.21555	3.238.E-04	0.01440
CG31807	33772	A_09_P013791	5.08008	1.54850	8.245.E-05	0.00875
	21171	A_09_P013791	5.00657	1.55489	1.105.E-04	0.00952
	34073	A_09_P217110	5.99553	0.79444	3.218.E-03	0.04170
CG31861	25457	A_09_P166490	3.07771	1.01003	1.224.E-03	0.02685
CG31975	23883	A_09_P014226	6.80592	1.26384	5.990.E-04	0.01885
	4038	A_09_P014226	6.87436	1.26772	2.497.E-04	0.01279
CG31976	1309	A_09_P014231	6.24039	0.95471	2.627.E-03	0.03809
	21026	A_09_P014231	6.26702	1.09382	5.851.E-04	0.01865
CG32061	24349	A_09_P014431	0.79010	2.42081	2.817.E-03	0.03938
CG32164	29899	A_09_P162230	10.59307	0.80191	4.607.E-03	0.04967
	25128	A_09_P191830	12.59270	0.77169	4.206.E-03	0.04732
CG32206	4864	A_09_P014911	2.98556	0.97076	2.480.E-03	0.03718
CG32318	306	A_09_P015271	10.66606	1.47523	1.939.E-04	0.01173

CG32365	7845	A_09_P015346	8.26177	1.82836	4.138.E-05	0.00750
	2938	A_09_P015346	8.23954	1.88322	4.853.E-05	0.00755
	26855	A_09_P177415	9.32109	1.96317	9.269.E-05	0.00914
CG32369	31540	A_09_P145485	2.30302	1.51168	1.255.E-04	0.01008
CG32373	44782	A_09_P015371	7.16907	1.19178	8.626.E-04	0.02258
	15630	A_09_P015371	6.68180	1.22813	7.246.E-04	0.02054
CG32412	28529	A_09_P015486	8.63849	0.81835	3.158.E-03	0.04128
	16784	A_09_P015486	8.76103	0.89166	2.188.E-03	0.03570
CG32413	24011	A_09_P015491	5.13643	0.83915	4.446.E-03	0.04873
CG3244	27692	A_09_P019281	2.39118	1.08559	7.677.E-04	0.02118
CG32442	20379	A_09_P151405	7.62737	0.81920	3.730.E-03	0.04464
CG32521	13776	A_09_P015731	3.93859	1.08703	3.484.E-03	0.04307
	40239	A_09_P202530	6.81512	0.93896	2.257.E-03	0.03604
CG32719	2626	A_09_P016331	4.33366	2.96455	2.852.E-03	0.03956
CG32726	23358	A_09_P016341	4.07058	3.60211	2.249.E-04	0.01239
CG32727	214	A_09_P016346	7.14899	1.49045	2.409.E-03	0.03686
	22733	A_09_P016346	7.10558	1.67960	1.777.E-03	0.03209
	39481	A_09_P130230	4.94156	3.32509	3.913.E-04	0.01534
CG32987	27392	A_09_P112340	1.33046	0.94975	3.423.E-03	0.04283
CG33181	44600	A_09_P017041	9.75498	1.08929	2.964.E-03	0.04013
	13104	A_09_P050611	7.01212	2.25534	1.143.E-05	0.00591
	20347	A_09_P109675	7.99406	1.74811	9.607.E-05	0.00916
	21618	A_09_P185735	6.71813	1.29700	7.064.E-04	0.02035
	37436	A_09_P192820	9.86864	1.07721	1.131.E-03	0.02581
CG33275	3014	A_09_P053651	5.67902	1.87725	1.875.E-04	0.01160
	13604	A_09_P053651	5.64482	1.88978	2.096.E-04	0.01192
CG33322	15413	A_09_P017516	3.69126	1.36970	1.791.E-03	0.03218
	38038	A_09_P214945	5.91322	0.74423	3.690.E-03	0.04458
CG33468	26122	A_09_P017646	2.47472	0.94099	3.411.E-03	0.04275
CG33477	21974	A_09_P017676	6.98758	1.30808	1.925.E-03	0.03358
CG33552	9721	A_09_P000261	7.56293	1.05835	9.034.E-04	0.02311
	6375	A_09_P000261	7.46152	1.22096	6.502.E-04	0.01965
CG33558	1667	A_09_P000311	8.76683	0.84538	2.029.E-03	0.03438
	7158	A_09_P000316	8.03402	0.95220	1.087.E-03	0.02536
	30557	A_09_P000316	7.99991	1.01349	7.366.E-04	0.02078
CG3362	41816	A_09_P027401	6.89906	1.15315	1.774.E-03	0.03209
	5434	A_09_P027401	7.20566	1.31519	4.375.E-04	0.01620
CG33665	357	A_09_P000423	4.85393	1.21511	4.365.E-04	0.01619
CG3371	29906	A_09_P028056	8.52407	1.30820	2.246.E-04	0.01239
	39863	A_09_P028056	8.39265	1.34815	2.437.E-04	0.01262
CG34040	39701	A_09_P001136	2.76334	0.72243	4.595.E-03	0.04962
CG34167	12140	A_09_P003016	2.21323	0.75922	4.188.E-03	0.04718
CG34295	33764	A_09_P003646	1.75449	0.77873	4.385.E-03	0.04843
CG34337	22255	A_09_P003816	5.52830	2.05117	6.877.E-04	0.02009
	34926	A_09_P003816	5.32189	2.16517	4.780.E-04	0.01682
	45009	A_09_P101645	2.11117	3.61760	2.931.E-04	0.01365
CG34353	38372	A_09_P003906	7.58750	3.23022	8.792.E-06	0.00581
	13762	A_09_P003906	7.76202	3.23044	2.392.E-05	0.00683
	8163	A_09_P205315	5.76986	3.77815	2.130.E-05	0.00643
	9772	A_09_P218190	7.29268	2.46910	4.792.E-04	0.01684
CG34360	12547	A_09_P063151	9.69441	1.18534	4.935.E-04	0.01698
	26978	A_09_P063151	9.39905	1.35325	5.241.E-04	0.01760
CG34386	22257	A_09_P216450	3.83800	0.89036	4.203.E-03	0.04731
CG3509	31479	A_09_P075131	5.93287	2.67381	5.775.E-06	0.00581
	28135	A_09_P075131	5.83785	2.71985	5.286.E-06	0.00581
CG3581	29392	A_09_P069171	5.61401	1.14805	1.363.E-03	0.02826
	22605	A_09_P069171	5.69494	1.16428	8.738.E-04	0.02267
CG3608	28936	A_09_P027621	8.18249	1.05520	1.404.E-03	0.02859
	23658	A_09_P027621	8.16403	1.09530	1.012.E-03	0.02447
CG3609	35091	A_09_P018531	8.56901	0.84144	2.629.E-03	0.03809
	25888	A_09_P130625	8.31456	1.38483	2.962.E-04	0.01375
CG3838	14666	A_09_P021156	6.44070	1.46864	1.443.E-04	0.01046
	43801	A_09_P021156	6.03751	1.47957	3.574.E-04	0.01468
	15304	A_09_P104790	6.37749	1.52579	4.844.E-04	0.01690
CG40006	7415	A_09_P001241	7.85098	0.91788	3.876.E-03	0.04536

	18487	A_09_P001241	7.85921	1.04792	1.903.E-03	0.03334
CG40485	22502	A_09_P002426	3.24346	1.18411	4.656.E-03	0.04989
	44065	A_09_P002426	2.79130	1.83260	9.333.E-05	0.00916
CG41562	44672	A_09_P005536	5.31538	1.04325	2.217.E-03	0.03592
CG42233	23580	A_09_P140620	10.64461	0.85627	2.354.E-03	0.03643
CG42265	22121	A_09_P067276	6.01423	2.29298	2.025.E-05	0.00643
	43215	A_09_P193465	3.92130	2.40822	1.746.E-05	0.00614
CG42340	2364	A_09_P067626	8.31704	0.83503	3.091.E-03	0.04092
	34853	A_09_P067626	8.17457	0.86675	2.113.E-03	0.03507
	39219	A_09_P067626	8.12922	0.86921	2.115.E-03	0.03507
	7761	A_09_P067626	8.14105	0.87031	1.976.E-03	0.03390
	37777	A_09_P067626	8.12014	0.87081	1.883.E-03	0.03320
	11612	A_09_P067626	8.33283	0.87445	1.569.E-03	0.03039
	34396	A_09_P067626	8.14131	0.89003	1.930.E-03	0.03358
	1333	A_09_P067626	8.25744	0.90404	1.412.E-03	0.02866
	10010	A_09_P067626	8.28128	0.91096	1.303.E-03	0.02759
	19303	A_09_P067626	8.12001	0.96995	1.265.E-03	0.02722
	25659	A_09_P179810	8.65191	0.88111	2.992.E-03	0.04025
CG42368	2609	A_09_P019951	5.04336	1.11398	9.715.E-04	0.02399
	8064	A_09_P112565	5.69624	1.07798	6.627.E-04	0.01968
CG42404	33659	A_09_P075361	6.69741	1.71340	5.708.E-05	0.00793
	2884	A_09_P075361	6.84973	1.73338	6.860.E-05	0.00827
	19986	A_09_P118075	5.02353	1.09679	1.787.E-03	0.03217
CG42492	1134	A_09_P171335	5.39659	1.55316	1.013.E-04	0.00935
	19136	A_09_P191475	6.45403	1.56964	1.897.E-03	0.03334
	13640	A_09_P192990	6.27211	2.30797	1.166.E-04	0.00968
	39723	A_09_P216680	9.26801	1.22464	1.108.E-03	0.02556
CG4250	17026	A_09_P026601	4.14393	1.24001	6.531.E-04	0.01965
	26369	A_09_P026601	3.93392	1.26823	5.861.E-04	0.01866
CG42514	10961	A_09_P014731	5.67958	1.75996	6.864.E-04	0.02009
CG42556	41147	A_09_P049031	5.35015	0.70392	4.621.E-03	0.04971
CG42669	39537	A_09_P162330	6.20550	1.42993	3.531.E-04	0.01463
CG42732	8143	A_09_P163660	7.86806	0.84861	3.036.E-03	0.04061
CG4306	34057	A_09_P034261	6.36198	1.54860	5.798.E-04	0.01853
	10242	A_09_P034261	6.69090	1.62714	4.658.E-04	0.01668
CG4364	23490	A_09_P021181	11.29735	0.80300	3.173.E-03	0.04144
CG4389	35621	A_09_P065151	9.24617	0.85633	1.795.E-03	0.03222
	1152	A_09_P065151	9.20747	0.89238	1.753.E-03	0.03205
CG4570	3549	A_09_P073626	10.90859	0.85947	2.344.E-03	0.03643
	11061	A_09_P073626	10.77019	0.92326	2.016.E-03	0.03429
CG4771	17900	A_09_P070396	9.52263	1.07370	2.222.E-03	0.03595
CG4815	37475	A_09_P111860	1.98509	1.26347	3.185.E-03	0.04150
CG5018	27695	A_09_P033426	8.92238	0.81132	2.505.E-03	0.03742
	25431	A_09_P033426	8.97812	0.81450	2.234.E-03	0.03601
CG5130	19633	A_09_P035136	9.84559	0.97004	3.214.E-03	0.04167
	34485	A_09_P035136	9.68370	1.08614	1.803.E-03	0.03231
CG5428	36797	A_09_P027036	4.04488	0.98453	1.109.E-03	0.02556
	18283	A_09_P027036	4.01809	1.14978	7.827.E-04	0.02141
CG5535	32674	A_09_P034156	10.13939	0.84850	3.131.E-03	0.04112
	27855	A_09_P034156	10.06442	0.89068	2.960.E-03	0.04013
CG5612	38185	A_09_P072386	5.03740	1.13876	1.870.E-03	0.03306
CG5718	26219	A_09_P055171	1.93003	2.29581	6.916.E-04	0.02015
CG5756	29277	A_09_P008986	3.14939	0.81339	3.382.E-03	0.04260
CG5883	6356	A_09_P055186	1.59916	1.27567	3.529.E-03	0.04342
CG5937	41737	A_09_P067491	6.34864	1.52991	9.508.E-05	0.00916
	22919	A_09_P067491	6.26863	1.70562	1.490.E-04	0.01050
CG5976	11200	A_09_P109975	6.72176	0.95993	2.056.E-03	0.03460
CG6073	9858	A_09_P124545	5.33844	1.06852	7.094.E-04	0.02035
CG6084	42697	A_09_P048596	11.17754	1.00825	1.490.E-03	0.02964
	29180	A_09_P048596	11.21196	1.05292	8.388.E-04	0.02220
CG6195	10030	A_09_P069266	9.69216	1.36477	3.777.E-04	0.01507
	18986	A_09_P191375	10.60284	1.06179	1.725.E-03	0.03199
CG6209	895	A_09_P007371	1.88809	0.95084	1.212.E-03	0.02673
CG6254	19149	A_09_P073401	6.83176	0.82702	2.831.E-03	0.03947
CG6435	16293	A_09_P008506	3.69610	0.86244	2.095.E-03	0.03496

CG6763	24089	A_09_P070606	8.29565	0.92945	1.988.E-03	0.03403
CG6770	26719	A_09_P022166	11.37559	1.02359	1.189.E-03	0.02654
	19985	A_09_P196000	11.65338	1.13696	4.688.E-04	0.01671
CG6951	22068	A_09_P034981	5.48622	1.18169	1.946.E-03	0.03365
	36070	A_09_P034981	5.47931	1.20038	3.939.E-03	0.04582
CG7252	22004	A_09_P055191	3.11545	0.97615	1.281.E-03	0.02737
CG7294	39681	A_09_P021721	5.69207	1.06536	7.098.E-04	0.02035
	8618	A_09_P021721	5.88278	1.09011	1.128.E-03	0.02576
CG7330	10567	A_09_P034226	5.51492	0.90149	4.622.E-03	0.04971
	6200	A_09_P034226	5.53510	0.93637	2.236.E-03	0.03601
CG7409	34742	A_09_P053696	3.65354	1.28698	5.771.E-04	0.01852
	8874	A_09_P053696	3.84252	1.30688	8.348.E-04	0.02216
	33847	A_09_P053696	3.58323	1.35336	1.979.E-04	0.01176
	18121	A_09_P053696	3.62163	1.36359	2.083.E-04	0.01192
	17280	A_09_P053696	3.80792	1.38314	2.864.E-04	0.01352
	32031	A_09_P053696	3.45859	1.39734	3.037.E-04	0.01394
	11593	A_09_P053696	3.81352	1.43187	1.613.E-04	0.01079
	14564	A_09_P053696	3.75682	1.49709	9.774.E-05	0.00922
	14081	A_09_P053696	3.71468	1.54639	1.476.E-04	0.01048
	32652	A_09_P053696	3.54673	1.66947	1.729.E-04	0.01129
	30019	A_09_P174425	7.32005	1.66930	1.689.E-03	0.03166
CG7422	16000	A_09_P053686	3.14524	2.22285	5.545.E-04	0.01816
CG7526	10497	A_09_P053636	2.56765	2.00981	1.548.E-03	0.03030
	15834	A_09_P053636	2.69925	2.31168	2.705.E-03	0.03842
CG7573	803	A_09_P054901	2.20115	3.59163	9.351.E-04	0.02356
CG7716	6267	A_09_P053646	3.83741	2.44645	3.005.E-05	0.00710
	34089	A_09_P053646	3.56378	2.67237	9.995.E-06	0.00581
	484	A_09_P129115	3.41527	2.56184	6.286.E-04	0.01922
CG7728	23159	A_09_P033861	8.73347	0.83831	2.899.E-03	0.03994
CG7896	40087	A_09_P061171	4.06150	0.87260	4.700.E-03	0.04995
CG7993	32768	A_09_P068766	10.74477	0.77801	3.378.E-03	0.04260
CG8147	18433	A_09_P056576	4.22239	0.89781	1.967.E-03	0.03389
CG8173	3724	A_09_P039886	9.96108	0.94165	1.078.E-03	0.02524
CG8180	9871	A_09_P007991	3.05953	1.56071	1.279.E-04	0.01012
	2653	A_09_P007991	3.16817	1.67861	3.304.E-04	0.01449
CG8306	38720	A_09_P008431	5.66413	1.50309	2.408.E-04	0.01261
	26565	A_09_P008431	5.71769	1.71991	1.901.E-04	0.01167
CG8316	14552	A_09_P039851	9.24679	0.73469	4.160.E-03	0.04702
CG8850	1581	A_09_P006766	2.54078	1.78257	9.675.E-04	0.02396
	7919	A_09_P006766	2.38230	2.34149	1.793.E-04	0.01142
CG8939	13255	A_09_P039301	10.24363	0.73142	3.830.E-03	0.04504
CG9004	43659	A_09_P028731	5.45061	1.18516	2.371.E-03	0.03656
	14338	A_09_P028731	5.86884	1.22518	1.406.E-03	0.02860
CG9013	9482	A_09_P101940	4.99921	1.14389	1.962.E-03	0.03387
CG9101	3504	A_09_P130125	2.00931	1.01843	9.398.E-04	0.02356
CG9168	10397	A_09_P201315	7.48937	0.80429	3.308.E-03	0.04224
CG9238	15228	A_09_P055951	5.81004	1.77052	1.225.E-04	0.00998
	17723	A_09_P055951	5.79950	1.84439	2.143.E-04	0.01209
CG9281	14712	A_09_P039111	11.74294	1.28406	4.963.E-04	0.01705
	31085	A_09_P148290	10.36613	0.88824	1.779.E-03	0.03209
CG9328	10530	A_09_P023841	7.76717	1.62378	7.367.E-05	0.00848
	22809	A_09_P023841	7.52252	1.81618	4.358.E-05	0.00750
	44134	A_09_P153885	7.64117	1.59749	6.401.E-05	0.00827
CG9503	40194	A_09_P038801	6.88171	1.61731	6.048.E-05	0.00822
	42117	A_09_P038801	6.86887	1.64743	7.266.E-05	0.00848
CG9650	26461	A_09_P067861	3.46074	2.62062	7.023.E-06	0.00581
	15241	A_09_P067861	3.72961	2.66550	7.324.E-06	0.00581
	33437	A_09_P188310	7.71357	1.39417	2.176.E-04	0.01220
	13897	A_09_P205000	4.70538	1.74788	3.086.E-04	0.01406
CG9657	19109	A_09_P067916	4.85831	2.90480	4.245.E-04	0.01594
	18531	A_09_P067916	4.45716	3.09079	5.122.E-04	0.01737
CG9684	35444	A_09_P072576	8.57852	0.78252	3.078.E-03	0.04087
	19599	A_09_P072576	8.73119	0.84775	1.833.E-03	0.03271
	12604	A_09_P221590	10.63833	1.17113	1.157.E-03	0.02612
CG9692	29209	A_09_P033721	7.47459	0.78943	2.692.E-03	0.03830

	15156	A_09_P033721	7.55560	0.86334	1.993.E-03	0.03404
CG9796	1858	A_09_P074751	9.29661	1.66556	5.201.E-05	0.00775
CheA7a	27262	A_09_P143670	3.54357	1.75892	1.647.E-03	0.03111
CheB42a	13705	A_09_P017581	8.19961	0.96595	1.807.E-03	0.03234
Cht8	6303	A_09_P025951	4.32495	1.08849	1.258.E-03	0.02712
Con	31884	A_09_P194775	5.24349	1.07672	4.389.E-03	0.04845
cp309	15310	A_09_P209515	3.76672	0.81936	2.338.E-03	0.03643
Cp7Fc	26772	A_09_P030346	8.61803	1.19031	3.385.E-04	0.01453
	28703	A_09_P030346	8.62639	1.23812	2.759.E-04	0.01333
Cpr47Eb	31549	A_09_P006361	0.97026	1.35503	1.873.E-03	0.03307
Cpr78Cc	1038	A_09_P035406	2.33423	1.68240	2.224.E-04	0.01233
CR43279	32858	A_09_P225025	4.53777	3.48858	1.629.E-03	0.03104
ct	22177	A_09_P090115	5.91195	2.41544	1.400.E-03	0.02857
cup‡	33200	A_09_P197885	10.61154	1.04196	1.203.E-03	0.02660
cv-c	17168	A_09_P004116	4.53898	1.36900	1.897.E-03	0.03334
CycJ	12139	A_09_P012226	8.10951	0.85435	3.359.E-03	0.04249
	38547	A_09_P012226	7.77383	0.90965	2.630.E-03	0.03809
Cyp4d8	12744	A_09_P030516	2.55666	1.78970	3.133.E-03	0.04112
Cyp4e3	28129	A_09_P052171	3.48278	1.38992	2.097.E-03	0.03496
Cyp4g1	18691	A_09_P011876	7.06546	0.86449	3.844.E-03	0.04513
	38020	A_09_P052161	9.39502	0.99451	2.704.E-03	0.03842
Cyp4g15	32301	A_09_P037731	2.44186	1.21507	2.462.E-03	0.03700
Cyp6a20	41546	A_09_P007841	4.81855	1.38399	2.726.E-04	0.01328
	3199	A_09_P007841	5.04666	1.43675	1.899.E-04	0.01167
	25399	A_09_P132155	4.87815	1.78291	1.034.E-04	0.00938
Cyp6v1	41227	A_09_P204725	7.16094	0.86825	2.591.E-03	0.03797
dl	2019	A_09_P041991	7.12534	1.54431	1.196.E-03	0.02657
	45180	A_09_P041991	6.71387	1.65219	8.478.E-04	0.02234
	2378	A_09_P119495	7.18162	1.44138	9.427.E-04	0.02361
	9978	A_09_P181510	8.17108	1.00686	8.685.E-04	0.02262
Dms	24938	A_09_P013221	3.04683	1.06986	6.591.E-04	0.01967
DNApol-alpha50	39528	A_09_P029361	7.08566	1.06948	1.558.E-03	0.03039
DNApol-alpha73	9531	A_09_P011801	8.12992	0.76826	4.396.E-03	0.04849
dpa	27708	A_09_P031391	10.90661	0.86320	2.821.E-03	0.03939
	22479	A_09_P031391	10.99776	0.95863	1.900.E-03	0.03334
dpp	37919	A_09_P042056	3.45354	1.04909	4.361.E-03	0.04825
dpr6	1635	A_09_P190075	8.10727	0.98308	2.605.E-03	0.03799
dpr8	19993	A_09_P049351	8.08439	1.57986	8.567.E-05	0.00882
egr	32455	A_09_P005981	9.18526	0.80665	3.411.E-03	0.04275
	10347	A_09_P135340	4.66703	1.17491	4.452.E-04	0.01633
eIF4E-5	44038	A_09_P053701	6.23137	0.91984	2.970.E-03	0.04018
Eip55E	40113	A_09_P042201	9.54103	1.12655	1.214.E-03	0.02673
	8619	A_09_P042201	9.70458	1.13020	9.472.E-04	0.02364
esg	40208	A_09_P043141	7.13682	0.94591	2.186.E-03	0.03570
etaTry	6551	A_09_P111005	1.36159	1.34593	1.006.E-03	0.02439
Ets98B	32859	A_09_P011736	7.03804	0.96383	4.284.E-03	0.04770
	34262	A_09_P011736	7.03644	1.00820	2.292.E-03	0.03628
	15204	A_09_P174640	3.31708	3.12430	1.433.E-06	0.00581
	32772	A_09_P226175	5.33719	1.52041	2.350.E-04	0.01256
fal‡	14063	A_09_P064906	11.79443	0.78284	4.664.E-03	0.04990
fas	43043	A_09_P225680	6.50711	1.12128	4.608.E-03	0.04967
Fas2	30038	A_09_P042336	7.30270	1.54539	1.232.E-04	0.00999
	22293	A_09_P042336	7.18386	1.71477	4.869.E-05	0.00755
	23101	A_09_P042341	11.29321	1.77562	3.837.E-05	0.00750
	24759	A_09_P042341	11.33567	1.89741	2.979.E-05	0.00710
	25786	A_09_P117980	8.16105	1.59422	5.609.E-04	0.01828
fog	39330	A_09_P042406	6.66846	1.60927	7.042.E-05	0.00842
	15008	A_09_P042406	7.22117	1.62407	5.686.E-05	0.00793
	40297	A_09_P106105	7.06437	1.37451	3.316.E-04	0.01452
	36137	A_09_P177795	5.17419	1.51219	1.424.E-04	0.01044
fs(1)M3	3686	A_09_P011436	1.63511	4.07929	3.779.E-06	0.00581
	26649	A_09_P011436	1.21733	4.15980	4.039.E-06	0.00581
Fsh	28808	A_09_P031551	3.05591	1.90866	3.063.E-05	0.00710
	24107	A_09_P031551	2.97902	2.00131	4.206.E-05	0.00750
gammaTub37C	39685	A_09_P011941	5.82724	1.84772	1.137.E-04	0.00957

glob2	18069	A_09_P036856	3.22373	0.86353	2.337.E-03	0.03643
gnu	27797	A_09_P042566	6.53680	1.61822	6.675.E-04	0.01980
	45158	A_09_P042566	6.40120	1.63158	7.472.E-04	0.02096
Gs1	34273	A_09_P042626	8.90526	2.46133	1.965.E-05	0.00643
	37936	A_09_P042626	8.92905	2.47230	1.515.E-05	0.00614
GstE1	12640	A_09_P009096	7.22229	0.78934	4.011.E-03	0.04631
	459	A_09_P009096	7.20189	0.81632	2.291.E-03	0.03628
	8615	A_09_P167250	8.28091	2.62894	1.035.E-04	0.00938
Gycalpha99B	23342	A_09_P029986	3.94207	1.10458	4.301.E-03	0.04778
	20483	A_09_P029986	4.04801	1.12247	4.607.E-04	0.01664
hkb	11882	A_09_P042726	2.99449	0.87866	3.736.E-03	0.04464
Hml	33671	A_09_P066536	1.74447	0.77091	4.027.E-03	0.04640
Hn	3333	A_09_P042741	6.92389	1.52988	1.111.E-03	0.02556
	20491	A_09_P042741	6.78143	1.70367	7.842.E-04	0.02141
	34875	A_09_P046041	5.63440	1.74542	3.909.E-05	0.00750
	38996	A_09_P177255	5.84544	2.76116	6.619.E-05	0.00827
hoip	30094	A_09_P030816	11.28970	0.78310	4.544.E-03	0.04929
Ih	13434	A_09_P065071	6.88427	1.61059	1.476.E-03	0.02949
	17774	A_09_P065071	6.86457	1.66408	1.615.E-03	0.03094
Ir48b	42720	A_09_P006526	4.87566	0.96386	3.448.E-03	0.04295
	37246	A_09_P006526	4.72733	1.12767	2.237.E-03	0.03601
Jheh3	17376	A_09_P009331	6.24551	1.16701	4.402.E-04	0.01626
	18187	A_09_P009331	6.53641	1.18173	4.513.E-04	0.01640
kkv	38044	A_09_P042961	8.34837	1.72738	6.544.E-05	0.00827
	6851	A_09_P042961	8.56210	1.90095	7.461.E-05	0.00848
l(1)G0193	34116	A_09_P079171	9.85179	0.74431	3.939.E-03	0.04582
l(1)G0196	38469	A_09_P079166	9.84940	0.92093	1.203.E-03	0.02660
	29336	A_09_P079166	9.89377	0.96705	9.670.E-04	0.02396
	34522	A_09_P180275	5.66442	0.95616	1.662.E-03	0.03126
	39621	A_09_P225480	6.96327	0.99037	1.551.E-03	0.03032
l(2)35Df	16061	A_09_P043151	8.64008	0.81269	2.614.E-03	0.03802
	24851	A_09_P043151	8.52923	0.91632	3.599.E-03	0.04391
l(2)k09022	13567	A_09_P048786	10.44788	0.90829	1.738.E-03	0.03202
	44687	A_09_P048786	9.95441	0.94029	3.109.E-03	0.04102
l(2)tid	21765	A_09_P043231	8.91836	0.75394	4.073.E-03	0.04661
La	29968	A_09_P013281	12.30058	0.72385	4.303.E-03	0.04778
lap	12006	A_09_P176935	4.97584	0.90914	1.286.E-03	0.02744
Lasp	35348	A_09_P001951	10.40798	0.87398	2.265.E-03	0.03604
	15322	A_09_P001951	10.68985	0.95239	1.444.E-03	0.02913
	4661	A_09_P137855	10.28689	0.90724	2.949.E-03	0.04013
lectin-33A	43505	A_09_P062351	5.60918	1.14221	1.205.E-03	0.02661
	7649	A_09_P062351	5.90727	1.23443	7.433.E-04	0.02087
ltd	14988	A_09_P043386	9.37065	1.29614	3.430.E-04	0.01453
Mat89Ba	22679	A_09_P075736	9.55659	0.79692	3.519.E-03	0.04335
Mcm5	20839	A_09_P031881	11.58439	0.74247	4.075.E-03	0.04661
Mcm7	4488	A_09_P032536	10.84710	1.01016	1.771.E-03	0.03209
	7913	A_09_P032536	10.65361	1.03920	9.205.E-04	0.02333
	24051	A_09_P169240	7.92897	0.83428	2.446.E-03	0.03690
metl	1968	A_09_P196905	11.13459	0.95860	1.396.E-03	0.02854
mol	6084	A_09_P178065	5.59518	1.17310	2.598.E-03	0.03797
mspo	6774	A_09_P032146	4.19156	1.70335	3.248.E-04	0.01440
mtg	22590	A_09_P072466	8.31339	1.24046	3.867.E-04	0.01526
	7546	A_09_P072466	8.50341	1.32153	2.064.E-04	0.01192
mthl10	32707	A_09_P219000	5.20174	0.81198	3.055.E-03	0.04073
Muc68Ca	6380	A_09_P194615	11.43714	0.98803	1.856.E-03	0.03297
Mys45A	3322	A_09_P005591	10.21926	0.92881	2.446.E-03	0.03690
	9360	A_09_P005591	10.41506	0.93939	3.443.E-03	0.04295
NAAT1	12492	A_09_P067216	3.35057	0.85404	3.456.E-03	0.04299
NaCP60E	36318	A_09_P204800	8.44478	0.78214	3.094.E-03	0.04092
nct	15878	A_09_P107320	0.76766	3.29393	1.596.E-05	0.00614
neuroligin	24507	A_09_P105940	5.70217	1.11530	1.127.E-03	0.02576
Nha1	7436	A_09_P020161	6.95470	1.06593	9.451.E-04	0.02364
Nhe3	42615	A_09_P065681	6.72605	1.08441	2.268.E-03	0.03604
	29367	A_09_P065681	6.78293	1.16611	8.391.E-04	0.02220
	28789	A_09_P065686	10.33816	0.95471	2.320.E-03	0.03637

	34865	A_09_P065686	10.34799	0.95575	2.261.E-03	0.03604
	43568	A_09_P187125	9.79477	0.78064	3.774.E-03	0.04475
nht	28189	A_09_P064001	7.79938	1.39538	1.014.E-03	0.02447
	22204	A_09_P064001	7.72404	1.44709	1.512.E-04	0.01054
	6091	A_09_P111250	6.51046	1.38605	3.350.E-03	0.04245
nrv3	11261	A_09_P183115	9.38808	1.64389	1.086.E-04	0.00943
	14276	A_09_P194020	6.60249	1.14232	4.335.E-04	0.01610
	40071	A_09_P194995	8.44283	1.57381	7.462.E-05	0.00848
Nup358	27248	A_09_P071521	11.51392	0.77794	2.861.E-03	0.03962
nvd	34459	A_09_P001271	3.54500	1.17848	1.403.E-03	0.02859
nwk	27310	A_09_P054251	4.45079	2.48664	3.541.E-04	0.01465
	23671	A_09_P054251	4.30086	2.59582	4.108.E-04	0.01569
olf413	40340	A_09_P035726	7.13559	2.56315	3.784.E-05	0.00750
	12059	A_09_P035726	7.51483	2.57715	5.378.E-05	0.00782
Optix	24280	A_09_P077356	2.12962	1.39630	1.339.E-04	0.01017
Or98a	8565	A_09_P072436	1.58943	3.01281	4.664.E-05	0.00755
	11069	A_09_P072436	1.33908	3.49626	9.487.E-05	0.00916
orb	37166	A_09_P011246	10.36217	0.98445	1.094.E-03	0.02544
	16291	A_09_P011251	8.83002	1.77385	1.572.E-04	0.01068
	20239	A_09_P050141	8.41344	1.25188	2.601.E-04	0.01293
	34906	A_09_P182220	9.47575	1.61142	3.289.E-04	0.01448
Ork1	8913	A_09_P031856	6.20329	1.00081	2.324.E-03	0.03637
	40854	A_09_P031856	6.36905	1.01113	1.616.E-03	0.03094
Osi2	11390	A_09_P036666	1.84275	1.06999	2.515.E-03	0.03746
otp	30604	A_09_P030881	5.49524	0.74859	3.583.E-03	0.04379
	35703	A_09_P030881	5.50473	0.76397	3.446.E-03	0.04295
out	6378	A_09_P065116	3.24808	1.61652	2.435.E-03	0.03690
	23205	A_09_P065116	3.09205	2.33954	4.572.E-05	0.00755
	72	A_09_P181240	6.15710	1.36344	1.110.E-03	0.02556
ovo	24176	A_09_P134245	9.45726	2.99455	1.379.E-05	0.00614
pcl	25859	A_09_P029436	7.23735	1.23475	3.525.E-03	0.04339
	12309	A_09_P151080	5.42550	1.53364	1.346.E-03	0.02822
Peritrophin-A	3218	A_09_P033066	7.14464	1.05166	7.543.E-04	0.02104
	13265	A_09_P033066	7.32251	1.28756	5.549.E-04	0.01816
pes	44208	A_09_P020551	7.91447	0.85623	3.178.E-03	0.04146
	21169	A_09_P020551	8.04017	0.90002	3.349.E-03	0.04245
pex13	41711	A_09_P007186	8.88773	0.72987	4.654.E-03	0.04989
pgant2	32375	A_09_P018961	6.63724	0.95443	1.304.E-03	0.02760
	16146	A_09_P018961	6.45537	0.99859	1.190.E-03	0.02654
	25281	A_09_P046461	4.81428	1.59846	2.090.E-04	0.01192
pgant8	17843	A_09_P176305	4.95447	0.88803	2.304.E-03	0.03631
PGRP-SD	42898	A_09_P053661	5.12134	2.06881	9.199.E-04	0.02333
pip	27136	A_09_P044091	6.70287	0.89481	2.424.E-03	0.03688
	37959	A_09_P044106	3.19722	0.74930	3.829.E-03	0.04504
	12488	A_09_P044106	2.10416	1.77028	7.868.E-04	0.02146
piwi‡	12346	A_09_P011196	9.81845	0.72688	4.250.E-03	0.04753
Pole2	41533	A_09_P053081	7.57541	1.05258	4.544.E-03	0.04929
ppk25	9326	A_09_P017586	6.78252	1.19539	1.300.E-03	0.02759
	21855	A_09_P017586	6.53069	1.25011	1.654.E-03	0.03114
Ptp61F	28528	A_09_P186330	8.88904	1.05424	3.358.E-03	0.04249
	28726	A_09_P208270	9.24918	1.14027	2.680.E-03	0.03830
ptr	17411	A_09_P044251	8.32461	1.04079	1.148.E-03	0.02604
Pxt	38691	A_09_P076266	9.72739	2.02480	6.388.E-05	0.00827
	40153	A_09_P076266	9.65551	2.05329	7.172.E-05	0.00848
qin	42962	A_09_P068931	9.26062	0.82135	2.949.E-03	0.04013
	13327	A_09_P068931	9.35063	0.82767	2.294.E-03	0.03628
	42009	A_09_P163755	10.52872	1.14506	6.228.E-04	0.01916
	27447	A_09_P206515	11.28578	1.05972	9.466.E-04	0.02364
r-l	21963	A_09_P044421	11.18524	0.81127	2.346.E-03	0.03643
Rad51D	14015	A_09_P040131	4.26700	0.74996	3.444.E-03	0.04295
Rcd4	9227	A_09_P020831	10.67925	0.82988	2.561.E-03	0.03778
	7097	A_09_P020831	10.42750	0.95393	2.008.E-03	0.03424
RecQ4	3236	A_09_P049651	9.51218	0.80247	2.419.E-03	0.03686
retn	6895	A_09_P184730	6.92206	2.17384	1.828.E-05	0.00620
rhi	42902	A_09_P010496	7.30921	0.86864	2.838.E-03	0.03948

	33161	A_09_P010496	7.48053	0.92478	1.486.E-03	0.02961
Rhp	13984	A_09_P203000	8.38214	0.83368	3.647.E-03	0.04434
RnrS	9148	A_09_P013416	10.75776	1.16878	3.682.E-04	0.01479
	26541	A_09_P013416	10.40492	1.21757	4.030.E-04	0.01553
RpI135	24949	A_09_P044476	7.08459	1.05528	1.669.E-03	0.03133
RpL22-like	32995	A_09_P026846	12.48789	0.76439	3.762.E-03	0.04466
	2427	A_09_P026846	12.57733	0.82294	2.346.E-03	0.03643
RpS23	27238	A_09_P147930	7.71298	1.03666	2.718.E-03	0.03855
Rpt4R	31156	A_09_P055181	9.34162	1.72181	4.912.E-05	0.00755
	536	A_09_P055181	9.14790	1.81370	6.758.E-05	0.00827
rt	21510	A_09_P044491	7.48664	0.88116	4.682.E-03	0.04995
Samuel	42495	A_09_P110020	2.57092	2.11664	4.900.E-05	0.00755
Sip1	2122	A_09_P012691	7.04629	2.17087	1.680.E-05	0.00614
SIP2	21826	A_09_P001836	2.57423	1.02739	1.053.E-03	0.02491
SIP3	30979	A_09_P001831	4.68607	1.44373	2.317.E-04	0.01246
	42026	A_09_P001831	4.63091	1.60140	1.589.E-04	0.01068
SK	5891	A_09_P067206	5.88828	1.11847	1.650.E-03	0.03112
	20577	A_09_P067206	5.74826	1.65960	2.427.E-04	0.01261
	10575	A_09_P140795	3.39093	1.19753	4.728.E-04	0.01674
smp-30	18243	A_09_P075141	2.94408	1.00536	1.668.E-03	0.03133
Sod	33844	A_09_P206585	11.70559	1.22420	1.736.E-03	0.03202
Sodh-2	22800	A_09_P032981	8.71579	1.15443	6.388.E-04	0.01941
Spargel	42961	A_09_P036066	9.29096	0.78814	2.638.E-03	0.03819
	15141	A_09_P036066	9.45071	0.92574	1.302.E-03	0.02759
spd-2	23052	A_09_P079366	8.65395	1.10390	9.637.E-04	0.02394
squ	33919	A_09_P043511	8.36509	2.10795	2.065.E-04	0.01192
	24365	A_09_P043511	8.22563	2.18953	1.344.E-04	0.01017
	11366	A_09_P183335	10.84434	1.60551	5.265.E-04	0.01760
Sr-CII	41149	A_09_P032281	6.21994	1.85155	3.055.E-05	0.00710
	29331	A_09_P032281	6.18564	1.98368	2.128.E-05	0.00643
Src64B	18547	A_09_P177640	9.59919	0.74301	4.520.E-03	0.04913
stnA	18399	A_09_P031726	3.33443	1.12302	1.158.E-03	0.02612
	3693	A_09_P031726	2.97305	1.18137	4.935.E-04	0.01698
Tace	9895	A_09_P061201	6.63546	1.39977	1.546.E-04	0.01068
	8764	A_09_P061201	6.87018	1.43754	2.406.E-04	0.01261
	35353	A_09_P146000	6.33923	1.54903	3.392.E-04	0.01453
tej	43307	A_09_P007621	10.53115	1.17814	3.655.E-04	0.01478
	20904	A_09_P007621	10.67833	1.19986	3.243.E-04	0.01440
Thd1	39076	A_09_P078941	6.39539	0.97511	4.485.E-03	0.04890
	38071	A_09_P078941	6.39104	1.03998	2.160.E-03	0.03546
tio	1169	A_09_P117925	2.92021	1.92329	2.551.E-05	0.00683
Tm1	8716	A_09_P009761	9.60208	0.92074	2.613.E-03	0.03802
	29875	A_09_P009766	10.50495	0.73479	3.684.E-03	0.04457
	19811	A_09_P051276	7.68926	0.93771	2.242.E-03	0.03604
	8909	A_09_P222575	9.86525	0.79671	2.991.E-03	0.04025
TM4SF	13973	A_09_P032271	6.85766	0.74671	4.146.E-03	0.04697
	40692	A_09_P193295	8.96559	0.74827	3.751.E-03	0.04465
Tpi	30955	A_09_P048636	4.87234	2.83220	5.649.E-06	0.00581
	31132	A_09_P048636	4.78340	2.89566	2.536.E-06	0.00581
	35695	A_09_P146560	4.42384	0.98157	2.151.E-03	0.03542
Tusp	1795	A_09_P072391	7.15992	1.45967	1.133.E-04	0.00957
	16451	A_09_P103905	7.60642	1.38417	3.006.E-04	0.01382
TyrRII	41943	A_09_P113315	2.85937	0.86322	2.958.E-03	0.04013
UK114	27747	A_09_P065256	5.66720	0.75818	4.504.E-03	0.04905
Vha100-2	32000	A_09_P065571	7.66057	1.09828	2.037.E-03	0.03442
Vha14	43477	A_09_P012491	10.15813	0.90016	3.072.E-03	0.04082
	12154	A_09_P012491	10.50298	0.95263	1.366.E-03	0.02826
Vsx2	17887	A_09_P000916	3.39454	1.02729	4.655.E-03	0.04989
wcd	17401	A_09_P032871	8.29566	0.85746	3.458.E-03	0.04299
	3601	A_09_P032871	8.14105	0.89827	2.953.E-03	0.04013
WRNexo	42255	A_09_P068851	7.99140	0.89637	3.429.E-03	0.04283
	5551	A_09_P068851	7.88870	1.12812	4.627.E-04	0.01664
	10226	A_09_P121180	6.95113	1.25403	2.704.E-04	0.01327
Wsck	43234	A_09_P057126	5.65839	1.78685	8.925.E-05	0.00894
	6602	A_09_P057126	6.10104	1.93007	5.806.E-05	0.00798

	15966	A_09_P109735	7.50715	1.09692	5.673.E-04	0.01842
wun	29278	A_09_P107200	8.90602	0.83181	4.134.E-03	0.04695
X11Lbeta	20683	A_09_P016181	8.50393	0.74111	3.747.E-03	0.04465
	5628	A_09_P110490	9.70257	1.19622	1.071.E-03	0.02516
	27079	A_09_P162075	9.66480	2.28391	8.358.E-05	0.00880
	20516	A_09_P175655	8.10764	2.52931	1.481.E-04	0.01048
	31465	A_09_P183230	6.24915	1.41219	2.327.E-04	0.01246
yip3	15888	A_09_P062256	7.91497	0.89117	4.421.E-03	0.04858
yip7	23457	A_09_P062251	4.06294	1.38799	7.092.E-04	0.02035
	40401	A_09_P062251	4.22243	1.41459	1.589.E-04	0.01068
yl	44405	A_09_P010936	7.27731	1.87818	7.693.E-05	0.00851
	21111	A_09_P010936	7.38170	2.08576	8.432.E-05	0.00880
	19682	A_09_P106275	5.29924	2.02206	4.531.E-05	0.00755

Supplementary Table S2. List of genes up-regulated in Ovo-B KD PGCs

* This table shows the names of genes and probes whose expression levels were significantly up-regulated in Ovo-A–expressing PGCs in comparison with control PGCs (q-value < 0.05; see SI Materials and Methods). *nos* probe, which detected the *nos* 3'UTR region, is excluded from this table (see SI Materials and Methods). In addition, one of the two *ovo* probes detecting the *ovo-A* transcript over-expressed from *UASp-Ovo-A* in PGCs was excluded.

† Using a linear model, the intercept and genotype effect (G_i) values were calculated for each probe (see SI Materials and Methods). The intercept value represents the estimated expression value in one of the biological replicates of Ovo-A–expressing PGCs in $\log_2$ scale. The genotype effect value represents the estimated fold change (control PGCs / Ovo-A–expressing PGCs) in $\log_2$ scale.

Gene Name*	Feature Number (GPL17080)	Probe Name*	Intercept†	Genotype effect [Fold Change: $\log_2$(Cont./Ovo-A oe)]†	p-value	q-value
5-HT1A	43665	A_09_P010201	4.88620	-1.29298	7.603E-04	0.02114
	23027	A_09_P010201	5.04582	-1.07664	6.282E-04	0.01922
abd-A	12788	A_09_P041071	8.82588	-4.65331	3.530E-05	0.00750
	22071	A_09_P041071	8.84113	-4.30235	3.853E-05	0.00750
	5347	A_09_P052391	9.32651	-1.52833	9.107E-05	0.00901
Abd-B	40218	A_09_P052351	7.73206	-2.65341	3.583E-04	0.01468
	20805	A_09_P052346	7.72384	-2.59784	2.087E-05	0.00643
	26764	A_09_P041076	10.15074	-1.91713	3.932E-04	0.01537
	29190	A_09_P041076	10.24545	-1.80021	3.596E-04	0.01468
	35341	A_09_P051891	7.94651	-1.42704	9.798E-04	0.02405
Ac78C	21915	A_09_P076641	6.16453	-2.16062	4.156E-05	0.00750
	33688	A_09_P076641	6.15068	-2.00685	1.751E-04	0.01137
AcCoAS	13391	A_09_P029491	8.28247	-0.83384	2.902E-03	0.03994
Acp53C14b	16294	A_09_P008461	6.33078	-1.07001	2.406E-03	0.03686
Acp76A	32605	A_09_P031021	5.47733	-1.82085	9.175E-04	0.02333
	8601	A_09_P031021	5.34418	-1.74131	9.602E-05	0.00916
	22687	A_09_P113135	5.18700	-1.32670	4.559E-04	0.01654
Actbeta	19531	A_09_P077111	8.12811	-1.46589	9.382E-04	0.02356
	28381	A_09_P077111	8.06478	-1.46208	5.756E-04	0.01852
ade5	8843	A_09_P032451	12.41676	-0.82462	2.430E-03	0.03690
	435	A_09_P032451	12.09536	-0.81867	4.222E-03	0.04735
Adk3	29007	A_09_P064471	12.09433	-1.43622	1.173E-04	0.00968
	23085	A_09_P064471	12.00691	-1.38244	2.145E-04	0.01209
Aldh	20565	A_09_P029496	8.25741	-1.09435	7.831E-04	0.02141
	29958	A_09_P029496	8.23857	-1.03488	8.952E-04	0.02299
Aldh-III	16256	A_09_P181080	11.15100	-0.82655	3.991E-03	0.04619
alphaPS4	9239	A_09_P213310	7.90447	-0.98961	2.653E-03	0.03825
	17265	A_09_P007946	6.07868	-0.85363	3.929E-03	0.04582
Andorra	27781	A_09_P040031	4.33422	-2.12321	1.939E-03	0.03363
AnnIX	38045	A_09_P166180	10.36516	-0.93589	3.005E-03	0.04034
aop	2136	A_09_P041291	10.35195	-1.24297	7.983E-04	0.02168
	32150	A_09_P041291	10.55751	-1.23171	1.029E-03	0.02455
Arc1	16628	A_09_P007636	8.60343	-0.98051	2.054E-03	0.03460
	14034	A_09_P007636	8.58192	-0.86109	4.493E-03	0.04895
Arc2	717	A_09_P007646	6.71058	-1.75783	4.409E-05	0.00750
	12683	A_09_P007646	6.77227	-1.71420	4.358E-05	0.00750
ASPP	988	A_09_P026031	9.39928	-1.48103	5.765E-04	0.01852
	23764	A_09_P026031	9.30898	-1.38254	4.477E-04	0.01634
Ast-C	19513	A_09_P151090	4.02196	-0.81309	2.443E-03	0.03690
bbg	13649	A_09_P048181	9.75868	-0.77625	3.375E-03	0.04260
Best1	42359	A_09_P107175	8.02604	-1.01017	4.626E-03	0.04972
	37472	A_09_P062481	11.13896	-0.81767	4.575E-03	0.04949
Best2	12996	A_09_P053256	6.05646	-2.17707	4.156E-05	0.00750
	34214	A_09_P053256	5.86832	-1.86535	8.041E-04	0.02169
betaTub60D	38436	A_09_P009901	7.20770	-1.36343	3.328E-04	0.01453
	25740	A_09_P009901	7.25615	-1.22272	1.254E-03	0.02712
BG642312	10941	A_09_P057276	4.92469	-3.15451	6.161E-04	0.01910

	3992	A_09_P057276	4.74260	-2.58092	1.725E-03	0.03199
bgm	14579	A_09_P079256	8.59979	-0.87859	2.992E-03	0.04025
	4531	A_09_P079256	8.44638	-0.86023	3.299E-03	0.04216
	5272	A_09_P110773	10.10705	-0.82139	3.379E-03	0.04260
blot	3575	A_09_P079886	7.54998	-2.44042	2.343E-03	0.03643
	24204	A_09_P079886	7.71122	-2.39978	1.041E-03	0.02469
bnb	3533	A_09_P042516	12.93538	-1.05711	7.063E-04	0.02035
	26570	A_09_P042516	12.72112	-0.99733	1.171E-03	0.02634
bowl	11503	A_09_P011276	6.07733	-1.46985	8.292E-04	0.02206
	33128	A_09_P011276	5.82521	-1.22790	3.132E-03	0.04112
Brd	7429	A_09_P041496	3.47105	-1.57909	6.381E-04	0.01941
	45209	A_09_P041496	3.37207	-1.52591	4.206E-04	0.01584
brp	30218	A_09_P215895	6.47167	-0.91484	3.925E-03	0.04582
bru-2	21208	A_09_P056661	6.39715	-1.34279	3.481E-03	0.04307
	4274	A_09_P056661	6.39904	-1.24075	3.136E-03	0.04114
	20031	A_09_P226000	8.70126	-0.84767	2.781E-03	0.03909
Buffy	3362	A_09_P063031	8.23384	-1.14835	5.882E-04	0.01866
Calx	38079	A_09_P051221	6.00843	-1.20751	3.181E-03	0.04148
capa	37147	A_09_P104995	5.82228	-1.43551	1.431E-03	0.02900
CBP	10636	A_09_P106320	11.72879	-1.38570	1.612E-04	0.01079
Cct2	6054	A_09_P028321	6.84936	-0.83478	4.063E-03	0.04660
Cda9	28905	A_09_P008611	4.87275	-1.23047	2.823E-04	0.01338
CG10082	17141	A_09_P109995	11.32154	-1.20117	1.711E-03	0.03195
	16771	A_09_P026136	11.33588	-1.09765	1.808E-03	0.03235
CG10176	2508	A_09_P023131	4.20303	-1.00795	3.697E-03	0.04459
CG10337	18581	A_09_P023536	11.41697	-0.94877	2.084E-03	0.03493
CG10630	32420	A_09_P218915	15.32646	-0.82160	3.355E-03	0.04249
CG10764	23975	A_09_P008686	5.75397	-1.36667	2.692E-03	0.03830
	31850	A_09_P008686	5.79399	-1.31212	2.034E-03	0.03442
CG11033	37635	A_09_P072866	10.12426	-1.29093	2.660E-04	0.01313
	36960	A_09_P072866	10.16106	-1.27785	2.520E-04	0.01286
CG1124	19971	A_09_P036191	5.40921	-1.45253	6.241E-04	0.01917
CG11275	16210	A_09_P026356	9.16507	-1.71959	1.316E-04	0.01017
	26498	A_09_P026356	9.25067	-1.58540	1.540E-04	0.01067
CG11289	558	A_09_P020261	6.89787	-1.75447	1.466E-04	0.01048
	9892	A_09_P020261	6.96303	-1.68795	1.115E-04	0.00954
CG11347	34645	A_09_P052686	7.81329	-3.38167	3.358E-06	0.00581
	43192	A_09_P052686	7.49549	-3.23783	7.974E-06	0.00581
CG11400	12926	A_09_P008616	5.36361	-0.75674	3.429E-03	0.04283
CG11669	19406	A_09_P025166	7.10233	-1.60662	3.032E-03	0.04057
	34765	A_09_P025166	6.96728	-1.57796	8.005E-04	0.02168
CG11825	43977	A_09_P107930	9.33970	-1.48870	2.301E-04	0.01246
	25124	A_09_P006106	9.95881	-1.38373	1.807E-04	0.01142
	9572	A_09_P176590	9.57296	-1.18176	2.549E-03	0.03772
CG11883	27645	A_09_P006151	9.63018	-0.79628	2.762E-03	0.03895
CG11997	12716	A_09_P072881	7.55794	-0.99075	1.372E-03	0.02826
	34345	A_09_P072881	7.48228	-0.98172	3.381E-03	0.04260
	10118	A_09_P072881	7.40103	-0.93975	2.682E-03	0.03830
	39899	A_09_P072881	7.39247	-0.93290	3.692E-03	0.04458
	8328	A_09_P072881	7.49420	-0.92961	3.267E-03	0.04201
	38880	A_09_P072881	7.39461	-0.91766	4.038E-03	0.04642
	9511	A_09_P072881	7.52736	-0.89966	3.566E-03	0.04369
CG12035	8485	A_09_P028481	6.17414	-0.82724	2.686E-03	0.03830
CG12071	18751	A_09_P061516	9.58070	-0.72020	4.454E-03	0.04877
CG12171	36148	A_09_P036451	7.31222	-1.27095	2.153E-03	0.03542
	35902	A_09_P036451	7.38530	-1.24402	1.811E-03	0.03235
CG12214	23385	A_09_P006016	11.33162	-0.95740	4.698E-03	0.04995
CG12262	39508	A_09_P053671	11.36774	-1.01017	9.716E-04	0.02399
	21243	A_09_P053671	11.55169	-0.94926	1.345E-03	0.02822
CG12420	42252	A_09_P073411	3.56460	-1.63101	2.532E-04	0.01286
	4402	A_09_P073411	3.84340	-1.11513	1.721E-03	0.03196
	27977	A_09_P217695	5.34113	-0.85730	2.663E-03	0.03829
CG12512	4762	A_09_P019566	7.29005	-1.57165	2.429E-04	0.01261
	24972	A_09_P019566	7.13150	-1.02811	4.563E-03	0.04941
CG12535	30770	A_09_P066821	9.61003	-1.16193	1.254E-03	0.02712

	29934	A_09_P066821	9.66773	-1.12752	2.152E-03	0.03542
	2002	A_09_P101155	9.90208	-0.82996	4.455E-03	0.04877
CG12581	6222	A_09_P035926	8.65017	-1.50271	3.258E-03	0.04201
CG12730	12559	A_09_P067256	6.71834	-1.53057	1.802E-04	0.01142
	31911	A_09_P067256	6.60653	-1.51118	6.624E-04	0.01968
	10927	A_09_P067256	6.84929	-1.50347	9.479E-04	0.02364
	26014	A_09_P067256	6.66112	-1.49858	1.098E-03	0.02548
	21173	A_09_P067256	6.58117	-1.48500	3.927E-04	0.01537
	9464	A_09_P067256	6.81766	-1.45104	6.832E-04	0.02006
	41888	A_09_P067256	6.40474	-1.34042	8.561E-04	0.02248
	25527	A_09_P067256	6.52118	-1.33013	6.613E-04	0.01968
	34019	A_09_P067256	6.54865	-1.30426	1.018E-03	0.02450
	5280	A_09_P067256	6.52353	-1.22799	9.757E-04	0.02401
CG12768	37853	A_09_P035906	7.42334	-2.68350	7.207E-05	0.00848
	31874	A_09_P035906	7.07584	-2.26927	1.288E-04	0.01012
	17076	A_09_P127480	6.28394	-2.24731	1.303E-04	0.01014
CG12814	36279	A_09_P073406	5.81459	-1.85248	3.122E-03	0.04110
	20668	A_09_P217690	9.08785	-0.88986	1.865E-03	0.03304
CG12896	13280	A_09_P006111	6.98697	-1.38091	5.171E-04	0.01748
CG12917	1139	A_09_P006001	8.58976	-0.88944	4.416E-03	0.04857
CG13004	11377	A_09_P039601	6.12177	-1.91026	6.988E-04	0.02022
	27472	A_09_P193025	5.59401	-1.59879	2.127E-03	0.03511
CG13028	30651	A_09_P033806	7.49499	-0.76042	3.295E-03	0.04212
CG13085	32304	A_09_P023441	7.92719	-1.22269	1.348E-03	0.02822
CG13160	28256	A_09_P213470	4.81284	-1.69546	4.348E-03	0.04815
	36716	A_09_P190165	4.57525	-0.79817	3.944E-03	0.04583
CG13177	5089	A_09_P063516	10.75268	-0.89251	2.857E-03	0.03958
CG13239	4715	A_09_P111480	5.51164	-1.39317	1.567E-04	0.01068
CG13252	35449	A_09_P035206	4.53344	-2.54063	4.214E-03	0.04733
	27084	A_09_P035206	4.29299	-1.86880	4.089E-04	0.01566
CG13253	10301	A_09_P217515	4.21507	-1.39896	8.450E-04	0.02231
CG13323	29901	A_09_P216360	7.30796	-0.81321	2.414E-03	0.03686
CG13360	12977	A_09_P077556	7.20492	-1.02622	2.232E-03	0.03600
CG13405	6131	A_09_P027831	11.20524	-0.96226	3.639E-03	0.04429
	32895	A_09_P027831	11.15423	-0.96177	3.506E-03	0.04324
CG13481	9487	A_09_P217425	7.26665	-0.83636	3.586E-03	0.04381
CG13506	25461	A_09_P026436	6.14908	-0.73265	4.020E-03	0.04634
CG13579	14293	A_09_P027496	6.90784	-1.92530	5.697E-04	0.01842
	5767	A_09_P027496	7.03266	-1.62333	1.186E-03	0.02653
CG13830	10070	A_09_P070546	6.53033	-1.33188	2.134E-04	0.01208
	30719	A_09_P208300	7.28016	-0.93387	2.020E-03	0.03429
CG13868	10349	A_09_P025661	9.36663	-3.21434	2.287E-05	0.00666
	4276	A_09_P025661	9.08431	-2.97264	1.177E-05	0.00594
CG13928	34987	A_09_P028391	10.33402	-1.38823	1.105E-03	0.02556
CG14034	20298	A_09_P019511	3.37468	-1.84677	1.437E-04	0.01046
CG14253	32595	A_09_P185065	9.91043	-0.81271	2.870E-03	0.03966
CG14277	32806	A_09_P020711	7.15704	-1.05752	2.548E-03	0.03772
CG14302	6122	A_09_P068971	6.38724	-0.87226	2.576E-03	0.03784
CG14315	2328	A_09_P111570	6.78272	-1.26156	7.634E-04	0.02115
	39303	A_09_P068701	7.14255	-0.83815	2.618E-03	0.03806
CG14439	40675	A_09_P130240	7.16030	-1.20323	1.810E-03	0.03235
CG14492	16325	A_09_P008916	6.16031	-0.97538	2.019E-03	0.03429
	7471	A_09_P008916	6.07434	-0.71984	4.586E-03	0.04957
CG14527	40583	A_09_P060730	8.17382	-1.01557	4.148E-03	0.04697
CG14535	44533	A_09_P020516	11.00899	-2.21957	4.001E-04	0.01552
	4263	A_09_P020516	11.07164	-2.12554	3.502E-04	0.01461
CG14589	40883	A_09_P024391	4.56428	-1.70202	3.191E-03	0.04151
	12999	A_09_P024391	4.94810	-1.60769	1.464E-03	0.02928
CG14628	40661	A_09_P062856	9.85539	-0.93015	3.972E-03	0.04605
	27641	A_09_P062856	9.88449	-0.86527	3.227E-03	0.04178
CG14661	35287	A_09_P036181	3.80736	-2.35413	2.341E-03	0.03643
CG14708	37492	A_09_P073861	9.12790	-1.24566	5.968E-04	0.01885
	40241	A_09_P148460	8.32330	-1.22822	4.398E-04	0.01626
CG14718	11745	A_09_P073986	6.40672	-0.86663	4.012E-03	0.04631
CG14741	12883	A_09_P074166	7.15423	-0.91010	2.467E-03	0.03704

CG14840	1721	A_09_P075006	4.01688	-0.94920	1.911E-03	0.03340
CG14915	14177	A_09_P217065	5.78055	-1.32382	8.244E-04	0.02200
	23204	A_09_P021896	3.63001	-1.22148	9.916E-04	0.02419
	14166	A_09_P021896	3.74808	-1.09734	6.036E-04	0.01897
CG1492	14833	A_09_P037971	8.63129	-1.02127	2.867E-03	0.03966
	29013	A_09_P037971	8.49738	-0.95766	2.393E-03	0.03678
CG14937	28453	A_09_P022071	9.24047	-1.37077	5.003E-04	0.01710
	21725	A_09_P022071	9.19263	-1.22106	7.178E-04	0.02048
CG14961	19351	A_09_P029096	10.02987	-1.14175	7.940E-04	0.02161
CG14968	15551	A_09_P029061	8.60816	-0.80225	4.479E-03	0.04890
CG15056	31948	A_09_P040081	9.68487	-0.96720	3.706E-03	0.04460
CG15098	15610	A_09_P009296	11.94329	-0.83388	2.450E-03	0.03690
	13965	A_09_P009296	11.87818	-0.78679	3.817E-03	0.04497
CG15247	5017	A_09_P068596	2.85645	-1.01613	3.912E-03	0.04569
CG15357	25166	A_09_P063426	7.59969	-0.95585	2.665E-03	0.03829
	42611	A_09_P063426	7.61405	-0.94079	2.962E-03	0.04013
CG15414	32249	A_09_P111710	6.99707	-1.62020	1.451E-04	0.01046
CG15479	4850	A_09_P022531	6.08417	-2.49881	1.147E-05	0.00591
	19585	A_09_P022531	6.17359	-2.46842	2.984E-05	0.00710
	9107	A_09_P022531	6.14726	-2.46643	9.885E-06	0.00581
	43269	A_09_P022531	5.93971	-2.44884	3.195E-04	0.01437
	45086	A_09_P022531	5.98073	-2.41092	8.678E-06	0.00581
	20858	A_09_P022531	6.11332	-2.36610	1.083E-04	0.00943
	12087	A_09_P022531	5.91393	-2.22335	1.226E-04	0.00998
	36112	A_09_P022531	5.97827	-2.11446	4.013E-05	0.00750
	16017	A_09_P022531	5.93577	-2.01648	1.930E-04	0.01170
	33318	A_09_P022531	5.94570	-1.99696	8.610E-05	0.00882
CG15482	36816	A_09_P022481	5.77694	-0.96544	4.036E-03	0.04642
CG15533	41121	A_09_P061361	5.37895	-0.75658	3.701E-03	0.04460
CG15643	31545	A_09_P039021	4.97022	-0.71847	4.458E-03	0.04879
CG15658	10894	A_09_P026011	6.45706	-1.10718	1.516E-03	0.02997
	41168	A_09_P026011	6.36525	-1.05859	2.208E-03	0.03590
CG1571	8202	A_09_P068076	10.48036	-0.79924	2.572E-03	0.03783
CG1572	1540	A_09_P037751	8.95114	-1.20352	2.497E-03	0.03735
CG1600	7214	A_09_P024801	8.92088	-1.09944	1.291E-03	0.02752
CG16791	17496	A_09_P175930	10.06165	-0.89426	1.618E-03	0.03094
CG16820	14725	A_09_P022541	3.77880	-1.64269	8.811E-05	0.00890
CG16996	10917	A_09_P022216	7.90635	-1.32646	1.789E-04	0.01142
	33398	A_09_P022216	7.58347	-1.13763	4.133E-04	0.01571
CG16997	6486	A_09_P022221	7.55631	-0.78259	4.462E-03	0.04880
CG17150	32906	A_09_P047421	6.74510	-1.23895	4.592E-04	0.01661
CG17154	32722	A_09_P055261	7.49625	-1.11070	1.350E-03	0.02822
CG17181	12550	A_09_P214545	6.39960	-1.05986	9.761E-04	0.02401
	31575	A_09_P028011	8.27407	-1.05239	4.137E-03	0.04695
CG17211	11391	A_09_P022226	7.54212	-2.03247	3.810E-03	0.04491
	16398	A_09_P022226	7.31462	-1.92062	3.976E-03	0.04606
CG17278	34660	A_09_P202905	10.73173	-2.34331	4.278E-05	0.00750
	10810	A_09_P057171	11.29221	-1.48129	5.790E-04	0.01853
CG17290	19108	A_09_P008631	4.23147	-2.25547	1.591E-03	0.03065
CG17321	13542	A_09_P023256	4.66238	-0.81730	2.597E-03	0.03797
CG17323	38180	A_09_P023241	7.57436	-1.28173	2.804E-03	0.03929
CG17574	17747	A_09_P007071	5.78798	-0.83861	2.268E-03	0.03604
CG17662	26073	A_09_P027016	4.99883	-1.01295	2.685E-03	0.03830
CG18157	30314	A_09_P149032	9.24886	-1.00228	1.220E-03	0.02683
CG18301	5368	A_09_P021666	4.78084	-1.51420	4.227E-03	0.04735
CG18304	4028	A_09_P202205	10.87457	-0.85714	3.647E-03	0.04434
CG18313	37426	A_09_P038665	8.20416	-2.18996	1.587E-05	0.00614
	33393	A_09_P038665	8.03757	-2.07557	2.663E-05	0.00686
CG18542	9518	A_09_P133175	9.18629	-0.81984	3.568E-03	0.04369
CG18547	33263	A_09_P074101	10.19940	-1.24606	9.243E-04	0.02338
	26127	A_09_P074101	10.10446	-1.06847	1.021E-03	0.02450
CG18585	11641	A_09_P020416	3.02224	-1.14297	3.326E-03	0.04235
CG1909	31966	A_09_P061886	7.05340	-1.14551	1.756E-03	0.03205
	21982	A_09_P061886	7.01136	-1.03204	2.854E-03	0.03956
CG2016	25799	A_09_P187190	8.53049	-1.45824	3.264E-03	0.04201

CG2022	22928	A_09_P036201	7.57262	-1.03372	2.056E-03	0.03460
	43339	A_09_P036201	7.42693	-0.95964	4.444E-03	0.04873
CG2556	20922	A_09_P038101	8.90481	-0.81244	3.230E-03	0.04180
CG2901	44064	A_09_P101370	5.44263	-1.21216	1.350E-03	0.02822
	34676	A_09_P066891	5.96112	-1.06555	1.900E-03	0.03334
CG30039	12884	A_09_P057411	3.92591	-0.94765	1.251E-03	0.02712
	26707	A_09_P057411	3.79642	-0.85197	2.669E-03	0.03830
CG30093	8454	A_09_P057611	7.11790	-0.88791	2.453E-03	0.03690
CG30104	8593	A_09_P057656	3.39809	-0.73907	4.656E-03	0.04989
CG30287	545	A_09_P058041	7.01228	-0.99944	3.309E-03	0.04224
CG30345	37110	A_09_P111075	9.15479	-1.46146	2.547E-04	0.01286
CG3097	42288	A_09_P067346	11.31937	-1.16121	7.955E-04	0.02163
CG31104	36783	A_09_P059011	3.28151	-1.56943	2.095E-04	0.01192
CG31106	5662	A_09_P059021	6.92299	-0.78996	3.742E-03	0.04464
CG31140	18699	A_09_P204740	7.78094	-1.42397	2.833E-03	0.03947
CG31221	10747	A_09_P045496	3.65768	-2.11973	2.784E-03	0.03911
CG31300	11284	A_09_P059556	2.53160	-1.19206	2.477E-03	0.03717
CG31475	2387	A_09_P059956	7.93811	-1.42974	1.058E-03	0.02496
	5617	A_09_P059956	8.15720	-1.36659	7.783E-04	0.02136
CG31522	40582	A_09_P109805	6.43843	-1.19273	4.771E-04	0.01682
CG31642	2819	A_09_P060306	10.07376	-2.07086	2.372E-04	0.01260
	8249	A_09_P060306	10.21858	-2.06184	2.004E-04	0.01178
CG31742	16440	A_09_P013601	9.12631	-1.06346	3.068E-03	0.04082
	27401	A_09_P013601	8.97583	-1.00085	3.782E-03	0.04477
CG31776	12918	A_09_P201415	7.69638	-1.50016	4.084E-04	0.01566
	40728	A_09_P013676	7.03093	-1.15141	6.531E-04	0.01965
CG31788	38916	A_09_P013726	3.89536	-1.47953	1.198E-03	0.02659
CG31816	31152	A_09_P166205	7.65514	-1.56049	3.956E-04	0.01544
	884	A_09_P013826	6.08085	-1.42332	1.483E-04	0.01048
CG31909	30527	A_09_P014086	2.45035	-1.57611	3.295E-04	0.01448
CG31997	8996	A_09_P014251	9.16790	-1.31963	5.314E-04	0.01772
	12018	A_09_P014251	9.23698	-1.31162	5.620E-04	0.01829
	3716	A_09_P193045	10.41241	-1.25567	2.411E-04	0.01261
CG32006	1914	A_09_P014276	6.48946	-1.00509	9.632E-04	0.02394
	26464	A_09_P014276	6.64728	-0.87563	1.626E-03	0.03104
CG32237	19066	A_09_P015011	3.26892	-1.23172	4.283E-03	0.04770
CG32264	20465	A_09_P185580	4.48117	-1.15662	1.568E-03	0.03039
	33561	A_09_P193010	8.88373	-0.85156	2.446E-03	0.03690
CG32354	36996	A_09_P015331	9.16963	-1.66304	1.075E-04	0.00943
	19936	A_09_P015331	9.18001	-1.53802	1.433E-04	0.01046
CG32459	21120	A_09_P015616	6.81844	-0.92231	3.702E-03	0.04460
	34270	A_09_P015616	6.93583	-0.90210	3.551E-03	0.04356
CG32588	36146	A_09_P015926	4.82207	-2.25863	1.938E-03	0.03363
	6272	A_09_P015926	4.79049	-1.97860	8.872E-05	0.00892
CG32638	25989	A_09_P016031	11.55956	-1.11399	4.733E-04	0.01674
	20854	A_09_P016031	11.59665	-0.93186	1.345E-03	0.02822
CG32695	18797	A_09_P016251	3.41164	-1.96537	4.906E-05	0.00755
CG33109	9307	A_09_P016836	3.95428	-1.35550	6.924E-04	0.02015
CG33128	10590	A_09_P215430	7.29777	-1.29868	2.388E-04	0.01261
	19429	A_09_P016901	7.16983	-1.20880	4.478E-04	0.01634
CG33144	14266	A_09_P139565	8.86723	-1.07697	7.626E-04	0.02115
	39779	A_09_P016941	8.94041	-1.06625	1.962E-03	0.03387
	37325	A_09_P016941	9.06182	-1.03446	1.369E-03	0.02826
CG33233	4421	A_09_P108210	6.27114	-1.20818	9.978E-04	0.02427
CG33285	42082	A_09_P017411	7.03771	-1.79491	1.634E-03	0.03106
	5293	A_09_P017411	7.11590	-1.69512	1.568E-03	0.03039
CG33287	28482	A_09_P017421	4.04472	-0.90321	2.689E-03	0.03830
CG33474	34212	A_09_P017661	8.28311	-1.17208	7.680E-04	0.02118
CG33798	44926	A_09_P000671	5.72773	-1.44957	2.790E-04	0.01335
	42305	A_09_P000671	5.69108	-1.28028	1.201E-03	0.02660
CG33912	17407	A_09_P000786	6.75927	-0.93891	2.554E-03	0.03772
	25225	A_09_P000786	6.81028	-0.80207	4.626E-03	0.04972
CG34107	2066	A_09_P002511	4.40017	-0.84222	2.796E-03	0.03921
CG34114	11698	A_09_P129855	7.97506	-1.32058	2.629E-03	0.03809
CG34160	22670	A_09_P002986	6.08654	-1.15298	4.699E-03	0.04995

CG34215	36962	A_09_P003246	4.42359	-2.42234	3.923E-05	0.00750
	7424	A_09_P003246	4.69399	-2.09060	2.275E-04	0.01245
CG34222	36102	A_09_P003281	9.43837	-1.39629	1.741E-03	0.03202
CG34232	12374	A_09_P100320	10.51052	-1.19136	2.653E-03	0.03825
CG34290	44472	A_09_P003621	9.53372	-1.23701	7.641E-04	0.02115
	21216	A_09_P003621	9.60610	-1.15345	9.886E-04	0.02417
CG34383	36656	A_09_P206085	12.97102	-1.45007	3.475E-04	0.01459
	11257	A_09_P171405	10.65921	-1.23467	7.538E-04	0.02104
	30729	A_09_P129800	9.71070	-0.95036	1.443E-03	0.02913
	18761	A_09_P004086	11.25712	-0.92454	1.196E-03	0.02657
CG34432	15365	A_09_P004421	6.97207	-0.99042	4.700E-03	0.04995
CG3624	33714	A_09_P026441	7.49858	-1.31288	8.976E-04	0.02299
	18220	A_09_P026441	7.46568	-1.13742	2.378E-03	0.03662
	43123	A_09_P206660	10.29931	-0.79646	3.439E-03	0.04292
CG3835	27514	A_09_P076456	7.32957	-3.09223	1.239E-05	0.00601
	37933	A_09_P076456	7.23064	-3.00266	4.916E-06	0.00581
CG3842	30715	A_09_P067601	7.45931	-0.87455	2.553E-03	0.03772
CG3927	32685	A_09_P026516	5.15553	-1.57561	8.621E-04	0.02258
CG40178	20982	A_09_P171490	7.70637	-1.11497	3.072E-03	0.04082
CG4115	9645	A_09_P074276	7.73587	-1.51337	6.467E-04	0.01958
CG41423	16283	A_09_P005291	7.27808	-0.98881	3.234E-03	0.04180
CG4164	34306	A_09_P017961	13.37762	-0.87213	3.728E-03	0.04464
CG42237	45084	A_09_P044326	9.51758	-1.01012	1.135E-03	0.02587
CG42256	21099	A_09_P015411	5.90517	-1.18851	1.708E-03	0.03195
	41766	A_09_P048051	6.16379	-1.03324	2.994E-03	0.04025
CG42347	5809	A_09_P057306	7.76294	-0.75108	4.709E-03	0.04999
CG42358	17005	A_09_P069126	12.56494	-1.86633	4.122E-05	0.00750
	20366	A_09_P069126	12.44052	-1.78543	3.691E-05	0.00750
CG42394	4803	A_09_P186280	9.50546	-1.13739	2.030E-03	0.03438
CG42450	43315	A_09_P221790	5.55569	-1.26411	1.462E-03	0.02926
	9132	A_09_P015786	9.10146	-1.15463	3.232E-03	0.04180
	22596	A_09_P015786	9.02550	-1.05145	2.261E-03	0.03604
CG42663	904	A_09_P209265	9.03775	-1.16545	1.237E-03	0.02702
CG42671	15040	A_09_P054886	8.09601	-0.95968	3.979E-03	0.04606
CG4271	5560	A_09_P018501	4.78274	-0.73721	3.775E-03	0.04475
CG42732	15478	A_09_P003971	9.66966	-2.22223	4.426E-04	0.01632
CG43143	20818	A_09_P090275	11.08602	-0.87209	2.265E-03	0.03604
CG43340	22520	A_09_P079786	5.10573	-0.99475	1.597E-03	0.03074
	32131	A_09_P079786	5.07933	-0.93347	1.933E-03	0.03360
CG43638	11226	A_09_P206605	5.26910	-0.99437	1.734E-03	0.03202
CG43737	32578	A_09_P038886	9.27642	-1.20893	3.027E-03	0.04053
	42012	A_09_P038886	9.14824	-1.15915	3.201E-03	0.04161
CG4434	42065	A_09_P070616	5.78437	-0.91098	3.088E-03	0.04092
CG4455	41762	A_09_P065241	9.31585	-1.10018	2.598E-03	0.03797
CG4596	9750	A_09_P073651	9.34207	-0.98031	1.107E-03	0.02556
	6542	A_09_P073651	9.23686	-0.83832	2.502E-03	0.03741
CG4597	16890	A_09_P052901	5.81805	-1.43717	8.302E-04	0.02206
CG4607	34226	A_09_P141515	6.69376	-1.51829	1.090E-03	0.02539
CG4680	38040	A_09_P033651	8.06394	-1.75738	4.712E-04	0.01673
CG4683	4085	A_09_P129860	6.32553	-1.41313	3.212E-03	0.04167
	10996	A_09_P073681	9.26201	-1.13185	1.780E-03	0.03209
CG4688	22304	A_09_P151205	5.05050	-1.41790	1.576E-03	0.03048
CG4757	35510	A_09_P079686	4.02167	-2.93886	1.009E-04	0.00935
	7242	A_09_P079686	4.16624	-2.85781	3.189E-04	0.01437
CG5048	31037	A_09_P055966	4.24077	-1.48341	1.057E-04	0.00943
	28864	A_09_P164790	6.99174	-1.47525	6.214E-04	0.01915
	25199	A_09_P055966	4.50397	-1.25220	1.482E-03	0.02955
CG5050	3016	A_09_P022941	9.39803	-0.97444	1.133E-03	0.02584
	18650	A_09_P022941	9.46575	-0.91569	1.760E-03	0.03209
CG5059	38442	A_09_P035161	10.23013	-1.19614	5.001E-04	0.01710
CG5075	32769	A_09_P022396	7.14078	-1.10470	1.721E-03	0.03196
	15288	A_09_P022396	7.09955	-1.02166	2.686E-03	0.03830
	35486	A_09_P022396	7.09196	-1.01163	2.587E-03	0.03794
	40830	A_09_P022396	7.03037	-1.01014	2.099E-03	0.03496
	42702	A_09_P022396	6.89134	-0.96561	4.006E-03	0.04631

	25411	A_09_P022396	6.93488	-0.94171	2.565E-03	0.03781
	32887	A_09_P022396	6.90839	-0.93666	4.684E-03	0.04995
	30426	A_09_P022396	6.91346	-0.92610	3.677E-03	0.04451
CG5322	8155	A_09_P021626	7.97654	-1.16767	4.204E-04	0.01584
	41703	A_09_P021626	7.55067	-1.10449	9.271E-04	0.02341
	18066	A_09_P111230	7.41450	-1.05653	6.104E-04	0.01907
CG5342	3283	A_09_P134465	6.78084	-1.38364	1.930E-04	0.01170
	2543	A_09_P073886	8.70756	-1.07623	8.661E-04	0.02262
	4272	A_09_P073886	8.84696	-0.85735	3.333E-03	0.04237
CG5455	8374	A_09_P071986	8.53231	-1.10631	1.747E-03	0.03204
	7792	A_09_P071986	8.55947	-1.05725	1.366E-03	0.02826
CG5614	1637	A_09_P142075	6.51151	-0.89529	2.646E-03	0.03821
	17213	A_09_P075561	7.00677	-0.82004	2.807E-03	0.03929
CG5707	19040	A_09_P078761	6.66691	-1.09819	3.781E-03	0.04477
	17610	A_09_P078761	6.52789	-1.02914	3.282E-03	0.04205
CG5715	4329	A_09_P218095	10.66398	-0.72497	4.325E-03	0.04794
CG5731	38939	A_09_P021376	10.01650	-0.83252	2.101E-03	0.03496
CG5804	23581	A_09_P054086	6.77334	-1.40739	1.148E-03	0.02604
	15548	A_09_P054086	6.61347	-1.32381	2.261E-03	0.03604
CG5835	35390	A_09_P069116	9.40840	-1.14149	5.696E-04	0.01842
	23378	A_09_P069106	9.56816	-1.12758	4.321E-04	0.01609
CG5869	13602	A_09_P065986	10.88148	-0.77496	3.728E-03	0.04464
	6974	A_09_P065986	10.78863	-0.75689	3.959E-03	0.04591
CG5945	26576	A_09_P022536	7.05751	-1.19096	3.411E-04	0.01453
CG6024	781	A_09_P055081	9.59676	-2.30837	2.969E-04	0.01375
CG6045	6034	A_09_P075516	9.43531	-1.22642	1.744E-03	0.03203
	21393	A_09_P075516	9.43078	-1.15387	1.541E-03	0.03025
CG6206	37642	A_09_P079826	10.30962	-1.51895	1.020E-03	0.02450
CG6231	9422	A_09_P148150	3.95043	-1.33263	1.515E-03	0.02997
CG6272	29318	A_09_P054796	8.63650	-1.11754	8.827E-04	0.02279
	40219	A_09_P054796	8.76674	-1.09382	8.117E-04	0.02178
CG6329	37001	A_09_P007421	5.65126	-0.92023	2.246E-03	0.03604
	6315	A_09_P007421	5.53222	-0.79496	4.481E-03	0.04890
CG6330	2632	A_09_P072126	5.81628	-2.94164	1.874E-04	0.01160
	19820	A_09_P072126	5.79005	-2.82792	1.677E-04	0.01111
CG6356	8838	A_09_P071031	5.38039	-1.20847	3.272E-04	0.01446
	43638	A_09_P071031	5.28351	-0.97693	1.322E-03	0.02790
CG6357	20211	A_09_P124315	7.66606	-1.60628	8.184E-05	0.00873
	30297	A_09_P007441	8.58483	-1.58770	6.727E-05	0.00827
	37307	A_09_P007441	8.48507	-1.57581	9.007E-05	0.00899
CG6520	6972	A_09_P008696	3.25629	-1.18898	3.411E-03	0.04275
	11212	A_09_P008696	3.78139	-0.73978	3.931E-03	0.04582
CG6790	19859	A_09_P073881	7.73978	-1.34524	7.798E-04	0.02136
CG6996	23466	A_09_P034941	7.23967	-1.25963	5.976E-04	0.01885
	24591	A_09_P034941	7.24482	-1.22653	6.875E-04	0.02009
CG7017	6654	A_09_P034946	3.15423	-1.28450	7.564E-04	0.02107
CG7407	4134	A_09_P035646	8.26116	-0.86729	2.491E-03	0.03729
CG7560	10775	A_09_P054921	4.16523	-1.23879	4.980E-04	0.01707
CG7884	5576	A_09_P040416	6.48760	-1.96175	6.939E-04	0.02015
	12776	A_09_P040416	6.41856	-1.94209	2.912E-04	0.01362
CG7900	5851	A_09_P217635	12.54812	-0.88520	4.090E-03	0.04669
CG7991	19870	A_09_P119080	6.44297	-1.52088	2.094E-04	0.01192
CG8369	32823	A_09_P063141	9.53533	-0.76466	3.801E-03	0.04484
CG8398	28214	A_09_P053301	5.59373	-1.02800	3.202E-03	0.04161
CG8420	10223	A_09_P072891	8.66906	-2.84710	2.453E-05	0.00683
	44215	A_09_P072891	8.40863	-2.83510	3.853E-05	0.00750
CG8620	19614	A_09_P063686	6.59863	-1.35733	2.476E-04	0.01274
CG8740	14897	A_09_P079691	4.55007	-2.31277	2.788E-03	0.03914
CG9084	34042	A_09_P006306	8.45401	-1.67917	5.808E-05	0.00798
CG9095	1736	A_09_P038876	5.19199	-1.40900	2.740E-04	0.01328
	37056	A_09_P038876	5.39060	-0.99779	3.425E-03	0.04283
CG9175	12091	A_09_P019866	8.37773	-0.85479	2.359E-03	0.03643
	25357	A_09_P019866	8.22191	-0.82138	2.160E-03	0.03546
CG9232	29737	A_09_P020086	5.87407	-2.05064	7.751E-04	0.02134
CG9297	41692	A_09_P194255	5.79180	-1.36935	3.470E-04	0.01459

CG9312	17314	A_09_P074856	6.24054	-1.37527	4.017E-03	0.04634
CG9426	23469	A_09_P022491	11.51114	-0.76252	3.129E-03	0.04112
	30924	A_09_P022491	11.38720	-0.75452	3.384E-03	0.04260
CG9445	5094	A_09_P063346	7.74214	-3.43625	1.353E-06	0.00581
CG9449	36111	A_09_P034606	5.01069	-2.17504	4.266E-03	0.04763
	34075	A_09_P034606	4.49801	-1.90978	1.768E-03	0.03209
CG9664	41677	A_09_P018906	5.07724	-1.26114	5.024E-04	0.01714
	5327	A_09_P018906	5.05429	-0.88981	2.249E-03	0.03604
CG9812	19688	A_09_P026921	3.27147	-1.23441	3.678E-04	0.01479
CG9894	43374	A_09_P221985	9.70659	-1.19585	8.467E-04	0.02233
	30216	A_09_P174270	11.58781	-1.07092	2.568E-03	0.03783
Chd64	12726	A_09_P029301	12.80236	-0.77317	4.598E-03	0.04962
CheA29a	10664	A_09_P017071	6.15368	-0.79957	3.316E-03	0.04231
CheA87a	19304	A_09_P074716	4.55191	-0.99457	2.685E-03	0.03830
cib	43888	A_09_P078106	13.85459	-1.08245	7.755E-04	0.02134
	28795	A_09_P078106	13.86793	-1.03783	1.207E-03	0.02665
	30843	A_09_P078106	13.78717	-1.02372	1.312E-03	0.02772
	13015	A_09_P078106	14.00194	-1.01303	1.174E-03	0.02639
	4534	A_09_P078106	13.94007	-0.99120	1.452E-03	0.02919
	16279	A_09_P078106	14.04834	-0.98940	1.542E-03	0.03025
	28585	A_09_P078106	13.89433	-0.98472	1.201E-03	0.02660
	30477	A_09_P078106	13.89919	-0.97991	1.257E-03	0.02712
	23224	A_09_P078106	13.91612	-0.97378	1.371E-03	0.02826
	17034	A_09_P078106	14.01375	-0.91357	1.566E-03	0.03039
cola	460	A_09_P130330	3.20483	-1.13964	4.609E-03	0.04967
cora	38106	A_09_P012506	6.50551	-0.91436	2.602E-03	0.03798
cpo	38544	A_09_P041801	9.07576	-1.04114	1.361E-03	0.02826
	5238	A_09_P225995	9.48040	-0.87310	3.069E-03	0.04082
cpx	43734	A_09_P064316	3.54707	-1.01397	2.377E-03	0.03661
	12609	A_09_P182675	7.33545	-0.91962	3.798E-03	0.04483
	20944	A_09_P193225	9.66432	-0.88348	1.633E-03	0.03106
	36183	A_09_P225125	4.05713	-0.70904	4.618E-03	0.04971
Crg-1	9015	A_09_P140588	10.82069	-0.99675	1.093E-03	0.02544
crol	7457	A_09_P032231	11.14958	-1.30207	5.938E-04	0.01879
	14488	A_09_P032231	11.18950	-1.24591	2.099E-03	0.03496
	35355	A_09_P050396	10.42314	-1.17333	6.420E-04	0.01949
	41556	A_09_P180365	15.21540	-1.08601	1.641E-03	0.03107
d	37465	A_09_P179220	9.32112	-1.16751	1.077E-03	0.02524
	21042	A_09_P041896	6.52121	-1.02931	1.345E-03	0.02822
dally	18003	A_09_P013216	5.01806	-1.46874	1.240E-03	0.02703
Dap160	10696	A_09_P209195	7.76448	-0.97650	4.075E-03	0.04661
daw	16336	A_09_P018706	2.96515	-1.18691	6.188E-04	0.01910
	35100	A_09_P018706	3.18207	-0.74905	3.695E-03	0.04459
Dgp-1	14582	A_09_P079961	11.27737	-0.90779	3.536E-03	0.04348
	36824	A_09_P079961	10.98864	-0.90741	2.294E-03	0.03628
Dh44-R1	2079	A_09_P007661	5.25401	-1.06786	1.196E-03	0.02657
dib	2189	A_09_P141385	6.66409	-0.89715	2.265E-03	0.03604
dnr1	6521	A_09_P026511	5.95545	-0.78512	4.513E-03	0.04910
Dok	837	A_09_P189630	11.26567	-1.03935	2.844E-03	0.03952
	17607	A_09_P067886	10.99733	-0.86782	2.452E-03	0.03690
drl	37836	A_09_P030801	9.48546	-0.80067	4.256E-03	0.04757
drongo	6441	A_09_P032206	12.93729	-0.77992	4.454E-03	0.04877
Drs	30832	A_09_P012336	8.27183	-0.96078	4.541E-03	0.04929
dtr	23006	A_09_P033201	7.05329	-0.81181	2.383E-03	0.03664
	23357	A_09_P033201	7.03406	-0.80001	2.927E-03	0.03999
Dys	7035	A_09_P045866	8.86648	-1.95607	1.813E-04	0.01142
	8007	A_09_P091350	8.09953	-1.11928	6.508E-04	0.01965
east	36404	A_09_P222800	9.93318	-0.82350	4.174E-03	0.04708
ec	44056	A_09_P042136	8.49828	-1.33940	3.785E-04	0.01508
	2214	A_09_P170860	8.81721	-1.19325	8.964E-04	0.02299
EcR	699	A_09_P206975	4.10534	-2.03116	4.310E-05	0.00750
	6362	A_09_P051976	8.48822	-1.69970	3.278E-04	0.01446
	4982	A_09_P042146	7.08126	-1.63556	1.450E-04	0.01046
	2439	A_09_P042146	7.04805	-1.57903	1.795E-04	0.01142
	21290	A_09_P050511	7.42977	-1.45707	4.218E-04	0.01586

Ect4	11376	A_09_P004026	11.82402	-0.86015	1.719E-03	0.03196
	22472	A_09_P143935	12.87628	-0.79395	3.112E-03	0.04102
	33776	A_09_P217320	11.36259	-0.77846	3.268E-03	0.04201
Eig71Ei	24978	A_09_P030366	3.79825	-2.19402	1.756E-03	0.03205
Eig71Ek	19788	A_09_P030376	4.06663	-2.68353	5.884E-04	0.01866
Eip75B	18320	A_09_P049541	6.42202	-1.50403	3.436E-04	0.01453
elav	32472	A_09_P090750	9.11339	-1.44977	1.356E-03	0.02826
	43452	A_09_P121080	9.90996	-1.02710	1.180E-03	0.02646
	21436	A_09_P042226	10.98460	-0.82425	3.665E-03	0.04449
	34863	A_09_P188635	10.46326	-0.74039	3.549E-03	0.04356
Eno	38188	A_09_P042251	12.72395	-0.92851	1.325E-03	0.02795
	1214	A_09_P042251	12.58621	-0.88286	1.714E-03	0.03195
esn	36969	A_09_P134125	6.11439	-2.07815	2.301E-03	0.03629
	7685	A_09_P065501	4.98600	-1.91756	2.432E-04	0.01261
Exn	24540	A_09_P178980	5.08401	-1.04695	2.330E-03	0.03641
	13420	A_09_P079726	7.05481	-0.90132	1.911E-03	0.03340
fax	37871	A_09_P126520	11.23197	-1.70837	9.115E-04	0.02326
	6109	A_09_P030211	10.92787	-1.35096	1.088E-03	0.02536
	23163	A_09_P030211	10.91131	-1.24386	1.070E-03	0.02516
	13780	A_09_P147645	11.28257	-1.04752	2.102E-03	0.03496
	43686	A_09_P050171	11.82772	-0.98311	1.778E-03	0.03209
Fhos	13217	A_09_P047936	10.31628	-1.87114	9.713E-05	0.00921
fj	27168	A_09_P042371	7.50761	-3.61694	2.872E-04	0.01353
	9710	A_09_P042371	7.61809	-3.56434	2.477E-04	0.01274
	20441	A_09_P121565	7.42725	-3.13010	2.594E-04	0.01293
fng	5908	A_09_P013246	6.35260	-2.82643	9.971E-05	0.00934
	6182	A_09_P013246	6.21931	-2.78101	2.044E-03	0.03447
for	4490	A_09_P042411	12.20350	-0.99296	1.527E-03	0.03005
	4302	A_09_P042411	12.17670	-0.93705	2.106E-03	0.03502
fs(1)K10	13816	A_09_P042426	13.06186	-4.18982	2.717E-07	0.00355
	27524	A_09_P042426	12.71973	-4.08884	2.874E-07	0.00355
fz3	28100	A_09_P079251	8.67381	-1.24004	1.258E-03	0.02712
	32287	A_09_P199375	9.85372	-0.94733	1.116E-03	0.02559
G-salpha60A	44066	A_09_P042581	9.40379	-1.06811	3.607E-03	0.04398
	12451	A_09_P042581	9.93966	-1.02183	1.559E-03	0.03039
	8655	A_09_P196460	11.26004	-0.78780	4.663E-03	0.04990
GABA-B-R1	27944	A_09_P187730	8.03808	-0.81283	2.411E-03	0.03686
galectin	16459	A_09_P122725	10.77362	-1.64053	8.815E-05	0.00890
	20671	A_09_P017791	10.84320	-1.63510	1.982E-04	0.01176
Gasp	15864	A_09_P078086	5.12534	-1.02314	8.965E-04	0.02299
gbb	44930	A_09_P076726	8.61786	-0.87591	1.791E-03	0.03218
	37890	A_09_P076726	8.70742	-0.82408	2.300E-03	0.03628
Gld2	10671	A_09_P070091	12.36162	-1.70886	8.726E-05	0.00888
	27585	A_09_P070091	11.94948	-1.49842	1.060E-04	0.00943
Gli	16331	A_09_P043156	7.00300	-1.53053	7.132E-04	0.02042
	6738	A_09_P043156	6.96633	-1.51010	1.450E-03	0.02919
	16041	A_09_P144150	7.27389	-1.12916	1.802E-03	0.03231
gol	25769	A_09_P146635	3.65548	-1.33090	3.328E-03	0.04235
	42537	A_09_P011351	3.54985	-1.28916	2.555E-03	0.03772
Gp150	33169	A_09_P116275	10.78072	-0.76400	4.660E-03	0.04990
	28300	A_09_P029566	10.59030	-0.75845	3.476E-03	0.04306
Gr28b	461	A_09_P056916	7.23797	-1.56624	5.511E-04	0.01811
	45007	A_09_P056916	7.60469	-1.41646	4.142E-04	0.01572
Gr63a	31028	A_09_P029176	10.98412	-1.02875	2.777E-03	0.03906
	42945	A_09_P029176	10.81373	-1.00455	3.550E-03	0.04356
Gr89a	3346	A_09_P075876	2.50834	-0.94199	1.630E-03	0.03104
Gr93a	17326	A_09_P064141	8.31901	-1.19553	5.164E-04	0.01748
	1808	A_09_P064141	8.43489	-1.02000	2.614E-03	0.03802
Gr93b	23739	A_09_P056851	9.05567	-1.48989	1.663E-03	0.03127
	34876	A_09_P056851	9.09191	-1.47505	2.118E-03	0.03510
Gr93c	34776	A_09_P056846	6.55886	-0.76206	3.515E-03	0.04333
Gr93d	7087	A_09_P056841	7.35028	-1.24365	1.359E-03	0.02826
	31432	A_09_P056841	7.27420	-1.16462	1.892E-03	0.03334
GstD1	757	A_09_P042646	11.58995	-3.24550	9.493E-06	0.00581
	27083	A_09_P042646	11.54485	-3.05871	7.737E-06	0.00581

GstD10	24830	A_09_P064681	9.20613	-3.53546	3.932E-05	0.00750
	10299	A_09_P209530	9.91909	-3.51265	1.119E-04	0.00954
GstD2	35205	A_09_P147905	6.41932	-1.80135	1.282E-04	0.01012
	17233	A_09_P011881	6.40633	-1.61463	2.545E-04	0.01286
	30486	A_09_P011881	6.59851	-1.52136	2.990E-04	0.01378
GstD3	24990	A_09_P011886	9.59964	-1.57554	1.740E-03	0.03202
	24441	A_09_P011886	9.58407	-1.42563	3.471E-03	0.04305
GstD4	3955	A_09_P011891	7.05243	-0.93561	2.122E-03	0.03511
	42966	A_09_P011891	7.01517	-0.88092	2.101E-03	0.03496
GstD6	11266	A_09_P011901	5.36996	-1.46456	2.019E-04	0.01183
	9812	A_09_P011901	5.34157	-1.33014	2.062E-03	0.03464
GstD9	36371	A_09_P074286	11.69442	-1.92309	4.262E-05	0.00750
GstE5	8378	A_09_P001981	10.61917	-2.20438	2.972E-04	0.01375
	14178	A_09_P001981	10.53837	-2.18645	1.483E-04	0.01048
	26666	A_09_P110960	9.01981	-1.88829	3.374E-04	0.01453
GstE6	15967	A_09_P001976	9.32970	-2.74495	2.066E-05	0.00643
	1571	A_09_P001976	9.12311	-2.55184	5.402E-05	0.00782
GstE7	6218	A_09_P001971	7.96691	-1.93729	3.405E-05	0.00750
	39291	A_09_P001971	7.99043	-1.91433	4.784E-05	0.00755
GstE8	24607	A_09_P001966	6.04514	-0.88358	3.112E-03	0.04102
GstE9	27135	A_09_P001961	6.87753	-1.02802	9.852E-04	0.02413
	15744	A_09_P001961	6.92189	-0.91952	1.267E-03	0.02723
H2.0	28508	A_09_P042671	5.27467	-1.00421	2.337E-03	0.03643
Hen1	3873	A_09_P006676	11.16825	-0.79370	2.835E-03	0.03947
hig	40394	A_09_P011971	6.29173	-2.04159	2.542E-03	0.03771
	724	A_09_P011971	6.18641	-1.87755	1.373E-03	0.02826
hipk	34739	A_09_P188060	12.52184	-0.98930	2.921E-03	0.03996
	35996	A_09_P028001	11.23458	-0.98854	2.058E-03	0.03462
	19159	A_09_P028001	11.16299	-0.70545	4.710E-03	0.04999
HLH4C	35401	A_09_P013011	3.57004	-0.79654	3.023E-03	0.04053
HLHm7	35451	A_09_P180645	10.62556	-1.22515	3.599E-04	0.01468
Hsp22	40043	A_09_P042786	12.20019	-0.80120	2.416E-03	0.03686
	22406	A_09_P042786	12.08706	-0.71719	4.113E-03	0.04687
Hsp67Bb	153	A_09_P042811	10.22583	-1.41031	3.335E-04	0.01453
	38440	A_09_P042811	10.13741	-1.23231	3.372E-04	0.01453
Hsp70Bb	17820	A_09_P029581	12.33108	-0.93044	4.683E-03	0.04995
Idgf4	40411	A_09_P078611	9.57545	-0.78771	3.093E-03	0.04092
Ilp2	32637	A_09_P054556	4.07156	-2.47422	1.523E-04	0.01058
	44657	A_09_P054556	3.69855	-1.67805	2.225E-03	0.03598
Invadolysin	43029	A_09_P107150	7.90579	-0.87674	3.405E-03	0.04274
inx2	381	A_09_P079136	12.03965	-0.80673	2.554E-03	0.03772
	5075	A_09_P079136	12.24264	-0.77645	2.869E-03	0.03966
inx3	9408	A_09_P064886	9.50894	-1.10467	2.420E-03	0.03686
inx7	2902	A_09_P079121	5.45995	-1.58112	1.564E-03	0.03039
	44046	A_09_P177915	6.64262	-1.29224	6.543E-04	0.01965
IP3K1	2976	A_09_P108475	10.12336	-0.93673	4.479E-03	0.04890
Ir52c	19727	A_09_P058561	5.14578	-0.88815	2.296E-03	0.03628
	13948	A_09_P058561	5.32045	-0.77675	3.846E-03	0.04514
jar	44306	A_09_P012921	8.05114	-0.80918	2.427E-03	0.03690
JhI-26	9433	A_09_P065051	10.10602	-1.72481	2.043E-04	0.01192
kek5	37568	A_09_P040471	12.20225	-1.16033	3.714E-03	0.04463
	11922	A_09_P172690	10.48371	-0.94266	3.881E-03	0.04540
Kmn1	37594	A_09_P079151	9.63692	-1.80280	6.550E-05	0.00827
	12874	A_09_P079151	9.91900	-1.76358	3.994E-05	0.00750
kni	9631	A_09_P042996	4.58547	-1.03552	1.222E-03	0.02685
Kr	32332	A_09_P043016	6.28931	-1.15090	4.173E-03	0.04708
l(2)01810	28619	A_09_P012596	8.99490	-0.81033	4.403E-03	0.04852
l(3)neo38	13946	A_09_P050936	13.92706	-0.97969	1.084E-03	0.02534
	36780	A_09_P116250	11.34568	-0.93033	1.866E-03	0.03304
	22967	A_09_P203805	8.57574	-0.84514	3.190E-03	0.04151
	42411	A_09_P126260	10.98347	-0.80607	3.830E-03	0.04504
lama	31816	A_09_P031426	7.53789	-1.52737	5.404E-04	0.01786
	37163	A_09_P031426	7.47802	-1.49108	1.002E-03	0.02433
LanA	17648	A_09_P043306	9.92497	-1.67382	6.627E-05	0.00827
LanB1	15218	A_09_P043311	11.39461	-0.76661	4.033E-03	0.04642

	40410	A_09_P043311	11.25867	-0.75271	3.330E-03	0.04236
laza	7462	A_09_P035741	7.56613	-2.14437	1.445E-03	0.02913
	27231	A_09_P140075	7.55825	-1.90329	1.232E-03	0.02695
lbm	10847	A_09_P031431	8.57978	-1.30719	2.068E-04	0.01192
	28657	A_09_P031431	8.19054	-1.22040	5.806E-04	0.01853
lea	18419	A_09_P043346	8.33884	-2.35423	1.010E-05	0.00581
	25827	A_09_P043346	8.35260	-2.17133	2.506E-05	0.00683
	32337	A_09_P133790	7.04426	-1.34191	4.135E-04	0.01571
lectin-28C	8082	A_09_P115030	4.31620	-0.91463	2.646E-03	0.03821
Letm1	34521	A_09_P117140	12.49770	-0.90119	1.838E-03	0.03277
Lip3	44822	A_09_P076441	6.12869	-1.22399	4.291E-03	0.04771
Lk6	25726	A_09_P117490	13.41238	-0.81989	2.381E-03	0.03663
	32715	A_09_P031896	12.73432	-0.71686	4.696E-03	0.04995
lmd	19930	A_09_P070481	3.33287	-1.07668	2.623E-03	0.03809
Lsp1beta	41675	A_09_P043366	8.06663	-1.05277	1.016E-03	0.02450
	45082	A_09_P043366	7.94583	-1.01574	2.249E-03	0.03604
mbl	23879	A_09_P017096	9.61426	-1.13137	2.375E-03	0.03661
Mdr65	44895	A_09_P010666	5.68309	-1.74293	4.273E-05	0.00750
melt	5535	A_09_P045646	7.21245	-1.07739	2.662E-03	0.03829
Men	5584	A_09_P043541	12.15457	-2.28426	8.373E-06	0.00581
	31172	A_09_P091290	9.63463	-2.26774	2.652E-05	0.00686
	15773	A_09_P090755	12.82783	-1.21536	4.445E-04	0.01633
miple2	10819	A_09_P066351	9.83735	-1.75957	6.508E-05	0.00827
	2898	A_09_P066351	9.44061	-1.67446	1.394E-04	0.01036
mnd	36460	A_09_P154825	8.71909	-1.59167	2.422E-04	0.01261
	1709	A_09_P043611	8.11153	-1.53481	3.135E-04	0.01419
	26687	A_09_P043611	8.23566	-1.51396	3.409E-04	0.01453
Mocs1	41803	A_09_P045656	10.35880	-0.70257	4.611E-03	0.04967
modSP	36166	A_09_P059336	9.77331	-2.18101	5.335E-05	0.00782
	27668	A_09_P059336	9.62790	-1.85587	1.022E-04	0.00938
	4568	A_09_P198635	8.39199	-1.45276	1.583E-04	0.01068
Msp-300	4398	A_09_P050461	9.74806	-2.34335	1.391E-04	0.01036
	20893	A_09_P050976	5.91635	-1.79670	4.729E-05	0.00755
	38664	A_09_P166080	9.19804	-1.28222	4.356E-03	0.04821
	29967	A_09_P046011	8.35749	-1.05968	5.983E-04	0.01885
msta	15352	A_09_P000246	6.55871	-2.22834	1.100E-04	0.00952
mthl3	40262	A_09_P066206	9.14810	-1.96594	2.221E-04	0.01233
	23804	A_09_P066206	9.25255	-1.87168	2.925E-04	0.01365
mthl4	43920	A_09_P008681	7.54751	-2.21730	2.289E-04	0.01246
	8691	A_09_P008681	7.84328	-2.04591	1.247E-04	0.01005
mthl5	39585	A_09_P074051	7.19831	-1.28532	2.076E-04	0.01192
	44144	A_09_P074051	7.28827	-1.20442	6.112E-04	0.01907
MtnD	15271	A_09_P017061	8.09832	-1.08653	4.392E-03	0.04847
Myo10A	14782	A_09_P037516	7.42853	-1.15140	2.088E-03	0.03496
myoglianin	9219	A_09_P078306	9.94748	-1.02420	1.638E-03	0.03107
	24237	A_09_P078306	9.74340	-0.96218	1.153E-03	0.02606
mys	43489	A_09_P010991	10.45109	-0.95414	2.018E-03	0.03429
	43249	A_09_P010991	10.45271	-0.95325	1.710E-03	0.03195
N	8672	A_09_P010921	11.17435	-1.16324	6.703E-04	0.01986
	11845	A_09_P050251	12.95176	-0.90767	1.928E-03	0.03358
nan	5444	A_09_P213845	5.86420	-0.96763	2.593E-03	0.03797
ND75	36896	A_09_P140975	8.17926	-0.88963	3.687E-03	0.04458
Ndae1	17784	A_09_P020306	11.00919	-0.84962	2.060E-03	0.03463
Nep3	24988	A_09_P040711	8.18470	-1.47924	1.507E-04	0.01054
	4148	A_09_P040711	8.40669	-1.44837	1.772E-04	0.01142
	7412	A_09_P143385	9.11908	-1.20898	6.164E-04	0.01910
nerfin-1	38817	A_09_P066346	6.71356	-1.00123	3.554E-03	0.04357
net	40137	A_09_P043836	5.64419	-2.02996	4.439E-05	0.00750
	22128	A_09_P043836	5.44376	-1.64255	5.564E-05	0.00790
	33254	A_09_P104200	6.36166	-1.27159	4.183E-04	0.01582
Nhe2	43639	A_09_P189445	9.17104	-1.25444	2.599E-04	0.01293
	43111	A_09_P045726	9.94160	-1.24269	2.776E-04	0.01335
ninaC	39722	A_09_P043866	6.28809	-0.86659	3.675E-03	0.04451
noc	33758	A_09_P011806	9.40192	-2.53779	1.349E-05	0.00614
	22078	A_09_P011806	9.41328	-2.51875	1.141E-05	0.00591

nompC	38851	A_09_P031696	5.41500	-1.27548	2.644E-03	0.03821
	8951	A_09_P031696	5.49832	-1.23579	2.146E-03	0.03537
Nplp2	26788	A_09_P047146	5.41449	-1.03202	2.215E-03	0.03592
nullo	7651	A_09_P010186	4.18239	-0.94278	4.126E-03	0.04693
Oatp30B	38295	A_09_P021126	8.24147	-1.22287	1.330E-03	0.02800
	38992	A_09_P021126	8.12951	-1.19140	1.022E-03	0.02450
obst-F	28586	A_09_P034926	6.88305	-1.86919	3.445E-04	0.01453
	5803	A_09_P034926	6.75200	-1.65487	2.738E-04	0.01328
Octbeta3R	19818	A_09_P169470	6.89122	-0.76644	3.791E-03	0.04480
odd	22692	A_09_P131435	4.36396	-1.66619	1.418E-04	0.01044
ogre	16526	A_09_P143495	9.09269	-1.80040	4.850E-04	0.01690
	36047	A_09_P010916	9.96330	-1.64101	1.423E-04	0.01044
	10474	A_09_P010916	9.96528	-1.55128	2.180E-04	0.01220
	33570	A_09_P135225	9.21834	-1.31887	3.069E-04	0.01401
Or46a	36621	A_09_P078521	8.97879	-0.94424	2.831E-03	0.03947
Orct2	35907	A_09_P048681	7.15888	-1.44520	1.216E-04	0.00996
	18219	A_09_P048681	7.11992	-1.35095	4.108E-04	0.01569
ort	12046	A_09_P105285	4.12713	-0.87729	4.110E-03	0.04687
pain	26353	A_09_P187395	10.98216	-1.34954	2.409E-04	0.01261
	44683	A_09_P181735	11.03971	-1.07581	1.108E-03	0.02556
	1728	A_09_P001821	8.02197	-1.02950	1.031E-03	0.02456
	4614	A_09_P001821	8.09134	-0.98624	9.763E-04	0.02401
pb	29130	A_09_P151010	3.50519	-1.58085	8.839E-04	0.02280
Peb	11395	A_09_P010261	4.80339	-1.02267	4.129E-03	0.04695
PGRP-LB	34279	A_09_P073846	8.35492	-0.93702	1.460E-03	0.02924
pirk	34801	A_09_P207530	6.62042	-2.04620	1.728E-05	0.00614
	19726	A_09_P215370	6.49797	-1.88535	3.534E-05	0.00750
	11335	A_09_P026156	6.65859	-1.79171	3.357E-05	0.00750
	16545	A_09_P026156	6.49433	-1.76346	5.391E-05	0.00782
Pka-C3	42040	A_09_P042051	7.18318	-2.44537	3.677E-04	0.01479
	12679	A_09_P042051	7.46548	-2.36863	1.066E-04	0.00943
Pkd2	4933	A_09_P064111	6.67184	-1.99881	1.350E-03	0.02822
	16072	A_09_P064111	6.69054	-1.63570	1.512E-03	0.02996
pnt	24625	A_09_P135145	11.99813	-1.04643	1.734E-03	0.03202
	62	A_09_P044171	11.69586	-1.03854	2.453E-03	0.03690
	31865	A_09_P142265	6.84701	-1.02848	2.447E-03	0.03690
	42973	A_09_P044171	11.46261	-0.93832	3.730E-03	0.04464
	31510	A_09_P044171	11.55923	-0.91810	4.134E-03	0.04695
	4671	A_09_P044171	11.69422	-0.90096	4.424E-03	0.04858
	42528	A_09_P044171	11.50705	-0.87351	3.671E-03	0.04451
pot	27576	A_09_P128510	5.05922	-1.98073	4.324E-04	0.01609
Pp1-13C	11842	A_09_P044191	7.20401	-1.04255	1.511E-03	0.02995
ppk28	10518	A_09_P039591	8.15886	-1.00060	2.921E-03	0.03996
Ppm1	41625	A_09_P028006	8.69482	-1.43473	3.165E-04	0.01429
PPYR1	25966	A_09_P038906	5.05176	-3.26902	3.635E-04	0.01472
	42054	A_09_P038906	4.95152	-2.95119	9.131E-04	0.02326
Prx2540-2	3483	A_09_P006101	7.78587	-1.16853	4.906E-04	0.01698
ps	40458	A_09_P193630	9.86568	-1.29925	6.950E-04	0.02016
	20817	A_09_P078251	9.56211	-1.08292	1.418E-03	0.02876
	7683	A_09_P078251	9.55519	-1.03102	1.250E-03	0.02712
	38290	A_09_P078256	8.74309	-0.95649	3.725E-03	0.04464
	33554	A_09_P179165	9.33193	-0.94287	3.239E-03	0.04185
	8422	A_09_P136435	9.14904	-0.81659	4.249E-03	0.04753
Ptp61F	35402	A_09_P203075	11.83269	-0.85708	3.477E-03	0.04306
puc	39500	A_09_P010281	11.10405	-0.90762	2.877E-03	0.03971
pum	32261	A_09_P181770	11.77562	-1.43363	6.800E-04	0.02000
	32660	A_09_P044261	12.13984	-0.82234	2.923E-03	0.03996
Pvf2	31772	A_09_P020266	7.07312	-2.37585	1.805E-05	0.00620
	34312	A_09_P020266	7.13291	-2.32889	1.379E-05	0.00614
	24728	A_09_P121465	7.63239	-2.10307	3.623E-05	0.00750
Pvf3	21703	A_09_P121460	7.64135	-2.46619	1.929E-04	0.01170
	671	A_09_P164230	10.03467	-1.39452	1.958E-04	0.01174
pyd3	23787	A_09_P037061	5.42215	-1.09299	6.083E-04	0.01907
	6658	A_09_P037061	5.28840	-0.97671	1.234E-03	0.02697
qtc	27132	A_09_P065466	6.46765	-1.02832	1.085E-03	0.02534

	9278	A_09_P065466	6.65745	-1.01055	2.510E-03	0.03746
	19137	A_09_P153140	6.04700	-0.94472	1.118E-03	0.02560
qvr	16177	A_09_P017656	6.74426	-1.18220	3.754E-03	0.04465
	30762	A_09_P017656	6.61776	-1.03999	3.093E-03	0.04092
r-cup	31560	A_09_P040936	7.20157	-1.15259	1.310E-03	0.02769
	14974	A_09_P040936	7.23848	-0.97798	4.210E-03	0.04733
Rab8	3580	A_09_P134980	10.02637	-0.77026	3.569E-03	0.04369
Rab9Fb	19012	A_09_P016151	7.99575	-0.96035	1.492E-03	0.02964
RabX5	42480	A_09_P112940	6.26567	-2.62546	5.043E-05	0.00761
	42951	A_09_P028446	7.30045	-2.01230	5.574E-05	0.00790
ref(2)P	12557	A_09_P044376	12.07904	-0.93073	2.359E-03	0.03643
	22119	A_09_P044376	11.83568	-0.85735	2.442E-03	0.03690
	8035	A_09_P198585	11.97686	-0.75514	3.731E-03	0.04464
Rh7	15121	A_09_P055306	6.97771	-0.79774	3.270E-03	0.04201
RpL10Aa	23993	A_09_P075236	4.05869	-0.84138	2.419E-03	0.03686
rpr	44758	A_09_P013426	8.10164	-2.12707	2.603E-04	0.01293
Rtnl1	26584	A_09_P016851	10.99068	-0.87832	2.989E-03	0.04025
	6145	A_09_P136610	9.54918	-0.79158	3.392E-03	0.04263
Rya-r44F	13843	A_09_P102745	7.64266	-1.67076	2.973E-04	0.01375
scb	29576	A_09_P044561	8.66924	-1.53879	1.564E-04	0.01068
	4119	A_09_P187265	5.95267	-1.52600	2.842E-04	0.01344
	7101	A_09_P044561	8.55951	-1.50092	3.976E-04	0.01549
scyl	19463	A_09_P224725	10.14757	-1.03698	1.113E-03	0.02557
	34761	A_09_P063976	10.12366	-0.96065	1.789E-03	0.03218
sda	19914	A_09_P030891	9.48474	-2.70213	7.562E-06	0.00581
	22081	A_09_P030891	9.43230	-2.61213	5.574E-06	0.00581
Sema-5c	24018	A_09_P065586	9.32901	-1.41151	3.900E-04	0.01531
	42804	A_09_P065586	9.25579	-1.32334	2.819E-04	0.01338
shep	36181	A_09_P205975	11.37792	-1.21803	2.436E-03	0.03690
shn	27055	A_09_P044726	11.07687	-0.97766	2.079E-03	0.03490
	533	A_09_P044726	10.93640	-0.96268	3.271E-03	0.04201
shot	29507	A_09_P047301	11.91381	-1.65685	8.180E-05	0.00873
sif	33444	A_09_P134315	10.69437	-1.41319	8.847E-04	0.02280
	28896	A_09_P004331	10.46332	-1.38953	3.094E-03	0.04092
	14467	A_09_P004336	7.56134	-0.93219	2.409E-03	0.03686
skl	28876	A_09_P034256	5.84660	-1.73344	1.308E-04	0.01014
	18047	A_09_P034256	5.94726	-1.61793	1.008E-04	0.00935
sli	44588	A_09_P047561	10.02474	-0.77825	3.453E-03	0.04299
slo	17131	A_09_P044766	5.38091	-1.30773	8.254E-04	0.02201
slou	37586	A_09_P043881	6.19328	-0.92374	3.138E-03	0.04114
sls	38881	A_09_P045221	7.23874	-1.20142	4.365E-03	0.04825
sm	21501	A_09_P192270	7.77784	-1.92418	4.116E-03	0.04689
	573	A_09_P187005	8.44725	-1.56684	3.483E-03	0.04307
	17355	A_09_P209295	6.95469	-1.55394	2.311E-03	0.03637
	41100	A_09_P049316	9.04072	-1.27099	5.536E-04	0.01816
	33261	A_09_P044786	9.88203	-1.21019	1.803E-03	0.03231
	18118	A_09_P044786	9.87680	-1.15269	1.990E-03	0.03404
sns	15344	A_09_P076681	7.66061	-0.94094	1.754E-03	0.03205
	19232	A_09_P171072	7.88512	-0.91672	1.846E-03	0.03285
	42018	A_09_P076681	7.45547	-0.89047	3.276E-03	0.04201
Socs36E	36849	A_09_P045976	9.36027	-2.60895	4.833E-05	0.00755
Sox14	5155	A_09_P148280	6.30029	-1.17291	2.921E-03	0.03996
Sox15	28541	A_09_P011551	7.42397	-1.30924	1.867E-03	0.03304
Spn100A	57	A_09_P061456	7.58661	-0.85500	2.181E-03	0.03564
step	585	A_09_P202550	8.06809	-0.93677	2.448E-03	0.03690
stv	3913	A_09_P014671	8.04322	-0.76865	4.422E-03	0.04858
sty	20530	A_09_P196120	10.00320	-0.75112	3.725E-03	0.04464
su(r)	7675	A_09_P150565	5.37519	-1.40100	8.637E-04	0.02259
Su(z)2	44664	A_09_P011861	9.99590	-1.68891	4.176E-04	0.01582
	26605	A_09_P011861	10.11752	-1.62534	3.742E-04	0.01496
Sxl	12810	A_09_P143460	7.62724	-1.14803	4.709E-03	0.04999
Syt1	26229	A_09_P049161	6.27245	-1.36484	1.728E-04	0.01129
tai	12133	A_09_P063971	9.89384	-1.13179	1.647E-03	0.03111
	7514	A_09_P174985	8.25210	-0.99807	2.609E-03	0.03802
tal-AA	5512	A_09_P194030	9.34811	-2.13178	7.524E-05	0.00850

	22980	A_09_P184935	7.42769	-1.54079	3.628E-04	0.01472
TepII	5399	A_09_P213450	7.51882	-2.19783	3.480E-04	0.01459
	3608	A_09_P064071	7.94086	-1.89323	1.817E-04	0.01142
	6660	A_09_P064071	7.94118	-1.81457	2.742E-04	0.01328
TepIV	43713	A_09_P064061	10.69753	-1.10369	1.078E-03	0.02524
	9082	A_09_P064061	11.03932	-1.04599	1.550E-03	0.03031
Thor	14242	A_09_P032876	11.08811	-1.67839	7.320E-05	0.00848
	19101	A_09_P032876	10.89855	-1.62878	6.153E-05	0.00827
Tim13	37592	A_09_P055096	8.90826	-1.00232	1.393E-03	0.02851
Tim17a1	41967	A_09_P074281	8.51074	-4.04326	1.303E-04	0.01014
	12462	A_09_P074281	9.00748	-3.99853	8.628E-05	0.00882
	13907	A_09_P126325	6.23859	-2.78614	3.756E-06	0.00581
Tollo	6312	A_09_P116390	5.99776	-2.38678	2.201E-05	0.00656
	34858	A_09_P191795	8.67398	-2.22740	1.896E-05	0.00634
	1575	A_09_P045751	6.54969	-1.75260	4.894E-04	0.01698
Traf-like	9333	A_09_P039416	9.64304	-0.82646	2.849E-03	0.03956
	3853	A_09_P039416	9.42143	-0.76865	4.161E-03	0.04702
Tsp33B	3750	A_09_P022066	8.61490	-0.73788	4.405E-03	0.04852
Tsp42Ed	44443	A_09_P066596	8.99370	-1.55659	7.255E-04	0.02054
	29603	A_09_P066596	9.21457	-1.49146	3.602E-04	0.01468
tw	9814	A_09_P048691	8.81363	-1.04726	1.013E-03	0.02447
	898	A_09_P048691	8.57145	-0.99399	1.493E-03	0.02964
	19556	A_09_P048691	8.61275	-0.98143	1.409E-03	0.02862
	37239	A_09_P048691	8.67357	-0.95082	1.970E-03	0.03389
	35814	A_09_P048691	8.63806	-0.92867	1.478E-03	0.02950
	23014	A_09_P048691	8.65574	-0.92761	1.739E-03	0.03202
	13262	A_09_P048691	8.60253	-0.92128	1.995E-03	0.03404
	4101	A_09_P048691	8.53385	-0.86564	2.162E-03	0.03546
	17438	A_09_P048691	8.63300	-0.86119	3.088E-03	0.04092
	24847	A_09_P048691	8.58806	-0.85789	4.162E-03	0.04702
Ugt35a	7652	A_09_P078396	7.52996	-1.15079	4.810E-04	0.01686
	44846	A_09_P078396	7.48718	-1.01616	1.912E-03	0.03340
Ugt86Da	24564	A_09_P062526	8.95604	-3.76931	1.169E-06	0.00581
Ugt86De	42358	A_09_P062511	4.97721	-3.99252	3.072E-05	0.00710
	1632	A_09_P062511	4.93108	-3.69684	1.452E-05	0.00614
Ugt86Di	20314	A_09_P113560	7.11042	-1.77875	5.680E-04	0.01842
	17529	A_09_P062496	7.41694	-1.69719	1.747E-04	0.01137
	24186	A_09_P062496	7.44014	-1.40360	2.228E-04	0.01233
unc-115	21073	A_09_P073171	6.23127	-0.95961	2.165E-03	0.03547
unc-5	44816	A_09_P179550	10.66941	-1.29994	1.981E-04	0.01176
unc79	19106	A_09_P198005	6.20307	-1.82321	4.914E-05	0.00755
Uro	13933	A_09_P009961	3.60787	-1.61521	2.782E-04	0.01335
	18890	A_09_P009961	3.59853	-1.00972	1.047E-03	0.02480
VAChT	22496	A_09_P205590	6.34180	-0.98122	1.187E-03	0.02653
veil	26548	A_09_P008701	6.60129	-1.15160	1.119E-03	0.02560
vg	33773	A_09_P009991	7.78734	-1.66807	2.240E-03	0.03603
	15442	A_09_P009991	7.87826	-1.58502	2.285E-03	0.03628
Vinc	31228	A_09_P010486	8.44394	-1.21195	3.116E-03	0.04105
	7399	A_09_P010486	8.29457	-1.14002	3.573E-03	0.04371
vir-1	10243	A_09_P056621	6.55549	-1.18053	2.287E-03	0.03628
	33815	A_09_P056616	8.07456	-1.09626	2.324E-03	0.03637
	41688	A_09_P056616	8.04669	-1.05387	2.352E-03	0.03643
vri	7188	A_09_P031491	6.52748	-1.10748	4.683E-03	0.04995
w	42415	A_09_P046191	13.51650	-3.42847	5.532E-05	0.00790
	41490	A_09_P010031	15.15003	-3.36391	1.684E-06	0.00581
wb	4966	A_09_P171275	7.87952	-1.40218	4.980E-04	0.01707
wnd	33108	A_09_P034701	7.93760	-1.11434	8.546E-04	0.02247
Wnt2	6466	A_09_P010341	7.22725	-1.16534	1.288E-03	0.02748
Xbp1	14073	A_09_P196055	13.15363	-0.81107	3.691E-03	0.04458
	13921	A_09_P032791	12.28857	-0.78104	4.228E-03	0.04735
Xrp1	7259	A_09_P069036	9.78726	-1.72890	2.129E-04	0.01208
	30887	A_09_P184380	11.35486	-1.70136	7.199E-04	0.02050
	616	A_09_P069036	9.75804	-1.64669	2.801E-04	0.01338
yellow-h	38797	A_09_P061826	8.91118	-1.00716	1.053E-03	0.02491
	33119	A_09_P061826	8.96728	-0.97695	1.057E-03	0.02494

yrt	44012	A_09_P116530	10.03677	-0.83772	4.214E-03	0.04733
Z600	14931	A_09_P010096	6.15506	-1.29867	9.491E-04	0.02365
	42539	A_09_P010096	5.77940	-1.26714	1.934E-03	0.03360

Supplementary Table S3. The top 10 GO categories enriched in the genes down-regulated in Ovo-B KD PGCs

Biological Process

Term	Name	count*	p-value	q-value
GO:0048619	embryonic hindgut morphogenesis	6	1.2E-03	4.0E-01
GO:0048567	ectodermal gut morphogenesis	6	6.0E-03	4.0E-01
GO:0007439	ectodermal gut development	6	6.0E-03	4.0E-01
GO:0048546	gut morphogenesis	6	6.6E-03	4.1E-01
GO:0009309	amine biosynthetic process	6	6.6E-03	4.1E-01
GO:0035239	tube morphogenesis	8	5.4E-03	4.2E-01
GO:0008652	cellular amino acid biosynthetic process	5	5.9E-03	4.2E-01
GO:0048546	digestive tract morphogenesis	6	7.2E-03	4.2E-01
GO:0055123	digestive system development	6	7.2E-03	4.2E-01
GO:0006270	DNA replication initiation	5	4.5E-04	4.4E-01

Molecular Function

Term	Name	count*	p-value	q-value
GO:0043138	3'-5' DNA helicase activity	4	3.7E-03	6.0E-01
GO:0030246	carbohydrate binding	14	2.3E-03	6.7E-01
GO:0030247	polysaccharide binding	10	7.0E-03	6.8E-01
GO:0001871	pattern binding	10	7.0E-03	6.8E-01
GO:0003824	catalytic activity	129	2.5E-02	8.3E-01
GO:0003678	DNA helicase activity	5	2.3E-02	8.5E-01
GO:0008061	chitin binding	8	2.0E-02	8.6E-01
GO:0016638	oxidoreductase activity, acting on the CH-NH2 group of donors	3	4.8E-02	8.9E-01
GO:0004497	monooxygenase activity	8	4.0E-02	8.9E-01
GO:0042578	phosphoric ester hydrolase activity	11	6.7E-02	8.9E-01

* No. of genes down-regulated in Ovo-B KD PGCs.

Supplementary Table S4. The top 10 GO categories enriched in the genes up-regulated in Ovo-B KD PGCs

Biological Process

Term	Name	count*	p-value	q-value
GO:0048598	embryonic morphogenesis	26	9.8E-08	5.2E-05
GO:0048731	system development	83	7.6E-08	6.0E-05
GO:0009888	tissue development	38	1.5E-07	6.1E-05
GO:0048513	organ development	70	2.5E-07	8.1E-05
GO:0050896	response to stimulus	73	5.5E-08	8.8E-05
GO:0007444	imaginal disc development	35	6.4E-07	1.7E-04
GO:0009887	organ morphogenesis	47	1.4E-06	2.8E-04
GO:0007610	behavior	35	1.6E-06	2.9E-04
GO:0007166	cell surface receptor linked signal transduction	45	1.3E-06	2.9E-04
GO:0009790	embryonic development	42	2.7E-06	4.0E-04

Molecular Function

Term	Name	count*	p-value	q-value
GO:0004364	glutathione transferase activity	12	3.3E-08	1.6E-05
GO:0008083	growth factor activity	9	6.4E-06	1.0E-03
GO:0016765	transferase activity, transferring alkyl or aryl (other than methyl) groups	12	6.3E-06	1.5E-03
GO:0005102	receptor binding	19	1.8E-05	2.1E-03
GO:0004872	receptor activity	36	3.6E-05	3.5E-03
GO:0004871	signal transducer activity	42	5.7E-05	4.6E-03
GO:0060089	molecular transducer activity	42	5.7E-05	4.6E-03
GO:0003779	actin binding	13	1.2E-03	7.9E-02
GO:0004888	transmembrane receptor activity	26	2.1E-03	1.2E-01
GO:0015020	glucuronosyltransferase activity	6	6.3E-03	2.6E-01

* No. of genes up-regulated in Ovo-B KD PGCs.

Supplementary Table S5. List of soma-enriched genes up-regulated in Ovo-B KD PGCs

* This table shows the names of the genes expressed predominantly in PGCs (Adjusted p-value < 0.05; see SI Materials and Methods) that were significantly up-regulated in Ovo-B KD PGCs in comparison with control PGCs (q-value < 0.05; see SI Materials and Methods).

† The cell-type effect (C_i) value was calculated using a linear model (see SI Materials and Methods). C_i represents the estimated fold change in PGCs relative to whole embryos (soma) in $\log_2$ scale.

‡ The genotype effect (G_i) value, or the estimated fold change in control PGCs vs Ovo-A–expressing PGCs in $\log_2$ scale (Table S1) is shown again.

	PGCs/soma				Cont./OvoB KD				
Gene Name*	Probe Name	Cell-type effect† [Fold Change; $\log_2$ (PGCs/soma)]	p-value	Adjusted p-value	Feature Number	Probe Name	Genotype effect ‡ [Fold Change; $\log_2$ (Cont./OvoB KD)]	p-value	q-value
abd-A	CG10325_ESCL1_4595	-4.122	3.09.E-51	6.92.E-47	12788	A_09_P041071	-4.653	3.53.E-05	7.50.E-03
					22071	A_09_P041071	-4.302	3.85.E-05	7.50.E-03
					5347	A_09_P052391	-1.528	9.11.E-05	9.01.E-03
abd-A	CG10325_ESCL2_926	-1.024	1.26.E-15	2.15.E-11	12788	A_09_P041071	-4.653	3.53.E-05	7.50.E-03
					22071	A_09_P041071	-4.302	3.85.E-05	7.50.E-03
					5347	A_09_P052391	-1.528	9.11.E-05	9.01.E-03
Abd-B	CG11648_ESCL1_3370	-2.553	6.05.E-34	1.28.E-29	40218	A_09_P052351	-2.653	3.58.E-04	1.47.E-02
					20805	A_09_P052346	-2.598	2.09.E-05	6.43.E-03
					26764	A_09_P041076	-1.917	3.93.E-04	1.54.E-02
					29190	A_09_P041076	-1.800	3.60.E-04	1.47.E-02
					35341	A_09_P051891	-1.427	9.80.E-04	2.40.E-02
Abd-B	CG11648_ESCL2_623	-1.591	1.05.E-26	2.09.E-22	40218	A_09_P052351	-2.653	3.58.E-04	1.47.E-02
					20805	A_09_P052346	-2.598	2.09.E-05	6.43.E-03
					26764	A_09_P041076	-1.917	3.93.E-04	1.54.E-02
					29190	A_09_P041076	-1.800	3.60.E-04	1.47.E-02
					35341	A_09_P051891	-1.427	9.80.E-04	2.40.E-02
Abd-B	CGY0179_ESCL1_610	-2.239	7.69.E-18	1.36.E-13	40218	A_09_P052351	-2.653	3.58.E-04	1.47.E-02
					20805	A_09_P052346	-2.598	2.09.E-05	6.43.E-03
					26764	A_09_P041076	-1.917	3.93.E-04	1.54.E-02
					29190	A_09_P041076	-1.800	3.60.E-04	1.47.E-02
					35341	A_09_P051891	-1.427	9.80.E-04	2.40.E-02
Ac78C	CG10564_ESCL1_6216	-2.805	3.76.E-32	7.85.E-28	21915	A_09_P076641	-2.161	4.16.E-05	7.50.E-03
					33688	A_09_P076641	-2.007	1.75.E-04	1.14.E-02
Ac78C	CG10564_ESCL2_5022	-2.284	5.20.E-32	1.09.E-27	21915	A_09_P076641	-2.161	4.16.E-05	7.50.E-03
					33688	A_09_P076641	-2.007	1.75.E-04	1.14.E-02
Aldh	CG3752_ESCL1_2113	-0.880	8.33.E-13	1.33.E-08	20565	A_09_P029496	-1.094	7.83.E-04	2.14.E-02
					29958	A_09_P029496	-1.035	8.95.E-04	2.30.E-02
Aldh	CG3752_ESCL2_1497	-0.600	5.92.E-13	9.51.E-09	20565	A_09_P029496	-1.094	7.83.E-04	2.14.E-02
					29958	A_09_P029496	-1.035	8.95.E-04	2.30.E-02
AnnIX	CG5730_ESCL1_1198	-0.958	6.08.E-16	1.04.E-11	38045	A_09_P166180	-0.936	3.01.E-03	4.03.E-02
AnnIX	CG5730_ESCL2_897	-1.182	4.78.E-27	9.52.E-23	38045	A_09_P166180	-0.936	3.01.E-03	4.03.E-02
AnnIX	CG5730_ESCL3_850	-1.550	4.44.E-30	9.11.E-26	38045	A_09_P166180	-0.936	3.01.E-03	4.03.E-02
AnnIX	CGY0327_ESCL1_712	-2.026	7.45.E-43	1.64.E-38	38045	A_09_P166180	-0.936	3.01.E-03	4.03.E-02
aop	CG3166_ESCL1_4748	-2.525	2.14.E-42	4.71.E-38	2136	A_09_P041291	-1.243	7.98.E-04	2.17.E-02
					32150	A_09_P041291	-1.232	1.03.E-03	2.46.E-02
aop	CG3166_ESCL2_1977	-1.499	7.64.E-32	1.59.E-27	2136	A_09_P041291	-1.243	7.98.E-04	2.17.E-02
					32150	A_09_P041291	-1.232	1.03.E-03	2.46.E-02
Arc1	CG12505_ESCL1_2260	-1.871	6.12.E-30	1.25.E-25	16628	A_09_P007636	-0.981	2.05.E-03	3.46.E-02
					14034	A_09_P007636	-0.861	4.49.E-03	4.89.E-02
ASPP	CG18375_ESCL1_4512	-1.404	3.46.E-26	6.82.E-22	988	A_09_P026031	-1.481	5.76.E-04	1.85.E-02
					23764	A_09_P026031	-1.383	4.48.E-04	1.63.E-02
ASPP	CG18375_ESCL2_2727	-0.555	3.30.E-07	4.41.E-03	988	A_09_P026031	-1.481	5.76.E-04	1.85.E-02
					23764	A_09_P026031	-1.383	4.48.E-04	1.63.E-02
bbg	CG9598_ESCL1_3583	-1.696	2.00.E-24	3.87.E-20	13649	A_09_P048181	-0.776	3.38.E-03	4.26.E-02
bbg	CG9598_ESCL2_3206	-1.426	1.21.E-26	2.40.E-22	13649	A_09_P048181	-0.776	3.38.E-03	4.26.E-02
betaTub60D	CG3401_ESCL1_2368	-4.630	1.17.E-45	2.61.E-41	38436	A_09_P009901	-1.363	3.33.E-04	1.45.E-02
					25740	A_09_P009901	-1.223	1.25.E-03	2.71.E-02
betaTub60D	CG3401_ESCL2_1303	-2.449	2.06.E-22	3.89.E-18	38436	A_09_P009901	-1.363	3.33.E-04	1.45.E-02
					25740	A_09_P009901	-1.223	1.25.E-03	2.71.E-02
bgm	CG4501_ESCL1_2334	-1.838	1.98.E-38	4.29.E-34	14579	A_09_P079256	-0.879	2.99.E-03	4.02.E-02
					4531	A_09_P079256	-0.860	3.30.E-03	4.22.E-02
					5272	A_09_P110773	-0.821	3.38.E-03	4.26.E-02

bgm	CG4501_ESCL2_1898	-0.467	2.05.E-06	2.64.E-02	14579	A_09_P079256	-0.879	2.99.E-03	4.02.E-02
					4531	A_09_P079256	-0.860	3.30.E-03	4.22.E-02
					5272	A_09_P110773	-0.821	3.38.E-03	4.26.E-02
blot	CG3897_ESCL1_3527	-3.854	1.12.E-45	2.48.E-41	3575	A_09_P079886	-2.440	2.34.E-03	3.64.E-02
					24204	A_09_P079886	-2.400	1.04.E-03	2.47.E-02
blot	CG3897_ESCL2_2857	-1.878	1.56.E-38	3.40.E-34	3575	A_09_P079886	-2.440	2.34.E-03	3.64.E-02
					24204	A_09_P079886	-2.400	1.04.E-03	2.47.E-02
bnb	CG7088_ESCL1_1954	-4.451	5.30.E-44	1.17.E-39	3533	A_09_P042516	-1.057	7.06.E-04	2.03.E-02
					26570	A_09_P042516	-0.997	1.17.E-03	2.63.E-02
bnb	CG7088_ESCL2_1255	-3.465	1.89.E-37	4.09.E-33	3533	A_09_P042516	-1.057	7.06.E-04	2.03.E-02
					26570	A_09_P042516	-0.997	1.17.E-03	2.63.E-02
bowl	CG10021_ESCL1_3301	-4.040	2.04.E-42	4.50.E-38	11503	A_09_P011276	-1.470	8.29.E-04	2.21.E-02
					33128	A_09_P011276	-1.228	3.13.E-03	4.11.E-02
bowl	CG10021_ESCL2_2071	-1.568	7.77.E-16	1.33.E-11	11503	A_09_P011276	-1.470	8.29.E-04	2.21.E-02
					33128	A_09_P011276	-1.228	3.13.E-03	4.11.E-02
Brd	CG3096_ESCL1_381	-4.229	3.59.E-52	8.06.E-48	7429	A_09_P041496	-1.579	6.38.E-04	1.94.E-02
					45209	A_09_P041496	-1.526	4.21.E-04	1.58.E-02
Brd	CG3096_ESCL2_181	-4.295	2.09.E-37	4.51.E-33	7429	A_09_P041496	-1.579	6.38.E-04	1.94.E-02
					45209	A_09_P041496	-1.526	4.21.E-04	1.58.E-02
bru-2	CG31761_ESCL1_3516	-3.249	2.47.E-24	4.77.E-20	21208	A_09_P056661	-1.343	3.48.E-03	4.31.E-02
					4274	A_09_P056661	-1.241	3.14.E-03	4.11.E-02
					20031	A_09_P226000	-0.848	2.78.E-03	3.91.E-02
bru-2	CG31761_ESCL3_2123	-1.062	6.40.E-13	1.03.E-08	21208	A_09_P056661	-1.343	3.48.E-03	4.31.E-02
					4274	A_09_P056661	-1.241	3.14.E-03	4.11.E-02
					20031	A_09_P226000	-0.848	2.78.E-03	3.91.E-02
Calx	CG5685_ESCL3_3643	-2.128	1.68.E-19	3.05.E-15	38079	A_09_P051221	-1.208	3.18.E-03	4.15.E-02
CBP	CG1435_ESCL1_1982	-3.370	2.17.E-50	4.85.E-46	10636	A_09_P106320	-1.386	1.61.E-04	1.08.E-02
CBP	CG1435_ESCL2_820	-2.205	2.34.E-43	5.17.E-39	10636	A_09_P106320	-1.386	1.61.E-04	1.08.E-02
CG10082	CG10082_ESCL1_3396	-1.162	5.53.E-30	1.13.E-25	17141	A_09_P109995	-1.201	1.71.E-03	3.20.E-02
					16771	A_09_P026136	-1.098	1.81.E-03	3.24.E-02
CG10082	CG10082_ESCL2_2614	-0.550	1.45.E-10	2.18.E-06	17141	A_09_P109995	-1.201	1.71.E-03	3.20.E-02
					16771	A_09_P026136	-1.098	1.81.E-03	3.24.E-02
CG10176	CG10176_ESCL1_1652	-1.519	7.50.E-23	1.42.E-18	2508	A_09_P023131	-1.008	3.70.E-03	4.46.E-02
CG10176	CG10176_ESCL2_644	-2.293	9.93.E-28	1.99.E-23	2508	A_09_P023131	-1.008	3.70.E-03	4.46.E-02
CG11033	CG11033_ESCL1_5185	-0.775	3.46.E-21	6.44.E-17	37635	A_09_P072866	-1.291	2.66.E-04	1.31.E-02
					36960	A_09_P072866	-1.278	2.52.E-04	1.29.E-02
CG1124	CG1124_ESCL2_620	-0.895	2.52.E-11	3.88.E-07	19971	A_09_P036191	-1.453	6.24.E-04	1.92.E-02
CG11275	CG11275_ESCL1_1180	-1.937	1.65.E-18	2.95.E-14	16210	A_09_P026356	-1.720	1.32.E-04	1.02.E-02
					26498	A_09_P026356	-1.585	1.54.E-04	1.07.E-02
CG11347	CG11347_ESCL1_1994	-2.710	3.57.E-36	7.67.E-32	34645	A_09_P052686	-3.382	3.36.E-06	5.81.E-03
					43192	A_09_P052686	-3.238	7.97.E-06	5.81.E-03
CG11347	CG11347_ESCL2_984	-2.506	2.52.E-33	5.31.E-29	34645	A_09_P052686	-3.382	3.36.E-06	5.81.E-03
					43192	A_09_P052686	-3.238	7.97.E-06	5.81.E-03
CG11825	CG11825_ESCL1_437	-0.519	1.88.E-08	2.64.E-04	43977	A_09_P107930	-1.489	2.30.E-04	1.25.E-02
					25124	A_09_P006106	-1.384	1.81.E-04	1.14.E-02
					9572	A_09_P176590	-1.182	2.55.E-03	3.77.E-02
CG11825	CG11825_ESCL2_45	-0.442	3.03.E-08	4.23.E-04	43977	A_09_P107930	-1.489	2.30.E-04	1.25.E-02
					25124	A_09_P006106	-1.384	1.81.E-04	1.14.E-02
					9572	A_09_P176590	-1.182	2.55.E-03	3.77.E-02
CG12171	CG12171_ESCL1_939	-1.456	1.81.E-24	3.51.E-20	36148	A_09_P036451	-1.271	2.15.E-03	3.54.E-02
					35902	A_09_P036451	-1.244	1.81.E-03	3.24.E-02
CG12171	CG12171_ESCL2_449	-0.798	5.21.E-18	9.25.E-14	36148	A_09_P036451	-1.271	2.15.E-03	3.54.E-02
					35902	A_09_P036451	-1.244	1.81.E-03	3.24.E-02
CG12262	CG12262_ESCL1_1183	-1.645	2.34.E-43	5.18.E-39	39508	A_09_P053671	-1.010	9.72.E-04	2.40.E-02
					21243	A_09_P053671	-0.949	1.35.E-03	2.82.E-02
CG12420	CG12420_ESCL1_419	-2.090	2.20.E-33	4.64.E-29	42252	A_09_P073411	-1.631	2.53.E-04	1.29.E-02
					4402	A_09_P073411	-1.115	1.72.E-03	3.20.E-02
					27977	A_09_P217695	-0.857	2.66.E-03	3.83.E-02
CG12512	CG12512_ESCL1_1685	-0.885	2.08.E-17	3.66.E-13	4762	A_09_P019566	-1.572	2.43.E-04	1.26.E-02
					24972	A_09_P019566	-1.028	4.56.E-03	4.94.E-02
CG12581	CG12581_ESCL1_2963	-3.767	2.19.E-55	4.92.E-51	6222	A_09_P035926	-1.503	3.26.E-03	4.20.E-02
CG12581	CG12581_ESCL2_1837	-2.534	7.52.E-40	1.64.E-35	6222	A_09_P035926	-1.503	3.26.E-03	4.20.E-02
CG12730	CG12730_ESCL2_357	-0.865	2.48.E-12	3.92.E-08	12559	A_09_P067256	-1.531	1.80.E-04	1.14.E-02
					31911	A_09_P067256	-1.511	6.62.E-04	1.97.E-02
					10927	A_09_P067256	-1.503	9.48.E-04	2.36.E-02
					26014	A_09_P067256	-1.499	1.10.E-03	2.55.E-02
					21173	A_09_P067256	-1.485	3.93.E-04	1.54.E-02
					9464	A_09_P067256	-1.451	6.83.E-04	2.01.E-02
					41888	A_09_P067256	-1.340	8.56.E-04	2.25.E-02

					25527	A_09_P067256	-1.330	6.61.E-04	1.97.E-02
					34019	A_09_P067256	-1.304	1.02.E-03	2.45.E-02
					5280	A_09_P067256	-1.228	9.76.E-04	2.40.E-02
CG12768	CG12768_ESCL2_1200	-0.988	3.38.E-16	5.81.E-12	37853	A_09_P035906	-2.684	7.21.E-05	8.48.E-03
					31874	A_09_P035906	-2.269	1.29.E-04	1.01.E-02
					17076	A_09_P127480	-2.247	1.30.E-04	1.01.E-02
CG12814	CG12814_ESCL1_440	-1.022	9.68.E-13	1.55.E-08	36279	A_09_P073406	-1.852	3.12.E-03	4.11.E-02
					20668	A_09_P217690	-0.890	1.87.E-03	3.30.E-02
CG12814	CG12814_ESCL2_6590	-0.973	2.47.E-09	3.57.E-05	36279	A_09_P073406	-1.852	3.12.E-03	4.11.E-02
					20668	A_09_P217690	-0.890	1.87.E-03	3.30.E-02
CG12896	CG12896_ESCL1_561	-2.842	3.76.E-45	8.34.E-41	13280	A_09_P006111	-1.381	5.17.E-04	1.75.E-02
CG13004	CG13004_ESCL1_866	-0.592	3.48.E-12	5.48.E-08	11377	A_09_P039601	-1.910	6.99.E-04	2.02.E-02
					27472	A_09_P193025	-1.599	2.13.E-03	3.51.E-02
CG13252	CG13252_ESCL1_2388	-1.550	7.14.E-26	1.40.E-21	35449	A_09_P035206	-2.541	4.21.E-03	4.73.E-02
					27084	A_09_P035206	-1.869	4.09.E-04	1.57.E-02
CG13506	CG13506_ESCL1_2781	-3.462	1.04.E-26	2.07.E-22	25461	A_09_P026436	-0.733	4.02.E-03	4.63.E-02
CG13868	CG13868_ESCL1_3771	-1.096	1.69.E-11	2.61.E-07	10349	A_09_P025661	-3.214	2.29.E-05	6.66.E-03
					4276	A_09_P025661	-2.973	1.18.E-05	5.94.E-03
CG13868	CG13868_ESCL2_989	-1.212	4.34.E-09	6.24.E-05	10349	A_09_P025661	-3.214	2.29.E-05	6.66.E-03
					4276	A_09_P025661	-2.973	1.18.E-05	5.94.E-03
CG13868	CG13868_ESCL3_2697	-1.591	4.73.E-24	9.13.E-20	10349	A_09_P025661	-3.214	2.29.E-05	6.66.E-03
					4276	A_09_P025661	-2.973	1.18.E-05	5.94.E-03
CG13928	CG13928_ESCL1_2446	-1.206	9.20.E-23	1.75.E-18	34987	A_09_P028391	-1.388	1.11.E-03	2.56.E-02
CG14253	CG14253_ESCL1_1747	-1.972	1.11.E-21	2.08.E-17	32595	A_09_P185065	-0.813	2.87.E-03	3.97.E-02
CG14253	CG14253_ESCL2_970	-1.003	2.08.E-10	3.12.E-06	32595	A_09_P185065	-0.813	2.87.E-03	3.97.E-02
CG14915	CG14915_ESCL1_269	-4.216	5.20.E-55	1.17.E-50	14177	A_09_P217065	-1.324	8.24.E-04	2.20.E-02
					23204	A_09_P021896	-1.221	9.92.E-04	2.42.E-02
					14166	A_09_P021896	-1.097	6.04.E-04	1.90.E-02
CG14937	CG14937_ESCL1_1377	-1.025	4.64.E-21	8.63.E-17	28453	A_09_P022071	-1.371	5.00.E-04	1.71.E-02
					21725	A_09_P022071	-1.221	7.18.E-04	2.05.E-02
CG14937	CG14937_ESCL2_1269	-1.476	1.43.E-23	2.74.E-19	28453	A_09_P022071	-1.371	5.00.E-04	1.71.E-02
					21725	A_09_P022071	-1.221	7.18.E-04	2.05.E-02
CG14968	CG14968_ESCL1_847	-0.851	4.36.E-12	6.84.E-08	15551	A_09_P029061	-0.802	4.48.E-03	4.89.E-02
CG14968	CG14968_ESCL2_504	-0.388	3.38.E-06	4.32.E-02	15551	A_09_P029061	-0.802	4.48.E-03	4.89.E-02
CG15479	CG15479_ESCL1_548	-3.639	4.23.E-35	9.02.E-31	4850	A_09_P022531	-2.499	1.15.E-05	5.91.E-03
					19585	A_09_P022531	-2.468	2.98.E-05	7.10.E-03
					9107	A_09_P022531	-2.466	9.88.E-06	5.81.E-03
					43269	A_09_P022531	-2.449	3.19.E-04	1.44.E-02
					45086	A_09_P022531	-2.411	8.68.E-06	5.81.E-03
					20858	A_09_P022531	-2.366	1.08.E-04	9.43.E-03
					12087	A_09_P022531	-2.223	1.23.E-04	9.98.E-03
					36112	A_09_P022531	-2.114	4.01.E-05	7.50.E-03
					16017	A_09_P022531	-2.016	1.93.E-04	1.17.E-02
					33318	A_09_P022531	-1.997	8.61.E-05	8.82.E-03
CG15658	CG15658_ESCL1_1526	-1.721	5.29.E-26	1.04.E-21	10894	A_09_P026011	-1.107	1.52.E-03	3.00.E-02
					41168	A_09_P026011	-1.059	2.21.E-03	3.59.E-02
CG1600	CG1600_ESCL1_1833	-2.017	1.08.E-30	2.22.E-26	7214	A_09_P024801	-1.099	1.29.E-03	2.75.E-02
CG1600	CG1600_ESCL2_1079	-1.208	3.07.E-17	5.38.E-13	7214	A_09_P024801	-1.099	1.29.E-03	2.75.E-02
CG16791	CG16791_ESCL1_4632	-2.344	2.61.E-32	5.46.E-28	17496	A_09_P175930	-0.894	1.62.E-03	3.09.E-02
CG16791	CG16791_ESCL2_3193	-1.922	1.14.E-27	2.29.E-23	17496	A_09_P175930	-0.894	1.62.E-03	3.09.E-02
CG17181	CG17181_ESCL1_1242	-0.613	1.09.E-07	1.49.E-03	12550	A_09_P214545	-1.060	9.76.E-04	2.40.E-02
					31575	A_09_P028011	-1.052	4.14.E-03	4.69.E-02
CG17211	CG17211_ESCL1_3981	-1.229	2.20.E-20	4.06.E-16	11391	A_09_P022226	-2.032	3.81.E-03	4.49.E-02
					16398	A_09_P022226	-1.921	3.98.E-03	4.61.E-02
CG17278	CG17278_ESCL1_1815	-1.907	9.77.E-13	1.56.E-08	34660	A_09_P202905	-2.343	4.28.E-05	7.50.E-03
					10810	A_09_P057171	-1.481	5.79.E-04	1.85.E-02
CG17278	CG17278_ESCL2_178	-1.920	1.43.E-23	2.74.E-19	34660	A_09_P202905	-2.343	4.28.E-05	7.50.E-03
					10810	A_09_P057171	-1.481	5.79.E-04	1.85.E-02
CG17321	CG17321_ESCL1_1921	-2.610	1.23.E-13	2.00.E-09	13542	A_09_P023256	-0.817	2.60.E-03	3.80.E-02
CG17323	CG17323_ESCL2_1422	-0.449	1.31.E-08	1.85.E-04	38180	A_09_P023241	-1.282	2.80.E-03	3.93.E-02
CG17574	CG17574_ESCL1_1050	-1.547	2.66.E-31	5.51.E-27	17747	A_09_P007071	-0.839	2.27.E-03	3.60.E-02
CG17574	CG17574_ESCL2_407	-0.727	2.70.E-12	4.26.E-08	17747	A_09_P007071	-0.839	2.27.E-03	3.60.E-02
CG18542	CG18542_ESCL1_1132	-0.635	5.56.E-12	8.69.E-08	9518	A_09_P133175	-0.820	3.57.E-03	4.37.E-02
CG2016	CG2016_ESCL1_656	-2.557	1.51.E-29	3.07.E-25	25799	A_09_P187190	-1.458	3.26.E-03	4.20.E-02
CG3097	CG3097_ESCL1_1891	-1.089	4.23.E-18	7.53.E-14	42288	A_09_P067346	-1.161	7.96.E-04	2.16.E-02
CG3097	CG3097_ESCL2_1181	-2.844	1.87.E-41	4.11.E-37	42288	A_09_P067346	-1.161	7.96.E-04	2.16.E-02
CG31140	CG31140_ESCL1_6642	-0.946	2.66.E-09	3.86.E-05	18699	A_09_P204740	-1.424	2.83.E-03	3.95.E-02
CG31300	CG31300_ESCL1_1187	-2.284	2.98.E-27	5.94.E-23	11284	A_09_P059556	-1.192	2.48.E-03	3.72.E-02
CG31300	CG31300_ESCL2_1294	-1.988	6.69.E-15	1.12.E-10	11284	A_09_P059556	-1.192	2.48.E-03	3.72.E-02

CG31522	CG31522_ESCL1_2591	-2.184	7.88.E-35	1.68.E-30	40582	A_09_P109805	-1.193	4.77.E-04	1.68.E-02
CG32264	CG32264_ESCL1_3701	-0.888	4.20.E-13	6.77.E-09	20465	A_09_P185580	-1.157	1.57.E-03	3.04.E-02
					33561	A_09_P193010	-0.852	2.45.E-03	3.69.E-02
CG32264	CG32264_ESCL2_2741	-0.989	8.26.E-18	1.46.E-13	20465	A_09_P185580	-1.157	1.57.E-03	3.04.E-02
					33561	A_09_P193010	-0.852	2.45.E-03	3.69.E-02
CG32638	CG32638_ESCL1_1194	-2.139	4.96.E-33	1.04.E-28	25989	A_09_P016031	-1.114	4.73.E-04	1.67.E-02
					20854	A_09_P016031	-0.932	1.34.E-03	2.82.E-02
CG32638	CG32638_ESCL2_210	-0.944	6.27.E-19	1.13.E-14	25989	A_09_P016031	-1.114	4.73.E-04	1.67.E-02
					20854	A_09_P016031	-0.932	1.34.E-03	2.82.E-02
CG34383	CG14365_ESCL1_4382	-0.797	6.25.E-08	8.62.E-04	36656	A_09_P206085	-1.450	3.48.E-04	1.46.E-02
					11257	A_09_P171405	-1.235	7.54.E-04	2.10.E-02
					30729	A_09_P129800	-0.950	1.44.E-03	2.91.E-02
					18761	A_09_P004086	-0.925	1.20.E-03	2.66.E-02
CG34383	CG9322_ESCL1_2921	-2.759	5.44.E-35	1.16.E-30	36656	A_09_P206085	-1.450	3.48.E-04	1.46.E-02
					11257	A_09_P171405	-1.235	7.54.E-04	2.10.E-02
					30729	A_09_P129800	-0.950	1.44.E-03	2.91.E-02
					18761	A_09_P004086	-0.925	1.20.E-03	2.66.E-02
CG34383	CG9322_ESCL2_2392	-2.300	2.56.E-34	5.42.E-30	36656	A_09_P206085	-1.450	3.48.E-04	1.46.E-02
					11257	A_09_P171405	-1.235	7.54.E-04	2.10.E-02
					30729	A_09_P129800	-0.950	1.44.E-03	2.91.E-02
					18761	A_09_P004086	-0.925	1.20.E-03	2.66.E-02
CG3624	CG3624_ESCL1_978	-1.541	1.21.E-17	2.14.E-13	33714	A_09_P026441	-1.313	8.98.E-04	2.30.E-02
					18220	A_09_P026441	-1.137	2.38.E-03	3.66.E-02
					43123	A_09_P206660	-0.796	3.44.E-03	4.29.E-02
CG3624	CG3624_ESCL2_323	-1.314	1.36.E-15	2.31.E-11	33714	A_09_P026441	-1.313	8.98.E-04	2.30.E-02
					18220	A_09_P026441	-1.137	2.38.E-03	3.66.E-02
					43123	A_09_P206660	-0.796	3.44.E-03	4.29.E-02
CG3835	CG3835_ESCL1_1522	-0.449	2.09.E-06	2.69.E-02	27514	A_09_P076456	-3.092	1.24.E-05	6.01.E-03
					37933	A_09_P076456	-3.003	4.92.E-06	5.81.E-03
CG3842	CG3842_ESCL1_1157	-1.727	8.04.E-25	1.56.E-20	30715	A_09_P067601	-0.875	2.55.E-03	3.77.E-02
CG4115	CG4115_ESCL1_958	-2.095	5.19.E-33	1.09.E-28	9645	A_09_P074276	-1.513	6.47.E-04	1.96.E-02
CG4115	CG4115_ESCL2_562	-2.914	1.30.E-31	2.70.E-27	9645	A_09_P074276	-1.513	6.47.E-04	1.96.E-02
CG4164	CG4164_ESCL1_1406	-1.386	3.11.E-22	5.87.E-18	34306	A_09_P017961	-0.872	3.73.E-03	4.46.E-02
CG4164	CG4164_ESCL2_967	-0.952	3.00.E-35	6.40.E-31	34306	A_09_P017961	-0.872	3.73.E-03	4.46.E-02
CG42358	CG5558_ESCL1_1178	-0.303	7.52.E-07	9.90.E-03	17005	A_09_P069126	-1.866	4.12.E-05	7.50.E-03
					20366	A_09_P069126	-1.785	3.69.E-05	7.50.E-03
CG42663	CG12488_ESCL2_1441	-1.207	1.74.E-14	2.88.E-10	904	A_09_P209265	-1.165	1.24.E-03	2.70.E-02
CG43340	CG30492_ESCL1_3837	-0.727	1.61.E-08	2.27.E-04	22520	A_09_P079786	-0.995	1.60.E-03	3.07.E-02
					32131	A_09_P079786	-0.933	1.93.E-03	3.36.E-02
CG43340	CG30492_ESCL2_3741	-1.460	7.46.E-31	1.54.E-26	22520	A_09_P079786	-0.995	1.60.E-03	3.07.E-02
					32131	A_09_P079786	-0.933	1.93.E-03	3.36.E-02
CG43340	CG30492_ESCL4_2753	-1.293	3.51.E-25	6.85.E-21	22520	A_09_P079786	-0.995	1.60.E-03	3.07.E-02
					32131	A_09_P079786	-0.933	1.93.E-03	3.36.E-02
CG4455	CG4455_ESCL1_1333	-2.883	2.61.E-53	5.86.E-49	41762	A_09_P065241	-1.100	2.60.E-03	3.80.E-02
CG4455	CG4455_ESCL2_315	-0.354	1.74.E-06	2.25.E-02	41762	A_09_P065241	-1.100	2.60.E-03	3.80.E-02
CG4688	CG4688_ESCL1_934	-1.391	8.42.E-13	1.35.E-08	22304	A_09_P151205	-1.418	1.58.E-03	3.05.E-02
CG4688	CG4688_ESCL2_398	-1.063	2.61.E-12	4.12.E-08	22304	A_09_P151205	-1.418	1.58.E-03	3.05.E-02
CG5059	CG5059_ESCL1_1639	-0.943	1.96.E-17	3.46.E-13	38442	A_09_P035161	-1.196	5.00.E-04	1.71.E-02
CG5059	CG5059_ESCL2_541	-0.870	1.39.E-18	2.50.E-14	38442	A_09_P035161	-1.196	5.00.E-04	1.71.E-02
CG5455	CG5455_ESCL1_2739	-2.684	4.47.E-32	9.33.E-28	8374	A_09_P071986	-1.106	1.75.E-03	3.20.E-02
					7792	A_09_P071986	-1.057	1.37.E-03	2.83.E-02
CG5455	CG5455_ESCL2_1993	-1.882	1.17.E-20	2.16.E-16	8374	A_09_P071986	-1.106	1.75.E-03	3.20.E-02
					7792	A_09_P071986	-1.057	1.37.E-03	2.83.E-02
CG5707	CG5707_ESCL1_1393	-3.127	1.07.E-33	2.25.E-29	19040	A_09_P078761	-1.098	3.78.E-03	4.48.E-02
					17610	A_09_P078761	-1.029	3.28.E-03	4.20.E-02
CG5707	CG5707_ESCL2_981	-0.498	7.82.E-07	1.03.E-02	19040	A_09_P078761	-1.098	3.78.E-03	4.48.E-02
					17610	A_09_P078761	-1.029	3.28.E-03	4.20.E-02
CG5835	CG11779_ESCL3_2331	-0.977	3.71.E-12	5.84.E-08	35390	A_09_P069116	-1.141	5.70.E-04	1.84.E-02
					23378	A_09_P069106	-1.128	4.32.E-04	1.61.E-02
CG5835	CG5835_ESCL1_1255	-0.843	1.74.E-11	2.69.E-07	35390	A_09_P069116	-1.141	5.70.E-04	1.84.E-02
					23378	A_09_P069106	-1.128	4.32.E-04	1.61.E-02
CG5869	CG5869_ESCL1_809	-1.461	3.15.E-25	6.16.E-21	13602	A_09_P065986	-0.775	3.73.E-03	4.46.E-02
					6974	A_09_P065986	-0.757	3.96.E-03	4.59.E-02
CG5869	CG5869_ESCL2_240	-1.137	3.37.E-25	6.57.E-21	13602	A_09_P065986	-0.775	3.73.E-03	4.46.E-02
					6974	A_09_P065986	-0.757	3.96.E-03	4.59.E-02
CG6024	CG6024_ESCL1_4020	-0.619	2.01.E-07	2.71.E-03	781	A_09_P055081	-2.308	2.97.E-04	1.37.E-02
CG6045	CG6045_ESCL1_3692	-0.555	1.29.E-09	1.88.E-05	6034	A_09_P075516	-1.226	1.74.E-03	3.20.E-02
					21393	A_09_P075516	-1.154	1.54.E-03	3.02.E-02
CG6206	CG6206_ESCL1_3146	-1.484	2.28.E-36	4.89.E-32	37642	A_09_P079826	-1.519	1.02.E-03	2.45.E-02

CG6231	CG6231_ESCL1_2874	-0.433	1.07.E-07	1.46.E-03	9422	A_09_P148150	-1.333	1.51.E-03	3.00.E-02
CG6231	CG6231_ESCL2_1692	-1.250	4.24.E-20	7.78.E-16	9422	A_09_P148150	-1.333	1.51.E-03	3.00.E-02
CG6329	CG6329_ESCL1_2566	-1.183	1.67.E-10	2.52.E-06	37001	A_09_P007421	-0.920	2.25.E-03	3.60.E-02
					6315	A_09_P007421	-0.795	4.48.E-03	4.89.E-02
CG6329	CG6329_ESCL2_974	-0.937	6.72.E-11	1.02.E-06	37001	A_09_P007421	-0.920	2.25.E-03	3.60.E-02
					6315	A_09_P007421	-0.795	4.48.E-03	4.89.E-02
CG6329	CG6329_ESCL3_261	-0.829	1.73.E-17	3.05.E-13	37001	A_09_P007421	-0.920	2.25.E-03	3.60.E-02
					6315	A_09_P007421	-0.795	4.48.E-03	4.89.E-02
CG6330	CG6330_ESCL1_1581	-3.251	3.11.E-53	6.99.E-49	2632	A_09_P072126	-2.942	1.87.E-04	1.16.E-02
					19820	A_09_P072126	-2.828	1.68.E-04	1.11.E-02
CG6330	CG6330_ESCL2_902	-1.870	1.08.E-37	2.33.E-33	2632	A_09_P072126	-2.942	1.87.E-04	1.16.E-02
					19820	A_09_P072126	-2.828	1.68.E-04	1.11.E-02
CG6356	CG6356_ESCL1_1602	-0.947	4.44.E-17	7.76.E-13	8838	A_09_P071031	-1.208	3.27.E-04	1.45.E-02
					43638	A_09_P071031	-0.977	1.32.E-03	2.79.E-02
CG6356	CG6356_ESCL2_2471	-1.865	7.33.E-13	1.18.E-08	8838	A_09_P071031	-1.208	3.27.E-04	1.45.E-02
					43638	A_09_P071031	-0.977	1.32.E-03	2.79.E-02
CG6520	CG6520_ESCL1_759	-4.121	1.22.E-45	2.71.E-41	6972	A_09_P008696	-1.189	3.41.E-03	4.28.E-02
					11212	A_09_P008696	-0.740	3.93.E-03	4.58.E-02
CG6520	CG6520_ESCL2_459	-3.179	3.90.E-42	8.59.E-38	6972	A_09_P008696	-1.189	3.41.E-03	4.28.E-02
					11212	A_09_P008696	-0.740	3.93.E-03	4.58.E-02
CG7407	CG7407_ESCL1_863	-0.596	2.73.E-11	4.20.E-07	4134	A_09_P035646	-0.867	2.49.E-03	3.73.E-02
CG7407	CG7407_ESCL2_427	-0.380	1.85.E-06	2.39.E-02	4134	A_09_P035646	-0.867	2.49.E-03	3.73.E-02
CG7560	CG7560_ESCL1_961	-0.627	2.93.E-09	4.23.E-05	10775	A_09_P054921	-1.239	4.98.E-04	1.71.E-02
CG7884	CG7884_ESCL1_3848	-4.317	3.82.E-40	8.35.E-36	5576	A_09_P040416	-1.962	6.94.E-04	2.02.E-02
					12776	A_09_P040416	-1.942	2.91.E-04	1.36.E-02
CG8398	CG8398_ESCL1_1865	-2.907	5.33.E-25	1.04.E-20	28214	A_09_P053301	-1.028	3.20.E-03	4.16.E-02
CG8398	CG8398_ESCL2_1545	-1.293	2.33.E-18	4.16.E-14	28214	A_09_P053301	-1.028	3.20.E-03	4.16.E-02
CG8420	CG8420_ESCL1_1495	-3.090	1.60.E-46	3.57.E-42	10223	A_09_P072891	-2.847	2.45.E-05	6.83.E-03
					44215	A_09_P072891	-2.835	3.85.E-05	7.50.E-03
CG8420	CG8420_ESCL2_1845	-3.743	1.74.E-53	3.90.E-49	10223	A_09_P072891	-2.847	2.45.E-05	6.83.E-03
					44215	A_09_P072891	-2.835	3.85.E-05	7.50.E-03
CG9095	CG9095_ESCL1_4654	-2.303	1.58.E-27	3.16.E-23	1736	A_09_P038876	-1.409	2.74.E-04	1.33.E-02
					37056	A_09_P038876	-0.998	3.43.E-03	4.28.E-02
CG9095	CG9095_ESCL2_2693	-1.270	3.97.E-13	6.41.E-09	1736	A_09_P038876	-1.409	2.74.E-04	1.33.E-02
					37056	A_09_P038876	-0.998	3.43.E-03	4.28.E-02
CG9232	CG9232_ESCL1_981	-2.840	4.07.E-43	9.00.E-39	29737	A_09_P020086	-2.051	7.75.E-04	2.13.E-02
CG9297	CG9297_ESCL1_3914	-0.600	1.85.E-07	2.50.E-03	41692	A_09_P194255	-1.369	3.47.E-04	1.46.E-02
CG9449	CG9449_ESCL2_1138	-3.236	1.59.E-39	3.46.E-35	36111	A_09_P034606	-2.175	4.27.E-03	4.76.E-02
					34075	A_09_P034606	-1.910	1.77.E-03	3.21.E-02
CG9449	CG9449_ESCL3_1297	-2.824	6.49.E-37	1.40.E-32	36111	A_09_P034606	-2.175	4.27.E-03	4.76.E-02
					34075	A_09_P034606	-1.910	1.77.E-03	3.21.E-02
CG9894	CG9894_ESCL1_796	-3.341	1.55.E-29	3.16.E-25	43374	A_09_P221985	-1.196	8.47.E-04	2.23.E-02
					30216	A_09_P174270	-1.071	2.57.E-03	3.78.E-02
CG9894	CG9894_ESCL2_314	-3.530	9.35.E-52	2.10.E-47	43374	A_09_P221985	-1.196	8.47.E-04	2.23.E-02
					30216	A_09_P174270	-1.071	2.57.E-03	3.78.E-02
cib	CG4944_ESCL3_312	-0.321	1.75.E-06	2.26.E-02	43888	A_09_P078106	-1.082	7.76.E-04	2.13.E-02
					28795	A_09_P078106	-1.038	1.21.E-03	2.66.E-02
					30843	A_09_P078106	-1.024	1.31.E-03	2.77.E-02
					13015	A_09_P078106	-1.013	1.17.E-03	2.64.E-02
					4534	A_09_P078106	-0.991	1.45.E-03	2.92.E-02
					16279	A_09_P078106	-0.989	1.54.E-03	3.02.E-02
					28585	A_09_P078106	-0.985	1.20.E-03	2.66.E-02
					30477	A_09_P078106	-0.980	1.26.E-03	2.71.E-02
					23224	A_09_P078106	-0.974	1.37.E-03	2.83.E-02
					17034	A_09_P078106	-0.914	1.57.E-03	3.04.E-02
cora	CG11949_ESCL1_2820	-4.114	4.42.E-48	9.87.E-44	38106	A_09_P012506	-0.914	2.60.E-03	3.80.E-02
cora	CG11949_ESCL2_2011	-3.520	8.79.E-55	1.98.E-50	38106	A_09_P012506	-0.914	2.60.E-03	3.80.E-02
cpo	CG31243_ESCL1_6148	-2.021	2.80.E-21	5.21.E-17	38544	A_09_P041801	-1.041	1.36.E-03	2.83.E-02
					5238	A_09_P225995	-0.873	3.07.E-03	4.08.E-02
cpo	CG31243_ESCL2_1631	-0.914	2.70.E-08	3.77.E-04	38544	A_09_P041801	-1.041	1.36.E-03	2.83.E-02
					5238	A_09_P225995	-0.873	3.07.E-03	4.08.E-02
cpo	CG31243_ESCL3_2132	-1.807	6.44.E-21	1.19.E-16	38544	A_09_P041801	-1.041	1.36.E-03	2.83.E-02
					5238	A_09_P225995	-0.873	3.07.E-03	4.08.E-02
cpo	CG31243_ESCL4_896	-3.694	1.44.E-45	3.20.E-41	38544	A_09_P041801	-1.041	1.36.E-03	2.83.E-02
					5238	A_09_P225995	-0.873	3.07.E-03	4.08.E-02
cpx	CG17299_ESCL3_4138	-1.532	1.19.E-13	1.95.E-09	43734	A_09_P064316	-1.014	2.38.E-03	3.66.E-02
					12609	A_09_P182675	-0.920	3.80.E-03	4.48.E-02
					20944	A_09_P193225	-0.883	1.63.E-03	3.11.E-02
					36183	A_09_P225125	-0.709	4.62.E-03	4.97.E-02

cpx	CG32490_ESCL1_3485	-1.820	8.09.E-08	1.11.E-03	43734	A_09_P064316	-1.014	2.38.E-03	3.66.E-02
					12609	A_09_P182675	-0.920	3.80.E-03	4.48.E-02
					20944	A_09_P193225	-0.883	1.63.E-03	3.11.E-02
					36183	A_09_P225125	-0.709	4.62.E-03	4.97.E-02
d	CG10595_ESCL1_3978	-3.986	3.01.E-43	6.65.E-39	37465	A_09_P179220	-1.168	1.08.E-03	2.52.E-02
					21042	A_09_P041896	-1.029	1.34.E-03	2.82.E-02
d	CG10595_ESCL2_3635	-2.049	2.37.E-30	4.87.E-26	37465	A_09_P179220	-1.168	1.08.E-03	2.52.E-02
					21042	A_09_P041896	-1.029	1.34.E-03	2.82.E-02
dally	CG4974_ESCL1_3365	-4.362	1.28.E-34	2.72.E-30	18003	A_09_P013216	-1.469	1.24.E-03	2.70.E-02
dally	CG4974_ESCL2_1807	-0.446	2.52.E-06	3.23.E-02	18003	A_09_P013216	-1.469	1.24.E-03	2.70.E-02
Dap160	CG1099_ESCL1_3841	-0.568	8.02.E-14	1.31.E-09	10696	A_09_P209195	-0.977	4.08.E-03	4.66.E-02
Dap160	CG1099_ESCL2_3215	-0.396	2.54.E-10	3.79.E-06	10696	A_09_P209195	-0.977	4.08.E-03	4.66.E-02
dib	CG12028_ESCL2_1379	-2.463	3.18.E-32	6.65.E-28	2189	A_09_P141385	-0.897	2.27.E-03	3.60.E-02
dnr1	CG12489_ESCL1_3109	-1.336	9.23.E-21	1.71.E-16	6521	A_09_P026511	-0.785	4.51.E-03	4.91.E-02
dnr1	CG12489_ESCL2_1716	-0.527	2.01.E-06	2.59.E-02	6521	A_09_P026511	-0.785	4.51.E-03	4.91.E-02
Dok	CG2079_ESCL1_3335	-1.836	1.17.E-35	2.51.E-31	837	A_09_P189630	-1.039	2.84.E-03	3.95.E-02
					17607	A_09_P067886	-0.868	2.45.E-03	3.69.E-02
Dok	CG2079_ESCL2_1711	-0.641	8.25.E-09	1.18.E-04	837	A_09_P189630	-1.039	2.84.E-03	3.95.E-02
					17607	A_09_P067886	-0.868	2.45.E-03	3.69.E-02
drl	CG17348_ESCL1_3022	-2.891	4.48.E-33	9.43.E-29	37836	A_09_P030801	-0.801	4.26.E-03	4.76.E-02
drl	CG17348_ESCL2_1758	-2.289	1.94.E-36	4.16.E-32	37836	A_09_P030801	-0.801	4.26.E-03	4.76.E-02
drl	CG17348_ESCL3_2466	-2.823	2.95.E-34	6.26.E-30	37836	A_09_P030801	-0.801	4.26.E-03	4.76.E-02
drongo	CG3365_ESCL2_1745	-0.720	7.33.E-17	1.28.E-12	6441	A_09_P032206	-0.780	4.45.E-03	4.88.E-02
Dys	CG31175_ESCL1_10382	-1.169	3.05.E-19	5.53.E-15	7035	A_09_P045866	-1.956	1.81.E-04	1.14.E-02
					8007	A_09_P091350	-1.119	6.51.E-04	1.96.E-02
Dys	CG31175_ESCL2_9896	-1.610	4.73.E-14	7.78.E-10	7035	A_09_P045866	-1.956	1.81.E-04	1.14.E-02
					8007	A_09_P091350	-1.119	6.51.E-04	1.96.E-02
east	CG4399_ESCL1_7292	-0.964	2.43.E-23	4.64.E-19	36404	A_09_P222800	-0.823	4.17.E-03	4.71.E-02
east	CG4399_ESCL2_6813	-0.937	6.22.E-27	1.24.E-22	36404	A_09_P222800	-0.823	4.17.E-03	4.71.E-02
EcR	CG1765_ESCL1_4635	-3.231	2.69.E-36	5.78.E-32	699	A_09_P206975	-2.031	4.31.E-05	7.50.E-03
					6362	A_09_P051976	-1.700	3.28.E-04	1.45.E-02
					4982	A_09_P042146	-1.636	1.45.E-04	1.05.E-02
					2439	A_09_P042146	-1.579	1.79.E-04	1.14.E-02
					21290	A_09_P050511	-1.457	4.22.E-04	1.59.E-02
EcR	CG1765_ESCL2_2487	-2.044	3.15.E-33	6.63.E-29	699	A_09_P206975	-2.031	4.31.E-05	7.50.E-03
					6362	A_09_P051976	-1.700	3.28.E-04	1.45.E-02
					4982	A_09_P042146	-1.636	1.45.E-04	1.05.E-02
					2439	A_09_P042146	-1.579	1.79.E-04	1.14.E-02
					21290	A_09_P050511	-1.457	4.22.E-04	1.59.E-02
EcR	CG1765_ESCL3_1802	-4.066	2.12.E-40	4.63.E-36	699	A_09_P206975	-2.031	4.31.E-05	7.50.E-03
					6362	A_09_P051976	-1.700	3.28.E-04	1.45.E-02
					4982	A_09_P042146	-1.636	1.45.E-04	1.05.E-02
					2439	A_09_P042146	-1.579	1.79.E-04	1.14.E-02
					21290	A_09_P050511	-1.457	4.22.E-04	1.59.E-02
EcR	CG1765_ESCL4_3438	-2.920	2.65.E-36	5.69.E-32	699	A_09_P206975	-2.031	4.31.E-05	7.50.E-03
					6362	A_09_P051976	-1.700	3.28.E-04	1.45.E-02
					4982	A_09_P042146	-1.636	1.45.E-04	1.05.E-02
					2439	A_09_P042146	-1.579	1.79.E-04	1.14.E-02
					21290	A_09_P050511	-1.457	4.22.E-04	1.59.E-02
Eip75B	CG8127_ESCL1_4421	-1.521	1.27.E-13	2.07.E-09	18320	A_09_P049541	-1.504	3.44.E-04	1.45.E-02
Eip75B	CG8127_ESCL3_4063	-0.625	1.21.E-06	1.58.E-02	18320	A_09_P049541	-1.504	3.44.E-04	1.45.E-02
elav	CG4262_ESCL1_2350	-3.734	1.90.E-41	4.17.E-37	32472	A_09_P090750	-1.450	1.36.E-03	2.83.E-02
					43452	A_09_P121080	-1.027	1.18.E-03	2.65.E-02
					21436	A_09_P042226	-0.824	3.66.E-03	4.45.E-02
					34863	A_09_P188635	-0.740	3.55.E-03	4.36.E-02
elav	CG4262_ESCL2_1301	-2.765	4.22.E-40	9.23.E-36	32472	A_09_P090750	-1.450	1.36.E-03	2.83.E-02
					43452	A_09_P121080	-1.027	1.18.E-03	2.65.E-02
					21436	A_09_P042226	-0.824	3.66.E-03	4.45.E-02
					34863	A_09_P188635	-0.740	3.55.E-03	4.36.E-02
Eno	CG17654_ESCL1_2078	-0.876	6.95.E-14	1.14.E-09	38188	A_09_P042251	-0.929	1.32.E-03	2.79.E-02
					1214	A_09_P042251	-0.883	1.71.E-03	3.20.E-02
Eno	CG17654_ESCL2_1412	-0.640	1.30.E-13	2.12.E-09	38188	A_09_P042251	-0.929	1.32.E-03	2.79.E-02
					1214	A_09_P042251	-0.883	1.71.E-03	3.20.E-02
esn	CG12833_ESCL1_4152	-2.612	5.14.E-34	1.09.E-29	36969	A_09_P134125	-2.078	2.30.E-03	3.63.E-02
					7685	A_09_P065501	-1.918	2.43.E-04	1.26.E-02
Exn	CG3799_ESCL1_3408	-3.275	5.23.E-27	1.04.E-22	24540	A_09_P178980	-1.047	2.33.E-03	3.64.E-02
					13420	A_09_P079726	-0.901	1.91.E-03	3.34.E-02
Exn	CG3799_ESCL2_3035	-1.926	6.53.E-32	1.36.E-27	24540	A_09_P178980	-1.047	2.33.E-03	3.64.E-02
					13420	A_09_P079726	-0.901	1.91.E-03	3.34.E-02

fax	CG4609_ESCL1_2930	-3.604	1.42.E-52	3.19.E-48	37871	A_09_P126520	-1.708	9.12.E-04	2.33.E-02
					6109	A_09_P030211	-1.351	1.09.E-03	2.54.E-02
					23163	A_09_P030211	-1.244	1.07.E-03	2.52.E-02
					13780	A_09_P147645	-1.048	2.10.E-03	3.50.E-02
					43686	A_09_P050171	-0.983	1.78.E-03	3.21.E-02
fax	CG4609_ESCL2_998	-3.320	1.49.E-54	3.35.E-50	37871	A_09_P126520	-1.708	9.12.E-04	2.33.E-02
					6109	A_09_P030211	-1.351	1.09.E-03	2.54.E-02
					23163	A_09_P030211	-1.244	1.07.E-03	2.52.E-02
					13780	A_09_P147645	-1.048	2.10.E-03	3.50.E-02
					43686	A_09_P050171	-0.983	1.78.E-03	3.21.E-02
fax	CG4609_ESCL3_1784	-3.654	1.55.E-54	3.48.E-50	37871	A_09_P126520	-1.708	9.12.E-04	2.33.E-02
					6109	A_09_P030211	-1.351	1.09.E-03	2.54.E-02
					23163	A_09_P030211	-1.244	1.07.E-03	2.52.E-02
					13780	A_09_P147645	-1.048	2.10.E-03	3.50.E-02
					43686	A_09_P050171	-0.983	1.78.E-03	3.21.E-02
Fhos	CG32030_ESCL1_4939	-2.022	5.95.E-18	1.06.E-13	13217	A_09_P047936	-1.871	9.71.E-05	9.21.E-03
Fhos	CG32030_ESCL2_3816	-1.319	9.14.E-18	1.62.E-13	13217	A_09_P047936	-1.871	9.71.E-05	9.21.E-03
Fhos	CG32030_ESCL3_4113	-1.675	4.85.E-31	1.00.E-26	13217	A_09_P047936	-1.871	9.71.E-05	9.21.E-03
Fhos	CG32030_ESCL4_4124	-1.200	8.03.E-17	1.40.E-12	13217	A_09_P047936	-1.871	9.71.E-05	9.21.E-03
fj	CG10917_ESCL1_3449	-4.453	4.04.E-27	8.04.E-23	27168	A_09_P042371	-3.617	2.87.E-04	1.35.E-02
					9710	A_09_P042371	-3.564	2.48.E-04	1.27.E-02
					20441	A_09_P121565	-3.130	2.59.E-04	1.29.E-02
fj	CG10917_ESCL2_1649	-2.657	7.82.E-33	1.64.E-28	27168	A_09_P042371	-3.617	2.87.E-04	1.35.E-02
					9710	A_09_P042371	-3.564	2.48.E-04	1.27.E-02
					20441	A_09_P121565	-3.130	2.59.E-04	1.29.E-02
fng	CG10580_ESCL1_1802	-1.513	2.52.E-20	4.64.E-16	5908	A_09_P013246	-2.826	9.97.E-05	9.34.E-03
					6182	A_09_P013246	-2.781	2.04.E-03	3.45.E-02
fng	CG10580_ESCL2_970	-1.566	6.33.E-21	1.17.E-16	5908	A_09_P013246	-2.826	9.97.E-05	9.34.E-03
					6182	A_09_P013246	-2.781	2.04.E-03	3.45.E-02
for	CG10033_ESCL1_4464	-1.281	2.75.E-28	5.55.E-24	4490	A_09_P042411	-0.993	1.53.E-03	3.00.E-02
					4302	A_09_P042411	-0.937	2.11.E-03	3.50.E-02
for	CG10033_ESCL2_3177	-0.988	1.50.E-20	2.76.E-16	4490	A_09_P042411	-0.993	1.53.E-03	3.00.E-02
					4302	A_09_P042411	-0.937	2.11.E-03	3.50.E-02
for	CG10033_ESCL3_3984	-1.198	7.98.E-25	1.55.E-20	4490	A_09_P042411	-0.993	1.53.E-03	3.00.E-02
					4302	A_09_P042411	-0.937	2.11.E-03	3.50.E-02
fs(1)K10	CG3218_ESCL1_2991	-1.412	2.20.E-27	4.40.E-23	13816	A_09_P042426	-4.190	2.72.E-07	3.55.E-03
					27524	A_09_P042426	-4.089	2.87.E-07	3.55.E-03
fs(1)K10	CG3218_ESCL2_1320	-1.738	2.73.E-18	4.87.E-14	13816	A_09_P042426	-4.190	2.72.E-07	3.55.E-03
					27524	A_09_P042426	-4.089	2.87.E-07	3.55.E-03
fz3	CG16785_ESCL2_1671	-0.564	1.83.E-09	2.66.E-05	28100	A_09_P079251	-1.240	1.26.E-03	2.71.E-02
					32287	A_09_P199375	-0.947	1.12.E-03	2.56.E-02
G-salpha60A	CG2835_ESCL3_907	-1.474	4.19.E-30	8.60.E-26	44066	A_09_P042581	-1.068	3.61.E-03	4.40.E-02
					12451	A_09_P042581	-1.022	1.56.E-03	3.04.E-02
					8655	A_09_P196460	-0.788	4.66.E-03	4.99.E-02
G-salpha60A	CG2835_ESCL4_907	-1.381	1.38.E-26	2.73.E-22	44066	A_09_P042581	-1.068	3.61.E-03	4.40.E-02
					12451	A_09_P042581	-1.022	1.56.E-03	3.04.E-02
					8655	A_09_P196460	-0.788	4.66.E-03	4.99.E-02
galectin	CG11372_ESCL1_1449	-0.815	1.36.E-14	2.26.E-10	16459	A_09_P122725	-1.641	8.82.E-05	8.90.E-03
					20671	A_09_P017791	-1.635	1.98.E-04	1.18.E-02
Gasp	CG10287_ESCL1_1206	-1.130	8.16.E-17	1.42.E-12	15864	A_09_P078086	-1.023	8.96.E-04	2.30.E-02
Gasp	CG10287_ESCL2_691	-2.201	1.81.E-23	3.47.E-19	15864	A_09_P078086	-1.023	8.96.E-04	2.30.E-02
gbb	CG5562_ESCL1_1277	-2.272	2.13.E-39	4.64.E-35	44930	A_09_P076726	-0.876	1.79.E-03	3.22.E-02
					37890	A_09_P076726	-0.824	2.30.E-03	3.63.E-02
Gli	CG3903_ESCL1_3875	-2.249	1.70.E-31	3.53.E-27	16331	A_09_P043156	-1.531	7.13.E-04	2.04.E-02
					6738	A_09_P043156	-1.510	1.45.E-03	2.92.E-02
					16041	A_09_P144150	-1.129	1.80.E-03	3.23.E-02
gol	CG2679_ESCL1_2485	-0.884	1.06.E-12	1.69.E-08	25769	A_09_P146635	-1.331	3.33.E-03	4.24.E-02
					42537	A_09_P011351	-1.289	2.56.E-03	3.77.E-02
gol	CG2679_ESCL2_2309	-1.925	7.67.E-21	1.42.E-16	25769	A_09_P146635	-1.331	3.33.E-03	4.24.E-02
					42537	A_09_P011351	-1.289	2.56.E-03	3.77.E-02
Gp150	CG5820_ESCL1_4112	-2.963	1.49.E-49	3.33.E-45	33169	A_09_P116275	-0.764	4.66.E-03	4.99.E-02
					28300	A_09_P029566	-0.758	3.48.E-03	4.31.E-02
Gp150	CG5820_ESCL2_3039	-1.813	4.43.E-30	9.09.E-26	33169	A_09_P116275	-0.764	4.66.E-03	4.99.E-02
					28300	A_09_P029566	-0.758	3.48.E-03	4.31.E-02
GstD1	CG10045_ESCL1_477	-2.205	3.12.E-32	6.52.E-28	757	A_09_P042646	-3.246	9.49.E-06	5.81.E-03
					27083	A_09_P042646	-3.059	7.74.E-06	5.81.E-03
GstD10	CG18548_ESCL1_561	-1.391	1.50.E-22	2.85.E-18	24830	A_09_P064681	-3.535	3.93.E-05	7.50.E-03
					10299	A_09_P209530	-3.513	1.12.E-04	9.54.E-03
GstD2	CG4181_ESCL1_575	-1.015	3.45.E-07	4.61.E-03	35205	A_09_P147905	-1.801	1.28.E-04	1.01.E-02

					17233	A_09_P011881	-1.615	2.55.E-04	1.29.E-02
					30486	A_09_P011881	-1.521	2.99.E-04	1.38.E-02
GstD9	CG10091_ESCL1_592	-0.510	3.25.E-15	5.48.E-11	36371	A_09_P074286	-1.923	4.26.E-05	7.50.E-03
GstE6	CG17530_ESCL1_533	-1.903	1.06.E-36	2.27.E-32	15967	A_09_P001976	-2.745	2.07.E-05	6.43.E-03
					1571	A_09_P001976	-2.552	5.40.E-05	7.82.E-03
GstE7	CG17531_ESCL1_606	-1.806	9.41.E-44	2.08.E-39	6218	A_09_P001971	-1.937	3.41.E-05	7.50.E-03
					39291	A_09_P001971	-1.914	4.78.E-05	7.55.E-03
GstE8	CG17533_ESCL1_1098	-0.718	7.55.E-07	9.93.E-03	24607	A_09_P001966	-0.884	3.11.E-03	4.10.E-02
GstE8	CG17533_ESCL2_541	-2.328	3.76.E-24	7.26.E-20	24607	A_09_P001966	-0.884	3.11.E-03	4.10.E-02
GstE9	CG17533_ESCL3_1875	-2.314	1.77.E-31	3.69.E-27	27135	A_09_P001961	-1.028	9.85.E-04	2.41.E-02
					15744	A_09_P001961	-0.920	1.27.E-03	2.72.E-02
GstE9	CG17534_ESCL1_565	-1.421	5.64.E-24	1.09.E-19	27135	A_09_P001961	-1.028	9.85.E-04	2.41.E-02
					15744	A_09_P001961	-0.920	1.27.E-03	2.72.E-02
H2.0	CG11607_ESCL1_1595	-3.264	3.73.E-16	6.41.E-12	28508	A_09_P042671	-1.004	2.34.E-03	3.64.E-02
H2.0	CG11607_ESCL2_1146	-0.748	1.85.E-13	3.01.E-09	28508	A_09_P042671	-1.004	2.34.E-03	3.64.E-02
hig	CG2040_ESCL1_3831	-0.997	4.29.E-09	6.17.E-05	40394	A_09_P011971	-2.042	2.54.E-03	3.77.E-02
					724	A_09_P011971	-1.878	1.37.E-03	2.83.E-02
hipk	CG17090_ESCL1_6210	-0.991	1.04.E-16	1.80.E-12	34739	A_09_P188060	-0.989	2.92.E-03	4.00.E-02
					35996	A_09_P028001	-0.989	2.06.E-03	3.46.E-02
					19159	A_09_P028001	-0.705	4.71.E-03	5.00.E-02
hipk	CG17090_ESCL3_2769	-1.042	6.22.E-19	1.12.E-14	34739	A_09_P188060	-0.989	2.92.E-03	4.00.E-02
					35996	A_09_P028001	-0.989	2.06.E-03	3.46.E-02
					19159	A_09_P028001	-0.705	4.71.E-03	5.00.E-02
HLH4C	CG3052_ESCL1_1240	-0.807	3.55.E-07	4.75.E-03	35401	A_09_P013011	-0.797	3.02.E-03	4.05.E-02
HLHm7	CG8361_ESCL1_468	-1.667	2.10.E-16	3.63.E-12	35451	A_09_P180645	-1.225	3.60.E-04	1.47.E-02
Hsp22	CG32041_ESCL4_255	-2.579	1.80.E-28	3.64.E-24	40043	A_09_P042786	-0.801	2.42.E-03	3.69.E-02
					22406	A_09_P042786	-0.717	4.11.E-03	4.69.E-02
Idgf4	CG1780_ESCL1_1486	-3.333	2.01.E-42	4.44.E-38	40411	A_09_P078611	-0.788	3.09.E-03	4.09.E-02
Idgf4	CG1780_ESCL2_1203	-3.063	1.37.E-54	3.08.E-50	40411	A_09_P078611	-0.788	3.09.E-03	4.09.E-02
inx2	CG4590_ESCL1_2018	-4.202	3.90.E-64	8.81.E-60	381	A_09_P079136	-0.807	2.55.E-03	3.77.E-02
					5075	A_09_P079136	-0.776	2.87.E-03	3.97.E-02
inx2	CG4590_ESCL2_1004	-2.708	1.25.E-48	2.80.E-44	381	A_09_P079136	-0.807	2.55.E-03	3.77.E-02
					5075	A_09_P079136	-0.776	2.87.E-03	3.97.E-02
inx3	CG1448_ESCL1_1495	-5.487	1.67.E-49	3.74.E-45	9408	A_09_P064886	-1.105	2.42.E-03	3.69.E-02
inx3	CG1448_ESCL2_767	-2.636	4.84.E-37	1.04.E-32	9408	A_09_P064886	-1.105	2.42.E-03	3.69.E-02
jar	CG5695_ESCL1_4293	-1.833	1.36.E-33	2.88.E-29	44306	A_09_P012921	-0.809	2.43.E-03	3.69.E-02
jar	CG5695_ESCL2_3690	-1.555	8.61.E-38	1.87.E-33	44306	A_09_P012921	-0.809	2.43.E-03	3.69.E-02
JhI-26	CG3767_ESCL1_1245	-1.971	1.30.E-39	2.85.E-35	9433	A_09_P065051	-1.725	2.04.E-04	1.19.E-02
kek5	CG12199_ESCL1_4041	-1.902	2.54.E-39	5.54.E-35	37568	A_09_P040471	-1.160	3.71.E-03	4.46.E-02
					11922	A_09_P172690	-0.943	3.88.E-03	4.54.E-02
Kmn1	CG1558_ESCL1_929	-1.216	2.14.E-17	3.76.E-13	37594	A_09_P079151	-1.803	6.55.E-05	8.27.E-03
					12874	A_09_P079151	-1.764	3.99.E-05	7.50.E-03
Kmn1	CG1558_ESCL2_464	-1.476	1.25.E-24	2.42.E-20	37594	A_09_P079151	-1.803	6.55.E-05	8.27.E-03
					12874	A_09_P079151	-1.764	3.99.E-05	7.50.E-03
kni	CG4717_ESCL1_1901	-3.299	9.73.E-38	2.11.E-33	9631	A_09_P042996	-1.036	1.22.E-03	2.68.E-02
kni	CG4717_ESCL2_1218	-1.617	4.88.E-29	9.92.E-25	9631	A_09_P042996	-1.036	1.22.E-03	2.68.E-02
Kr	CG3340_ESCL1_2165	-4.242	2.68.E-52	6.01.E-48	32332	A_09_P043016	-1.151	4.17.E-03	4.71.E-02
Kr	CG3340_ESCL2_1446	-1.594	5.76.E-28	1.16.E-23	32332	A_09_P043016	-1.151	4.17.E-03	4.71.E-02
l(2)01810	CG5304_ESCL1_2203	-0.831	6.50.E-13	1.04.E-08	28619	A_09_P012596	-0.810	4.40.E-03	4.85.E-02
l(3)neo38	CG31364_ESCL1_631	-2.223	1.76.E-25	3.45.E-21	13946	A_09_P050936	-0.980	1.08.E-03	2.53.E-02
					36780	A_09_P116250	-0.930	1.87.E-03	3.30.E-02
					22967	A_09_P203805	-0.845	3.19.E-03	4.15.E-02
					42411	A_09_P126260	-0.806	3.83.E-03	4.50.E-02
l(3)neo38	CG6930_ESCL1_1429	-1.738	2.96.E-39	6.46.E-35	13946	A_09_P050936	-0.980	1.08.E-03	2.53.E-02
					36780	A_09_P116250	-0.930	1.87.E-03	3.30.E-02
					22967	A_09_P203805	-0.845	3.19.E-03	4.15.E-02
					42411	A_09_P126260	-0.806	3.83.E-03	4.50.E-02
l(3)neo38	CG6930_ESCL2_942	-0.996	1.87.E-08	2.63.E-04	13946	A_09_P050936	-0.980	1.08.E-03	2.53.E-02
					36780	A_09_P116250	-0.930	1.87.E-03	3.30.E-02
					22967	A_09_P203805	-0.845	3.19.E-03	4.15.E-02
					42411	A_09_P126260	-0.806	3.83.E-03	4.50.E-02
lama	CG10645_ESCL1_3326	-1.650	6.53.E-26	1.29.E-21	31816	A_09_P031426	-1.527	5.40.E-04	1.79.E-02
					37163	A_09_P031426	-1.491	1.00.E-03	2.43.E-02
lama	CG10645_ESCL2_1809	-2.155	2.45.E-30	5.05.E-26	31816	A_09_P031426	-1.527	5.40.E-04	1.79.E-02
					37163	A_09_P031426	-1.491	1.00.E-03	2.43.E-02
LanA	CG10236_ESCL1_11482	-4.374	1.36.E-58	3.08.E-54	17648	A_09_P043306	-1.674	6.63.E-05	8.27.E-03
LanA	CG10236_ESCL2_11025	-3.569	3.26.E-55	7.33.E-51	17648	A_09_P043306	-1.674	6.63.E-05	8.27.E-03
LanB1	CG7123_ESCL1_6103	-1.287	1.34.E-23	2.56.E-19	15218	A_09_P043311	-0.767	4.03.E-03	4.64.E-02
					40410	A_09_P043311	-0.753	3.33.E-03	4.24.E-02

LanB1	CG7123_ESCL2_5185	-1.027	2.45.E-23	4.68.E-19	15218	A_09_P043311	-0.767	4.03.E-03	4.64.E-02
					40410	A_09_P043311	-0.753	3.33.E-03	4.24.E-02
lbm	CG2374_ESCL1_937	-2.091	8.38.E-30	1.72.E-25	10847	A_09_P031431	-1.307	2.07.E-04	1.19.E-02
					28657	A_09_P031431	-1.220	5.81.E-04	1.85.E-02
lbm	CG2374_ESCL2_526	-0.831	5.11.E-11	7.79.E-07	10847	A_09_P031431	-1.307	2.07.E-04	1.19.E-02
					28657	A_09_P031431	-1.220	5.81.E-04	1.85.E-02
lea	CG5481_ESCL1_1373	-3.468	1.48.E-41	3.25.E-37	18419	A_09_P043346	-2.354	1.01.E-05	5.81.E-03
					25827	A_09_P043346	-2.171	2.51.E-05	6.83.E-03
					32337	A_09_P133790	-1.342	4.13.E-04	1.57.E-02
lea	CG5481_ESCL2_3967	-1.125	2.62.E-17	4.60.E-13	18419	A_09_P043346	-2.354	1.01.E-05	5.81.E-03
					25827	A_09_P043346	-2.171	2.51.E-05	6.83.E-03
					32337	A_09_P133790	-1.342	4.13.E-04	1.57.E-02
Lk6	CG17342_ESCL1_4362	-0.594	5.65.E-11	8.62.E-07	25726	A_09_P117490	-0.820	2.38.E-03	3.66.E-02
					32715	A_09_P031896	-0.717	4.70.E-03	5.00.E-02
lmd	CG4677_ESCL1_2938	-2.857	1.69.E-42	3.72.E-38	19930	A_09_P070481	-1.077	2.62.E-03	3.81.E-02
lmd	CG4677_ESCL2_1573	-1.732	1.32.E-23	2.53.E-19	19930	A_09_P070481	-1.077	2.62.E-03	3.81.E-02
lmd	CG4677_ESCL3_2528	-2.481	5.02.E-37	1.08.E-32	19930	A_09_P070481	-1.077	2.62.E-03	3.81.E-02
mbl	CG33197_ESCL2_3679	-0.809	4.23.E-12	6.65.E-08	23879	A_09_P017096	-1.131	2.38.E-03	3.66.E-02
melt	CG8624_ESCL1_2819	-1.121	5.54.E-18	9.84.E-14	5535	A_09_P045646	-1.077	2.66.E-03	3.83.E-02
Men	CG10120_ESCL1_2861	-1.752	6.09.E-27	1.21.E-22	5584	A_09_P043541	-2.284	8.37.E-06	5.81.E-03
					31172	A_09_P091290	-2.268	2.65.E-05	6.86.E-03
					15773	A_09_P090755	-1.215	4.45.E-04	1.63.E-02
Men	CG10120_ESCL2_2212	-1.332	4.90.E-35	1.04.E-30	5584	A_09_P043541	-2.284	8.37.E-06	5.81.E-03
					31172	A_09_P091290	-2.268	2.65.E-05	6.86.E-03
					15773	A_09_P090755	-1.215	4.45.E-04	1.63.E-02
miple2	CG18321_ESCL1_778	-3.306	6.64.E-46	1.48.E-41	10819	A_09_P066351	-1.760	6.51.E-05	8.27.E-03
					2898	A_09_P066351	-1.674	1.39.E-04	1.04.E-02
mnd	CG3297_ESCL1_1845	-2.309	8.28.E-37	1.78.E-32	36460	A_09_P154825	-1.592	2.42.E-04	1.26.E-02
					1709	A_09_P043611	-1.535	3.14.E-04	1.42.E-02
					26687	A_09_P043611	-1.514	3.41.E-04	1.45.E-02
mnd	CG3297_ESCL2_1423	-1.401	3.39.E-27	6.74.E-23	36460	A_09_P154825	-1.592	2.42.E-04	1.26.E-02
					1709	A_09_P043611	-1.535	3.14.E-04	1.42.E-02
					26687	A_09_P043611	-1.514	3.41.E-04	1.45.E-02
modSP	CG31217_ESCL1_2280	-0.530	1.57.E-07	2.13.E-03	36166	A_09_P059336	-2.181	5.34.E-05	7.82.E-03
					27668	A_09_P059336	-1.856	1.02.E-04	9.38.E-03
					4568	A_09_P198635	-1.453	1.58.E-04	1.07.E-02
modSP	CG31217_ESCL2_1824	-0.562	7.15.E-11	1.09.E-06	36166	A_09_P059336	-2.181	5.34.E-05	7.82.E-03
					27668	A_09_P059336	-1.856	1.02.E-04	9.38.E-03
					4568	A_09_P198635	-1.453	1.58.E-04	1.07.E-02
Msp-300	CG14035_ESCL1_753	-1.071	1.09.E-10	1.64.E-06	4398	A_09_P050461	-2.343	1.39.E-04	1.04.E-02
					20893	A_09_P050976	-1.797	4.73.E-05	7.55.E-03
					38664	A_09_P166080	-1.282	4.36.E-03	4.82.E-02
					29967	A_09_P046011	-1.060	5.98.E-04	1.89.E-02
Msp-300	CG31649_ESCL1_11903	-1.887	2.57.E-31	5.34.E-27	4398	A_09_P050461	-2.343	1.39.E-04	1.04.E-02
					20893	A_09_P050976	-1.797	4.73.E-05	7.55.E-03
					38664	A_09_P166080	-1.282	4.36.E-03	4.82.E-02
					29967	A_09_P046011	-1.060	5.98.E-04	1.89.E-02
Msp-300	CG31916_ESCL2_837	-3.239	4.16.E-37	8.97.E-33	4398	A_09_P050461	-2.343	1.39.E-04	1.04.E-02
					20893	A_09_P050976	-1.797	4.73.E-05	7.55.E-03
					38664	A_09_P166080	-1.282	4.36.E-03	4.82.E-02
					29967	A_09_P046011	-1.060	5.98.E-04	1.89.E-02
msta	CG32800_ESCL1_1683	-0.924	2.34.E-06	3.01.E-02	15352	A_09_P000246	-2.228	1.10.E-04	9.52.E-03
msta	CG32800_ESCL2_1310	-0.340	2.46.E-09	3.57.E-05	15352	A_09_P000246	-2.228	1.10.E-04	9.52.E-03
mthl5	CG6965_ESCL1_1257	-1.154	3.40.E-20	6.24.E-16	39585	A_09_P074051	-1.285	2.08.E-04	1.19.E-02
					44144	A_09_P074051	-1.204	6.11.E-04	1.91.E-02
Myo10A	CG2174_ESCL1_8387	-0.832	4.62.E-18	8.21.E-14	14782	A_09_P037516	-1.151	2.09.E-03	3.50.E-02
Myo10A	CG2174_ESCL2_7638	-0.800	6.85.E-16	1.17.E-11	14782	A_09_P037516	-1.151	2.09.E-03	3.50.E-02
myoglianin	CG1838_ESCL2_1725	-1.239	8.01.E-25	1.56.E-20	9219	A_09_P078306	-1.024	1.64.E-03	3.11.E-02
					24237	A_09_P078306	-0.962	1.15.E-03	2.61.E-02
myoglianin	CG1838_ESCL4_2703	-1.065	1.61.E-07	2.18.E-03	9219	A_09_P078306	-1.024	1.64.E-03	3.11.E-02
					24237	A_09_P078306	-0.962	1.15.E-03	2.61.E-02
mys	CG1560_ESCL1_3705	-0.524	1.08.E-07	1.48.E-03	43489	A_09_P010991	-0.954	2.02.E-03	3.43.E-02
					43249	A_09_P010991	-0.953	1.71.E-03	3.20.E-02
N	CG3936_ESCL1_9849	-1.964	8.62.E-30	1.76.E-25	8672	A_09_P010921	-1.163	6.70.E-04	1.99.E-02
					11845	A_09_P050251	-0.908	1.93.E-03	3.36.E-02
N	CG3936_ESCL2_8049	-1.830	6.43.E-35	1.37.E-30	8672	A_09_P010921	-1.163	6.70.E-04	1.99.E-02
					11845	A_09_P050251	-0.908	1.93.E-03	3.36.E-02
Ndae1	CG4675_ESCL1_3030	-1.040	8.19.E-16	1.40.E-11	17784	A_09_P020306	-0.850	2.06.E-03	3.46.E-02
nerfin-1	CG13906_ESCL1_2930	-4.586	8.62.E-29	1.75.E-24	38817	A_09_P066346	-1.001	3.55.E-03	4.36.E-02

net	CG11450_ESCL1_2119	-3.165	1.16.E-25	2.28.E-21	40137	A_09_P043836	-2.030	4.44.E-05	7.50.E-03
					22128	A_09_P043836	-1.643	5.56.E-05	7.90.E-03
					33254	A_09_P104200	-1.272	4.18.E-04	1.58.E-02
Nhe2	CG9256_ESCL1_3725	-0.563	7.02.E-10	1.04.E-05	43639	A_09_P189445	-1.254	2.60.E-04	1.29.E-02
					43111	A_09_P045726	-1.243	2.78.E-04	1.34.E-02
noc	CG4491_ESCL1_2721	-3.286	1.90.E-32	3.98.E-28	33758	A_09_P011806	-2.538	1.35.E-05	6.14.E-03
					22078	A_09_P011806	-2.519	1.14.E-05	5.91.E-03
noc	CG4491_ESCL2_1551	-4.259	1.38.E-52	3.10.E-48	33758	A_09_P011806	-2.538	1.35.E-05	6.14.E-03
					22078	A_09_P011806	-2.519	1.14.E-05	5.91.E-03
noc	CG4491_ESCL3_2334	-4.097	4.00.E-50	8.96.E-46	33758	A_09_P011806	-2.538	1.35.E-05	6.14.E-03
					22078	A_09_P011806	-2.519	1.14.E-05	5.91.E-03
Nplp2	CG11051_ESCL1_369	-4.531	1.84.E-53	4.14.E-49	26788	A_09_P047146	-1.032	2.22.E-03	3.59.E-02
Nplp2	CG11051_ESCL2_153	-5.041	4.21.E-51	9.45.E-47	26788	A_09_P047146	-1.032	2.22.E-03	3.59.E-02
nullo	CG14426_ESCL1_755	-3.474	3.85.E-32	8.04.E-28	7651	A_09_P010186	-0.943	4.13.E-03	4.69.E-02
nullo	CG14426_ESCL2_550	-2.223	1.55.E-29	3.17.E-25	7651	A_09_P010186	-0.943	4.13.E-03	4.69.E-02
Oatp30B	CG3811_ESCL1_5300	-2.650	5.28.E-44	1.17.E-39	38295	A_09_P021126	-1.223	1.33.E-03	2.80.E-02
					38992	A_09_P021126	-1.191	1.02.E-03	2.45.E-02
Oatp30B	CG3811_ESCL2_3508	-1.438	2.11.E-19	3.84.E-15	38295	A_09_P021126	-1.223	1.33.E-03	2.80.E-02
					38992	A_09_P021126	-1.191	1.02.E-03	2.45.E-02
odd	CG3851_ESCL1_2226	-3.192	8.62.E-41	1.89.E-36	22692	A_09_P131435	-1.666	1.42.E-04	1.04.E-02
odd	CG3851_ESCL2_1110	-2.404	1.20.E-34	2.55.E-30	22692	A_09_P131435	-1.666	1.42.E-04	1.04.E-02
ogre	CG3039_ESCL1_2053	-2.262	4.99.E-39	1.09.E-34	16526	A_09_P143495	-1.800	4.85.E-04	1.69.E-02
					36047	A_09_P010916	-1.641	1.42.E-04	1.04.E-02
					10474	A_09_P010916	-1.551	2.18.E-04	1.22.E-02
					33570	A_09_P135225	-1.319	3.07.E-04	1.40.E-02
ogre	CG3039_ESCL2_996	-1.705	8.62.E-32	1.80.E-27	16526	A_09_P143495	-1.800	4.85.E-04	1.69.E-02
					36047	A_09_P010916	-1.641	1.42.E-04	1.04.E-02
					10474	A_09_P010916	-1.551	2.18.E-04	1.22.E-02
					33570	A_09_P135225	-1.319	3.07.E-04	1.40.E-02
Orct2	CG13610_ESCL1_2137	-3.144	1.21.E-29	2.47.E-25	35907	A_09_P048681	-1.445	1.22.E-04	9.96.E-03
					18219	A_09_P048681	-1.351	4.11.E-04	1.57.E-02
Orct2	CG13610_ESCL2_1625	-3.398	5.45.E-30	1.12.E-25	35907	A_09_P048681	-1.445	1.22.E-04	9.96.E-03
					18219	A_09_P048681	-1.351	4.11.E-04	1.57.E-02
pb	CG31481_ESCL2_3489	-0.774	7.28.E-07	9.58.E-03	29130	A_09_P151010	-1.581	8.84.E-04	2.28.E-02
Pka-C3	CG6117_ESCL1_2077	-1.789	1.78.E-22	3.37.E-18	42040	A_09_P042051	-2.445	3.68.E-04	1.48.E-02
					12679	A_09_P042051	-2.369	1.07.E-04	9.43.E-03
Pka-C3	CG6117_ESCL3_1855	-2.019	1.23.E-24	2.39.E-20	42040	A_09_P042051	-2.445	3.68.E-04	1.48.E-02
					12679	A_09_P042051	-2.369	1.07.E-04	9.43.E-03
pnt	CG17077_ESCL1_3048	-0.811	1.56.E-15	2.64.E-11	24625	A_09_P135145	-1.046	1.73.E-03	3.20.E-02
					62	A_09_P044171	-1.039	2.45.E-03	3.69.E-02
					31865	A_09_P142265	-1.028	2.45.E-03	3.69.E-02
					42973	A_09_P044171	-0.938	3.73.E-03	4.46.E-02
					31510	A_09_P044171	-0.918	4.13.E-03	4.69.E-02
					4671	A_09_P044171	-0.901	4.42.E-03	4.86.E-02
					42528	A_09_P044171	-0.874	3.67.E-03	4.45.E-02
pnt	CG17077_ESCL2_2095	-0.576	1.02.E-10	1.54.E-06	24625	A_09_P135145	-1.046	1.73.E-03	3.20.E-02
					62	A_09_P044171	-1.039	2.45.E-03	3.69.E-02
					31865	A_09_P142265	-1.028	2.45.E-03	3.69.E-02
					42973	A_09_P044171	-0.938	3.73.E-03	4.46.E-02
					31510	A_09_P044171	-0.918	4.13.E-03	4.69.E-02
					4671	A_09_P044171	-0.901	4.42.E-03	4.86.E-02
					42528	A_09_P044171	-0.874	3.67.E-03	4.45.E-02
pot	CG2467_ESCL1_3679	-2.426	1.88.E-27	3.76.E-23	27576	A_09_P128510	-1.981	4.32.E-04	1.61.E-02
Prx2540-2	CG11765_ESCL1_601	-2.723	1.32.E-50	2.96.E-46	3483	A_09_P006101	-1.169	4.91.E-04	1.70.E-02
ps	CG16765_ESCL1_706	-2.257	1.77.E-48	3.97.E-44	40458	A_09_P193630	-1.299	6.95.E-04	2.02.E-02
					20817	A_09_P078251	-1.083	1.42.E-03	2.88.E-02
					7683	A_09_P078251	-1.031	1.25.E-03	2.71.E-02
					38290	A_09_P078256	-0.956	3.73.E-03	4.46.E-02
					33554	A_09_P179165	-0.943	3.24.E-03	4.18.E-02
					8422	A_09_P136435	-0.817	4.25.E-03	4.75.E-02
ps	CG16777_ESCL1_491	-1.328	6.47.E-23	1.23.E-18	40458	A_09_P193630	-1.299	6.95.E-04	2.02.E-02
					20817	A_09_P078251	-1.083	1.42.E-03	2.88.E-02
					7683	A_09_P078251	-1.031	1.25.E-03	2.71.E-02
					38290	A_09_P078256	-0.956	3.73.E-03	4.46.E-02
					33554	A_09_P179165	-0.943	3.24.E-03	4.18.E-02
					8422	A_09_P136435	-0.817	4.25.E-03	4.75.E-02
ps	CG8144_ESCL1_1656	-2.819	1.08.E-32	2.26.E-28	40458	A_09_P193630	-1.299	6.95.E-04	2.02.E-02
					20817	A_09_P078251	-1.083	1.42.E-03	2.88.E-02
					7683	A_09_P078251	-1.031	1.25.E-03	2.71.E-02

					38290	A_09_P078256	-0.956	3.73.E-03	4.46.E-02
					33554	A_09_P179165	-0.943	3.24.E-03	4.18.E-02
					8422	A_09_P136435	-0.817	4.25.E-03	4.75.E-02
ps	CG8144_ESCL3_1348	-2.082	2.37.E-18	4.23.E-14	40458	A_09_P193630	-1.299	6.95.E-04	2.02.E-02
					20817	A_09_P078251	-1.083	1.42.E-03	2.88.E-02
					7683	A_09_P078251	-1.031	1.25.E-03	2.71.E-02
					38290	A_09_P078256	-0.956	3.73.E-03	4.46.E-02
					33554	A_09_P179165	-0.943	3.24.E-03	4.18.E-02
					8422	A_09_P136435	-0.817	4.25.E-03	4.75.E-02
puc	CG7850_ESCL1_2836	-0.534	1.59.E-09	2.31.E-05	39500	A_09_P010281	-0.908	2.88.E-03	3.97.E-02
pum	CG9755_ESCL1_6549	-2.222	8.54.E-33	1.79.E-28	32261	A_09_P181770	-1.434	6.80.E-04	2.00.E-02
					32660	A_09_P044261	-0.822	2.92.E-03	4.00.E-02
pum	CG9755_ESCL2_4254	-0.872	3.01.E-12	4.75.E-08	32261	A_09_P181770	-1.434	6.80.E-04	2.00.E-02
					32660	A_09_P044261	-0.822	2.92.E-03	4.00.E-02
Pvf2	CG13780_ESCL1_1973	-0.507	2.13.E-06	2.74.E-02	31772	A_09_P020266	-2.376	1.81.E-05	6.20.E-03
					34312	A_09_P020266	-2.329	1.38.E-05	6.14.E-03
					24728	A_09_P121465	-2.103	3.62.E-05	7.50.E-03
Pvf2	CG13780_ESCL2_1152	-0.534	2.08.E-06	2.68.E-02	31772	A_09_P020266	-2.376	1.81.E-05	6.20.E-03
					34312	A_09_P020266	-2.329	1.38.E-05	6.14.E-03
					24728	A_09_P121465	-2.103	3.62.E-05	7.50.E-03
Pvf3	CG31629_ESCL2_1361	-1.292	4.37.E-13	7.05.E-09	21703	A_09_P121460	-2.466	1.93.E-04	1.17.E-02
					671	A_09_P164230	-1.395	1.96.E-04	1.17.E-02
pyd3	CG3027_ESCL1_1094	-2.865	5.53.E-47	1.23.E-42	23787	A_09_P037061	-1.093	6.08.E-04	1.91.E-02
					6658	A_09_P037061	-0.977	1.23.E-03	2.70.E-02
qtc	CG14039_ESCL1_2260	-0.960	1.93.E-10	2.91.E-06	27132	A_09_P065466	-1.028	1.08.E-03	2.53.E-02
					9278	A_09_P065466	-1.011	2.51.E-03	3.75.E-02
					19137	A_09_P153140	-0.945	1.12.E-03	2.56.E-02
Rab8	CG8287_ESCL1_2081	-2.751	2.71.E-30	5.57.E-26	3580	A_09_P134980	-0.770	3.57.E-03	4.37.E-02
Rab8	CG8287_ESCL2_465	-2.259	3.59.E-34	7.60.E-30	3580	A_09_P134980	-0.770	3.57.E-03	4.37.E-02
Rab8	CG8287_ESCL3_1941	-0.902	6.34.E-16	1.09.E-11	3580	A_09_P134980	-0.770	3.57.E-03	4.37.E-02
ref(2)P	CG10360_ESCL1_3533	-0.756	4.90.E-21	9.10.E-17	12557	A_09_P044376	-0.931	2.36.E-03	3.64.E-02
					22119	A_09_P044376	-0.857	2.44.E-03	3.69.E-02
					8035	A_09_P198585	-0.755	3.73.E-03	4.46.E-02
ref(2)P	CG10360_ESCL2_1641	-0.597	3.64.E-12	5.73.E-08	12557	A_09_P044376	-0.931	2.36.E-03	3.64.E-02
					22119	A_09_P044376	-0.857	2.44.E-03	3.69.E-02
					8035	A_09_P198585	-0.755	3.73.E-03	4.46.E-02
rpr	CG4319_ESCL1_493	-0.689	4.74.E-09	6.80.E-05	44758	A_09_P013426	-2.127	2.60.E-04	1.29.E-02
Rtnl1	CG33113_ESCL1_2617	-2.658	1.16.E-24	2.25.E-20	26584	A_09_P016851	-0.878	2.99.E-03	4.02.E-02
					6145	A_09_P136610	-0.792	3.39.E-03	4.26.E-02
Rtnl1	CG33113_ESCL2_1689	-2.546	3.29.E-32	6.88.E-28	26584	A_09_P016851	-0.878	2.99.E-03	4.02.E-02
					6145	A_09_P136610	-0.792	3.39.E-03	4.26.E-02
Rya-r44F	CG10844_ESCL1_15708	-3.228	6.50.E-36	1.39.E-31	13843	A_09_P102745	-1.671	2.97.E-04	1.37.E-02
Rya-r44F	CG10844_ESCL2_15181	-1.848	1.55.E-31	3.22.E-27	13843	A_09_P102745	-1.671	2.97.E-04	1.37.E-02
scb	CG8095_ESCL1_4260	-2.643	1.86.E-36	4.00.E-32	29576	A_09_P044561	-1.539	1.56.E-04	1.07.E-02
					4119	A_09_P187265	-1.526	2.84.E-04	1.34.E-02
					7101	A_09_P044561	-1.501	3.98.E-04	1.55.E-02
scyl	CG7590_ESCL1_2249	-3.209	1.29.E-50	2.89.E-46	19463	A_09_P224725	-1.037	1.11.E-03	2.56.E-02
					34761	A_09_P063976	-0.961	1.79.E-03	3.22.E-02
scyl	CG7590_ESCL2_41	-1.679	2.16.E-28	4.35.E-24	19463	A_09_P224725	-1.037	1.11.E-03	2.56.E-02
					34761	A_09_P063976	-0.961	1.79.E-03	3.22.E-02
scyl	CG7590_ESCL3_2510	-2.719	4.76.E-32	9.93.E-28	19463	A_09_P224725	-1.037	1.11.E-03	2.56.E-02
					34761	A_09_P063976	-0.961	1.79.E-03	3.22.E-02
sda	CG5518_ESCL1_4707	-1.320	4.61.E-18	8.20.E-14	19914	A_09_P030891	-2.702	7.56.E-06	5.81.E-03
					22081	A_09_P030891	-2.612	5.57.E-06	5.81.E-03
Sema-5c	CG5661_ESCL2_3189	-2.750	8.15.E-35	1.73.E-30	24018	A_09_P065586	-1.412	3.90.E-04	1.53.E-02
					42804	A_09_P065586	-1.323	2.82.E-04	1.34.E-02
sif	CG5406_ESCL1_7798	-0.577	5.33.E-08	7.37.E-04	33444	A_09_P134315	-1.413	8.85.E-04	2.28.E-02
					28896	A_09_P004331	-1.390	3.09.E-03	4.09.E-02
					14467	A_09_P004336	-0.932	2.41.E-03	3.69.E-02
skl	CG13701_ESCL1_1187	-2.698	1.50.E-30	3.10.E-26	28876	A_09_P034256	-1.733	1.31.E-04	1.01.E-02
					18047	A_09_P034256	-1.618	1.01.E-04	9.35.E-03
skl	CG13701_ESCL2_239	-0.695	4.18.E-09	6.01.E-05	28876	A_09_P034256	-1.733	1.31.E-04	1.01.E-02
					18047	A_09_P034256	-1.618	1.01.E-04	9.35.E-03
slou	CG6534_ESCL1_2681	-3.545	4.70.E-30	9.64.E-26	37586	A_09_P043881	-0.924	3.14.E-03	4.11.E-02
slou	CG6534_ESCL2_1713	-0.652	3.16.E-09	4.56.E-05	37586	A_09_P043881	-0.924	3.14.E-03	4.11.E-02
sls	CG1915_ESCL1_15059	-3.418	1.75.E-32	3.67.E-28	38881	A_09_P045221	-1.201	4.37.E-03	4.82.E-02
sls	CG1915_ESCL3_14279	-1.352	1.31.E-19	2.38.E-15	38881	A_09_P045221	-1.201	4.37.E-03	4.82.E-02
sls	CG1915_ESCL4_1524	-1.648	4.83.E-13	7.77.E-09	38881	A_09_P045221	-1.201	4.37.E-03	4.82.E-02
sls	CG1915_ESCL5_2497	-1.081	1.52.E-11	2.35.E-07	38881	A_09_P045221	-1.201	4.37.E-03	4.82.E-02

sm	CG9218_ESCL1_2769	-1.043	1.44.E-18	2.58.E-14	21501	A_09_P192270	-1.924	4.12.E-03	4.69.E-02
					573	A_09_P187005	-1.567	3.48.E-03	4.31.E-02
					17355	A_09_P209295	-1.554	2.31.E-03	3.64.E-02
					41100	A_09_P049316	-1.271	5.54.E-04	1.82.E-02
					33261	A_09_P044786	-1.210	1.80.E-03	3.23.E-02
					18118	A_09_P044786	-1.153	1.99.E-03	3.40.E-02
sm	CG9218_ESCL2_1294	-0.502	1.35.E-07	1.84.E-03	21501	A_09_P192270	-1.924	4.12.E-03	4.69.E-02
					573	A_09_P187005	-1.567	3.48.E-03	4.31.E-02
					17355	A_09_P209295	-1.554	2.31.E-03	3.64.E-02
					41100	A_09_P049316	-1.271	5.54.E-04	1.82.E-02
					33261	A_09_P044786	-1.210	1.80.E-03	3.23.E-02
					18118	A_09_P044786	-1.153	1.99.E-03	3.40.E-02
sns	CG33141_ESCL1_7919	-1.259	1.41.E-20	2.61.E-16	15344	A_09_P076681	-0.941	1.75.E-03	3.20.E-02
					19232	A_09_P171072	-0.917	1.85.E-03	3.29.E-02
					42018	A_09_P076681	-0.890	3.28.E-03	4.20.E-02
Socs36E	CG15154_ESCL1_3320	-2.380	2.92.E-26	5.77.E-22	36849	A_09_P045976	-2.609	4.83.E-05	7.55.E-03
Socs36E	CG15154_ESCL2_1816	-0.928	4.20.E-12	6.60.E-08	36849	A_09_P045976	-2.609	4.83.E-05	7.55.E-03
Socs36E	CG15154_ESCL3_2088	-1.193	7.27.E-21	1.35.E-16	36849	A_09_P045976	-2.609	4.83.E-05	7.55.E-03
Sox14	CG3090_ESCL1_3088	-2.288	8.41.E-19	1.51.E-14	5155	A_09_P148280	-1.173	2.92.E-03	4.00.E-02
Sox14	CG3090_ESCL2_1937	-2.409	4.93.E-36	1.06.E-31	5155	A_09_P148280	-1.173	2.92.E-03	4.00.E-02
Sox15	CG8404_ESCL2_2291	-0.595	1.25.E-06	1.63.E-02	28541	A_09_P011551	-1.309	1.87.E-03	3.30.E-02
Spn100A	CG1342_ESCL1_1886	-3.335	1.73.E-42	3.82.E-38	57	A_09_P061456	-0.855	2.18.E-03	3.56.E-02
step	CG11628_ESCL1_1674	-0.957	1.71.E-29	3.50.E-25	585	A_09_P202550	-0.937	2.45.E-03	3.69.E-02
step	CG11628_ESCL2_1169	-0.726	1.24.E-12	1.98.E-08	585	A_09_P202550	-0.937	2.45.E-03	3.69.E-02
stv	CG32130_ESCL1_3133	-1.336	2.80.E-11	4.31.E-07	3913	A_09_P014671	-0.769	4.42.E-03	4.86.E-02
sty	CG1921_ESCL1_4183	-2.759	2.43.E-40	5.31.E-36	20530	A_09_P196120	-0.751	3.73.E-03	4.46.E-02
sty	CG1921_ESCL2_3138	-4.076	3.33.E-43	7.35.E-39	20530	A_09_P196120	-0.751	3.73.E-03	4.46.E-02
sty	CG1921_ESCL3_1673	-3.333	6.92.E-42	1.52.E-37	20530	A_09_P196120	-0.751	3.73.E-03	4.46.E-02
su(r)	CG2194_ESCL1_3025	-2.686	2.35.E-39	5.12.E-35	7675	A_09_P150565	-1.401	8.64.E-04	2.26.E-02
Sxl	CG33070_ESCL1_1763	-1.694	3.86.E-34	8.17.E-30	12810	A_09_P143460	-1.148	4.71.E-03	5.00.E-02
Sxl	CG33070_ESCL2_3064	-2.409	8.43.E-37	1.81.E-32	12810	A_09_P143460	-1.148	4.71.E-03	5.00.E-02
Sxl	CG33070_ESCL3_4822	-1.480	1.21.E-12	1.93.E-08	12810	A_09_P143460	-1.148	4.71.E-03	5.00.E-02
Sxl	CG33070_ESCL5_104	-0.436	1.01.E-10	1.53.E-06	12810	A_09_P143460	-1.148	4.71.E-03	5.00.E-02
Syt1	CG3139_ESCL1_3739	-0.850	5.46.E-07	7.24.E-03	26229	A_09_P049161	-1.365	1.73.E-04	1.13.E-02
tai	CG13109_ESCL1_7447	-0.671	6.47.E-08	8.92.E-04	12133	A_09_P063971	-1.132	1.65.E-03	3.11.E-02
					7514	A_09_P174985	-0.998	2.61.E-03	3.80.E-02
TepII	CG7052_ESCL1_4140	-1.856	1.16.E-34	2.47.E-30	5399	A_09_P213450	-2.198	3.48.E-04	1.46.E-02
					3608	A_09_P064071	-1.893	1.82.E-04	1.14.E-02
					6660	A_09_P064071	-1.815	2.74.E-04	1.33.E-02
Thor	CG8846_ESCL1_638	-0.710	3.07.E-12	4.85.E-08	14242	A_09_P032876	-1.678	7.32.E-05	8.48.E-03
					19101	A_09_P032876	-1.629	6.15.E-05	8.27.E-03
Tollo	CG6890_ESCL1_7040	-3.335	8.69.E-28	1.74.E-23	6312	A_09_P116390	-2.387	2.20.E-05	6.56.E-03
					34858	A_09_P191795	-2.227	1.90.E-05	6.34.E-03
					1575	A_09_P045751	-1.753	4.89.E-04	1.70.E-02
tw	CG12311_ESCL1_2541	-2.734	1.98.E-25	3.88.E-21	9814	A_09_P048691	-1.047	1.01.E-03	2.45.E-02
					898	A_09_P048691	-0.994	1.49.E-03	2.96.E-02
					19556	A_09_P048691	-0.981	1.41.E-03	2.86.E-02
					37239	A_09_P048691	-0.951	1.97.E-03	3.39.E-02
					35814	A_09_P048691	-0.929	1.48.E-03	2.95.E-02
					23014	A_09_P048691	-0.928	1.74.E-03	3.20.E-02
					13262	A_09_P048691	-0.921	1.99.E-03	3.40.E-02
					4101	A_09_P048691	-0.866	2.16.E-03	3.55.E-02
					17438	A_09_P048691	-0.861	3.09.E-03	4.09.E-02
					24847	A_09_P048691	-0.858	4.16.E-03	4.70.E-02
tw	CG12311_ESCL2_2229	-1.349	2.85.E-31	5.92.E-27	9814	A_09_P048691	-1.047	1.01.E-03	2.45.E-02
					898	A_09_P048691	-0.994	1.49.E-03	2.96.E-02
					19556	A_09_P048691	-0.981	1.41.E-03	2.86.E-02
					37239	A_09_P048691	-0.951	1.97.E-03	3.39.E-02
					35814	A_09_P048691	-0.929	1.48.E-03	2.95.E-02
					23014	A_09_P048691	-0.928	1.74.E-03	3.20.E-02
					13262	A_09_P048691	-0.921	1.99.E-03	3.40.E-02
					4101	A_09_P048691	-0.866	2.16.E-03	3.55.E-02
					17438	A_09_P048691	-0.861	3.09.E-03	4.09.E-02
					24847	A_09_P048691	-0.858	4.16.E-03	4.70.E-02
Ugt35a	CG6644_ESCL1_1535	-0.388	3.50.E-06	4.46.E-02	7652	A_09_P078396	-1.151	4.81.E-04	1.69.E-02
					44846	A_09_P078396	-1.016	1.91.E-03	3.34.E-02
Ugt86Da	CG18578_ESCL1_1748	-1.690	1.24.E-24	2.40.E-20	24564	A_09_P062526	-3.769	1.17.E-06	5.81.E-03
Ugt86Da	CG18578_ESCL2_1522	-1.466	1.36.E-19	2.47.E-15	24564	A_09_P062526	-3.769	1.17.E-06	5.81.E-03
unc-115	CG31332_ESCL1_2700	-3.216	1.43.E-38	3.12.E-34	21073	A_09_P073171	-0.960	2.17.E-03	3.55.E-02

unc-115	CG31332_ESCL2_2293	-0.582	3.06.E-09	4.42.E-05	21073	A_09_P073171	-0.960	2.17.E-03	3.55.E-02
unc-115	CG31352_ESCL1_3130	-2.469	6.55.E-29	1.33.E-24	21073	A_09_P073171	-0.960	2.17.E-03	3.55.E-02
unc-5	CG8166_ESCL1_4927	-3.662	1.16.E-43	2.58.E-39	44816	A_09_P179550	-1.300	1.98.E-04	1.18.E-02
unc-5	CG8166_ESCL2_3152	-2.400	2.32.E-22	4.38.E-18	44816	A_09_P179550	-1.300	1.98.E-04	1.18.E-02
vg	CG3830_ESCL1_3667	-1.857	3.82.E-12	6.00.E-08	33773	A_09_P009991	-1.668	2.24.E-03	3.60.E-02
					15442	A_09_P009991	-1.585	2.28.E-03	3.63.E-02
vg	CG3830_ESCL3_656	-0.891	5.44.E-10	8.06.E-06	33773	A_09_P009991	-1.668	2.24.E-03	3.60.E-02
					15442	A_09_P009991	-1.585	2.28.E-03	3.63.E-02
vir-1	CG31764_ESCL1_1584	-1.477	4.56.E-21	8.48.E-17	10243	A_09_P056621	-1.181	2.29.E-03	3.63.E-02
					33815	A_09_P056616	-1.096	2.32.E-03	3.64.E-02
					41688	A_09_P056616	-1.054	2.35.E-03	3.64.E-02
vir-1	CG31764_ESCL2_1202	-0.768	1.27.E-13	2.07.E-09	10243	A_09_P056621	-1.181	2.29.E-03	3.63.E-02
					33815	A_09_P056616	-1.096	2.32.E-03	3.64.E-02
					41688	A_09_P056616	-1.054	2.35.E-03	3.64.E-02
vir-1	CG31764_ESCL3_1023	-1.502	2.37.E-15	4.02.E-11	10243	A_09_P056621	-1.181	2.29.E-03	3.63.E-02
					33815	A_09_P056616	-1.096	2.32.E-03	3.64.E-02
					41688	A_09_P056616	-1.054	2.35.E-03	3.64.E-02
vri	CG14029_ESCL1_3258	-2.096	1.61.E-22	3.04.E-18	7188	A_09_P031491	-1.107	4.68.E-03	5.00.E-02
vri	CG14029_ESCL2_1725	-0.753	7.54.E-07	9.92.E-03	7188	A_09_P031491	-1.107	4.68.E-03	5.00.E-02
vri	CG14029_ESCL3_2928	-2.841	3.46.E-26	6.82.E-22	7188	A_09_P031491	-1.107	4.68.E-03	5.00.E-02
wb	CG15288_ESCL1_10722	-0.785	3.75.E-11	5.74.E-07	4966	A_09_P171275	-1.402	4.98.E-04	1.71.E-02
wb	CG15288_ESCL3_9821	-0.545	4.50.E-11	6.88.E-07	4966	A_09_P171275	-1.402	4.98.E-04	1.71.E-02
wnd	CG8789_ESCL1_4180	-1.440	5.49.E-25	1.07.E-20	33108	A_09_P034701	-1.114	8.55.E-04	2.25.E-02
wnd	CG8789_ESCL2_2836	-0.826	5.13.E-07	6.81.E-03	33108	A_09_P034701	-1.114	8.55.E-04	2.25.E-02
Wnt2	CG1916_ESCL1_2135	-2.372	1.96.E-30	4.03.E-26	6466	A_09_P010341	-1.165	1.29.E-03	2.75.E-02
Wnt2	CG1916_ESCL2_997	-1.722	8.94.E-29	1.81.E-24	6466	A_09_P010341	-1.165	1.29.E-03	2.75.E-02
Xbp1	CG9415_ESCL1_1936	-0.388	7.62.E-08	1.05.E-03	14073	A_09_P196055	-0.811	3.69.E-03	4.46.E-02
					13921	A_09_P032791	-0.781	4.23.E-03	4.73.E-02
yrt	CG9764_ESCL1_4239	-0.662	7.65.E-17	1.33.E-12	44012	A_09_P116530	-0.838	4.21.E-03	4.73.E-02
yrt	CG9764_ESCL2_2845	-1.420	4.91.E-24	9.47.E-20	44012	A_09_P116530	-0.838	4.21.E-03	4.73.E-02
yrt	CG9764_ESCL3_2576	-2.222	2.00.E-46	4.46.E-42	44012	A_09_P116530	-0.838	4.21.E-03	4.73.E-02
Z600	CG17962_ESCL1_192	-3.525	1.47.E-48	3.29.E-44	14931	A_09_P010096	-1.299	9.49.E-04	2.36.E-02
					42539	A_09_P010096	-1.267	1.93.E-03	3.36.E-02

Supplementary Table S6. List of PGC-enriched genes down-regulated in Ovo-B KD PGCs

* This table shows names of genes expressed predominantly in PGCs (Adjusted p-value < 0.05; see SI Materials and Methods) that were significantly down-regulated in Ovo-B KD PGCs in comparison with control PGCs (q-value < 0.05; see SI Materials and Methods).

† The cell-type effect (C_i) value was calculated using a linear model (see SI Materials and Methods). C_i represents the estimated fold change in PGCs relative to whole embryos (soma) in $\log_2$ scale.

‡ The genotype effect (G_i) value, or the estimated fold change in control PGCs vs Ovo-A–expressing PGCs in $\log_2$ scale (Table S1) is shown again.

	PGCs/soma				Cont./OvoB KD				
Gene Name*	Probe Name	Cell-type effect† [Fold Change; log2 (PGCs/soma)]	p-value	Adjusted p-value	Feature Number	Probe Name	Genotype effect ‡ [Fold Change; log2 (Cont./OvoB KD)]	p-value	q-value
adp	CG5124_ESCL1_1812	1.161	1.47E-28	2.97.E-24	33391	A_09_P041196	0.813	2.40.E-03	3.69.E-02
					20001	A_09_P041196	0.895	1.94.E-03	3.36.E-02
adp	CG5124_ESCL2_2098	1.265	3.08E-42	6.78.E-38	33391	A_09_P041196	0.813	2.40.E-03	3.69.E-02
					20001	A_09_P041196	0.895	1.94.E-03	3.36.E-02
alpha-Est10	CG1131_ESCL1_1558	1.677	1.02E-20	1.90.E-16	3628	A_09_P030956	1.877	3.64.E-05	7.50.E-03
					25877	A_09_P030956	1.936	2.32.E-04	1.25.E-02
					19336	A_09_P209670	2.395	6.88.E-05	8.27.E-03
alpha-Est3	CG1257_ESCL1_1535	2.498	2.34E-34	4.97.E-30	31459	A_09_P197385	1.369	1.98.E-04	1.18.E-02
					33181	A_09_P197645	1.523	8.11.E-05	8.73.E-03
					33886	A_09_P030966	1.852	6.27.E-05	8.27.E-03
					29917	A_09_P030966	1.927	4.21.E-05	7.50.E-03
alpha-Est4	CG1082_ESCL1_1561	0.807	3.22E-11	4.95.E-07	19915	A_09_P030971	0.841	3.34.E-03	4.24.E-02
aub	CG6137_ESCL1_2537	2.813	2.77E-59	6.25.E-55	18373	A_09_P041376	1.177	8.98.E-04	2.30.E-02
					24248	A_09_P041376	1.195	1.37.E-03	2.83.E-02
CG10747	CG10747_ESCL1_1417	0.404	3.31E-11	5.07.E-07	11506	A_09_P023676	0.900	3.45.E-03	4.30.E-02
					16108	A_09_P023676	0.991	1.98.E-03	3.39.E-02
CG10747	CG10747_ESCL2_876	0.650	1.82E-08	2.56.E-04	11506	A_09_P023676	0.900	3.45.E-03	4.30.E-02
					16108	A_09_P023676	0.991	1.98.E-03	3.39.E-02
CG11095	CG11095_ESCL1_1038	0.978	8.54E-16	1.46.E-11	11846	A_09_P038546	0.825	4.15.E-03	4.70.E-02
					28679	A_09_P038546	0.880	2.26.E-03	3.60.E-02
CG11382	CG11382_ESCL1_1336	1.551	9.90E-19	1.78.E-14	43541	A_09_P062866	2.757	1.21.E-05	5.98.E-03
CG11409	CG11409_ESCL1_4051	2.127	1.23E-32	2.58.E-28	27879	A_09_P076921	1.043	8.05.E-04	2.17.E-02
					2094	A_09_P076921	1.095	6.27.E-04	1.92.E-02
CG11409	CG11409_ESCL2_3772	1.534	6.47E-30	1.32.E-25	27879	A_09_P076921	1.043	8.05.E-04	2.17.E-02
					2094	A_09_P076921	1.095	6.27.E-04	1.92.E-02
CG11638	CG11638_ESCL1_2319	3.253	1.28E-41	2.81.E-37	9106	A_09_P062786	1.531	1.78.E-04	1.14.E-02
					20495	A_09_P062786	1.599	1.05.E-04	9.43.E-03
					33878	A_09_P199575	1.728	1.85.E-04	1.16.E-02
CG11638	CG11638_ESCL2_1032	2.942	1.19E-30	2.46.E-26	9106	A_09_P062786	1.531	1.78.E-04	1.14.E-02
					20495	A_09_P062786	1.599	1.05.E-04	9.43.E-03
					33878	A_09_P199575	1.728	1.85.E-04	1.16.E-02
CG12001	CG12001_ESCL1_1881	1.087	5.23E-17	9.14.E-13	5986	A_09_P036121	0.763	3.75.E-03	4.46.E-02
					5986	A_09_P036121	0.763	3.75.E-03	4.46.E-02
CG12325	CG12325_ESCL1_3040	0.618	9.09E-07	1.19.E-02	27057	A_09_P006231	1.394	2.74.E-04	1.33.E-02
CG12325	CG12325_ESCL2_2706	0.676	7.00E-20	1.28.E-15	27057	A_09_P006231	1.394	2.74.E-04	1.33.E-02
CG12477	CG12477_ESCL1_734	4.846	7.41E-38	1.61.E-33	24914	A_09_P113955	0.767	3.68.E-03	4.45.E-02
CG12499	CG12499_ESCL1_6094	0.358	1.98E-07	2.68.E-03	1544	A_09_P070216	0.754	4.51.E-03	4.91.E-02
					4622	A_09_P070216	0.768	4.08.E-03	4.66.E-02
CG12744	CG12744_ESCL1_830	1.004	2.00E-23	3.82.E-19	38336	A_09_P005866	1.657	8.59.E-05	8.82.E-03
					25426	A_09_P005866	1.679	6.19.E-05	8.27.E-03
					13616	A_09_P150055	1.733	5.63.E-05	7.91.E-03
CG12744	CG12744_ESCL2_372	1.050	4.74E-26	9.34.E-22	38336	A_09_P005866	1.657	8.59.E-05	8.82.E-03
					25426	A_09_P005866	1.679	6.19.E-05	8.27.E-03
					13616	A_09_P150055	1.733	5.63.E-05	7.91.E-03
CG12851	CG12851_ESCL1_3052	0.615	3.35E-06	4.27.E-02	12656	A_09_P027781	1.171	3.43.E-04	1.45.E-02
					4842	A_09_P027781	1.208	3.53.E-04	1.46.E-02
CG12972	CG12972_ESCL1_872	0.648	2.64E-12	4.16.E-08	33256	A_09_P035431	1.521	9.67.E-04	2.40.E-02
					66	A_09_P035431	1.596	9.89.E-04	2.42.E-02
CG12991	CG12991_ESCL1_2268	0.622	1.52E-10	2.28.E-06	29187	A_09_P213420	0.777	2.97.E-03	4.02.E-02
CG13350	CG13350_ESCL1_2208	1.018	7.04E-24	1.36.E-19	28127	A_09_P007501	0.858	2.09.E-03	3.49.E-02
					34758	A_09_P007501	0.863	2.70.E-03	3.84.E-02
CG13350	CG13350_ESCL2_2615	1.104	3.52E-35	7.52.E-31	28127	A_09_P007501	0.858	2.09.E-03	3.49.E-02

					34758	A_09_P007501	0.863	2.70.E-03	3.84.E-02
CG13741	CG13741_ESCL1_1881	2.278	1.08E-34	2.30.E-30	24688	A_09_P005567	1.182	8.01.E-04	2.17.E-02
CG13741	CG13741_ESCL2_965	2.148	4.23E-36	9.08.E-32	24688	A_09_P005567	1.182	8.01.E-04	2.17.E-02
CG1387	CG1387_ESCL1_3324	1.134	3.55E-20	6.52.E-16	4865	A_09_P068226	1.489	1.77.E-03	3.21.E-02
					12252	A_09_P068226	1.615	3.15.E-03	4.13.E-02
CG14512	CG14512_ESCL1_454	0.616	4.87E-12	7.63.E-08	4728	A_09_P060841	0.896	1.94.E-03	3.36.E-02
					6849	A_09_P060841	0.954	2.02.E-03	3.43.E-02
CG14516	CG14516_ESCL1_3206	1.059	9.65E-26	1.90.E-21	7384	A_09_P060846	2.718	7.15.E-06	5.81.E-03
CG14516	CG14516_ESCL2_2908	1.061	4.70E-29	9.55.E-25	7384	A_09_P060846	2.718	7.15.E-06	5.81.E-03
CG15172	CG15172_ESCL1_284	5.164	3.33E-35	7.10.E-31	7144	A_09_P023331	1.232	3.24.E-04	1.44.E-02
					9622	A_09_P162685	1.244	4.01.E-04	1.55.E-02
CG15390	CG15390_ESCL1_661	0.715	2.62E-17	4.60.E-13	12051	A_09_P018536	1.520	1.80.E-04	1.14.E-02
					21055	A_09_P018536	1.596	1.19.E-04	9.80.E-03
CG15771	CG15771_ESCL1_989	0.845	4.04E-19	7.30.E-15	44341	A_09_P067336	1.341	2.26.E-04	1.24.E-02
					7416	A_09_P067336	1.371	2.08.E-04	1.19.E-02
CG15771	CGX0749_ESCL1_717	1.162	3.59E-12	5.66.E-08	44341	A_09_P067336	1.341	2.26.E-04	1.24.E-02
					7416	A_09_P067336	1.371	2.08.E-04	1.19.E-02
CG1640	CG1640_ESCL1_2440	1.036	9.87E-17	1.71.E-12	32178	A_09_P038366	0.802	2.82.E-03	3.94.E-02
					4906	A_09_P038366	0.868	1.77.E-03	3.21.E-02
CG1640	CG1640_ESCL2_1642	0.800	2.34E-20	4.30.E-16	32178	A_09_P038366	0.802	2.82.E-03	3.94.E-02
					4906	A_09_P038366	0.868	1.77.E-03	3.21.E-02
CG30409	CG30409_ESCL1_536	2.888	3.63E-15	6.11.E-11	31581	A_09_P058351	0.761	4.04.E-03	4.64.E-02
CG31472	CG31472_ESCL2_639	0.529	1.59E-12	2.52.E-08	20911	A_09_P059946	2.235	3.31.E-05	7.50.E-03
CG31861	CG31861_ESCL1_415	0.540	8.03E-08	1.10.E-03	25457	A_09_P166490	1.010	1.22.E-03	2.68.E-02
CG32164	CG32164_ESCL1_3550	1.221	1.39E-22	2.64.E-18	25128	A_09_P191830	0.772	4.21.E-03	4.73.E-02
					29899	A_09_P162230	0.802	4.61.E-03	4.97.E-02
CG32164	CG32164_ESCL2_3168	1.202	4.65E-35	9.92.E-31	25128	A_09_P191830	0.772	4.21.E-03	4.73.E-02
					29899	A_09_P162230	0.802	4.61.E-03	4.97.E-02
CG32164	CG32165_ESCL1_3171	1.188	6.96E-22	1.31.E-17	25128	A_09_P191830	0.772	4.21.E-03	4.73.E-02
					29899	A_09_P162230	0.802	4.61.E-03	4.97.E-02
CG32318	CG9187_ESCL1_492	0.863	4.70E-15	7.90.E-11	306	A_09_P015271	1.475	1.94.E-04	1.17.E-02
CG32373	CG32373_ESCL1_1364	2.948	2.43E-37	5.25.E-33	44782	A_09_P015371	1.192	8.63.E-04	2.26.E-02
					15630	A_09_P015371	1.228	7.25.E-04	2.05.E-02
CG32719	CG32719_ESCL1_3596	0.510	1.25E-06	1.63.E-02	2626	A_09_P016331	2.965	2.85.E-03	3.96.E-02
CG3362	CG3362_ESCL1_1397	0.988	4.73E-26	9.31.E-22	41816	A_09_P027401	1.153	1.77.E-03	3.21.E-02
					5434	A_09_P027401	1.315	4.37.E-04	1.62.E-02
CG3362	CG3362_ESCL2_818	1.026	2.72E-30	5.59.E-26	41816	A_09_P027401	1.153	1.77.E-03	3.21.E-02
					5434	A_09_P027401	1.315	4.37.E-04	1.62.E-02
CG3371	CG3371_ESCL1_2231	1.991	9.94E-41	2.18.E-36	29906	A_09_P028056	1.308	2.25.E-04	1.24.E-02
					39863	A_09_P028056	1.348	2.44.E-04	1.26.E-02
CG3371	CG3371_ESCL2_1664	1.943	1.93E-44	4.28.E-40	29906	A_09_P028056	1.308	2.25.E-04	1.24.E-02
					39863	A_09_P028056	1.348	2.44.E-04	1.26.E-02
CG34353	CG12274_ESCL1_140	1.134	5.65E-15	9.47.E-11	9772	A_09_P218190	2.469	4.79.E-04	1.68.E-02
					38372	A_09_P003906	3.230	8.79.E-06	5.81.E-03
					13762	A_09_P003906	3.230	2.39.E-05	6.83.E-03
					8163	A_09_P205315	3.778	2.13.E-05	6.43.E-03
CG34353	CG12427_ESCL1_122	1.846	1.54E-25	3.02.E-21	9772	A_09_P218190	2.469	4.79.E-04	1.68.E-02
					38372	A_09_P003906	3.230	8.79.E-06	5.81.E-03
					13762	A_09_P003906	3.230	2.39.E-05	6.83.E-03
					8163	A_09_P205315	3.778	2.13.E-05	6.43.E-03
CG34353	CG12875_ESCL1_664	1.481	7.56E-19	1.36.E-14	9772	A_09_P218190	2.469	4.79.E-04	1.68.E-02
					38372	A_09_P003906	3.230	8.79.E-06	5.81.E-03
					13762	A_09_P003906	3.230	2.39.E-05	6.83.E-03
					8163	A_09_P205315	3.778	2.13.E-05	6.43.E-03
CG3608	CG3608_ESCL1_1465	0.513	2.11E-10	3.16.E-06	28936	A_09_P027621	1.055	1.40.E-03	2.86.E-02
					23658	A_09_P027621	1.095	1.01.E-03	2.45.E-02
CG3609	CG3609_ESCL1_930	0.485	3.60E-15	6.07.E-11	35091	A_09_P018531	0.841	2.63.E-03	3.81.E-02
					25888	A_09_P130625	1.385	2.96.E-04	1.37.E-02
CG40006	CG40006_ESCL1_2397	0.947	3.36E-09	4.85.E-05	7415	A_09_P001241	0.918	3.88.E-03	4.54.E-02
					18487	A_09_P001241	1.048	1.90.E-03	3.33.E-02
CG40006	CG40006_ESCL2_1996	1.013	2.57E-18	4.58.E-14	7415	A_09_P001241	0.918	3.88.E-03	4.54.E-02
					18487	A_09_P001241	1.048	1.90.E-03	3.33.E-02
CG42340	CG3367_ESCL1_1385	0.923	1.93E-13	3.13.E-09	2364	A_09_P067626	0.835	3.09.E-03	4.09.E-02
					34853	A_09_P067626	0.867	2.11.E-03	3.51.E-02
					39219	A_09_P067626	0.869	2.11.E-03	3.51.E-02
					7761	A_09_P067626	0.870	1.98.E-03	3.39.E-02
					37777	A_09_P067626	0.871	1.88.E-03	3.32.E-02
					11612	A_09_P067626	0.874	1.57.E-03	3.04.E-02
					25659	A_09_P179810	0.881	2.99.E-03	4.02.E-02

					34396	A_09_P067626	0.890	1.93.E-03	3.36.E-02
					1333	A_09_P067626	0.904	1.41.E-03	2.87.E-02
					10010	A_09_P067626	0.911	1.30.E-03	2.76.E-02
					19303	A_09_P067626	0.970	1.27.E-03	2.72.E-02
CG42404	CG4285_ESCL1_4492	0.865	9.13E-15	1.53.E-10	19986	A_09_P118075	1.097	1.79.E-03	3.22.E-02
					33659	A_09_P075361	1.713	5.71.E-05	7.93.E-03
					2884	A_09_P075361	1.733	6.86.E-05	8.27.E-03
CG42404	CG4285_ESCL2_2928	0.738	3.47E-11	5.31.E-07	19986	A_09_P118075	1.097	1.79.E-03	3.22.E-02
					33659	A_09_P075361	1.713	5.71.E-05	7.93.E-03
					2884	A_09_P075361	1.733	6.86.E-05	8.27.E-03
CG4250	CG4250_ESCL1_449	0.608	1.18E-06	1.53.E-02	17026	A_09_P026601	1.240	6.53.E-04	1.96.E-02
					26369	A_09_P026601	1.268	5.86.E-04	1.87.E-02
CG4306	CG4306_ESCL1_696	1.028	1.24E-08	1.75.E-04	34057	A_09_P034261	1.549	5.80.E-04	1.85.E-02
					10242	A_09_P034261	1.627	4.66.E-04	1.67.E-02
CG4306	CG4306_ESCL2_587	1.070	2.10E-08	2.94.E-04	34057	A_09_P034261	1.549	5.80.E-04	1.85.E-02
					10242	A_09_P034261	1.627	4.66.E-04	1.67.E-02
CG4771	CG4771_ESCL1_2096	1.825	5.22E-33	1.10.E-28	17900	A_09_P070396	1.074	2.22.E-03	3.60.E-02
CG4771	CG4771_ESCL2_1988	1.693	1.04E-44	2.30.E-40	17900	A_09_P070396	1.074	2.22.E-03	3.60.E-02
CG5018	CG5018_ESCL1_1880	0.343	2.23E-06	2.87.E-02	27695	A_09_P033426	0.811	2.50.E-03	3.74.E-02
					25431	A_09_P033426	0.814	2.23.E-03	3.60.E-02
CG5535	CG5535_ESCL1_1662	1.050	1.87E-23	3.59.E-19	32674	A_09_P034156	0.849	3.13.E-03	4.11.E-02
					27855	A_09_P034156	0.891	2.96.E-03	4.01.E-02
CG6084	CG6084_ESCL1_1240	1.577	3.53E-32	7.38.E-28	42697	A_09_P048596	1.008	1.49.E-03	2.96.E-02
					29180	A_09_P048596	1.053	8.39.E-04	2.22.E-02
CG6084	CG6084_ESCL2_931	1.846	5.60E-38	1.21.E-33	42697	A_09_P048596	1.008	1.49.E-03	2.96.E-02
					29180	A_09_P048596	1.053	8.39.E-04	2.22.E-02
CG6195	CG6195_ESCL1_885	1.609	6.82E-41	1.50.E-36	18986	A_09_P191375	1.062	1.73.E-03	3.20.E-02
					10030	A_09_P069266	1.365	3.78.E-04	1.51.E-02
CG6763	CG6763_ESCL1_974	1.812	8.76E-39	1.90.E-34	24089	A_09_P070606	0.929	1.99.E-03	3.40.E-02
CG6763	CG6763_ESCL2_1889	2.004	1.61E-25	3.16.E-21	24089	A_09_P070606	0.929	1.99.E-03	3.40.E-02
CG6770	CG6770_ESCL1_661	2.501	1.43E-48	3.21.E-44	26719	A_09_P022166	1.024	1.19.E-03	2.65.E-02
					19985	A_09_P196000	1.137	4.69.E-04	1.67.E-02
CG6770	CG6770_ESCL2_140	1.742	4.25E-39	9.27.E-35	26719	A_09_P022166	1.024	1.19.E-03	2.65.E-02
					19985	A_09_P196000	1.137	4.69.E-04	1.67.E-02
CG9004	CG9004_ESCL1_2433	0.286	1.12E-06	1.46.E-02	43659	A_09_P028731	1.185	2.37.E-03	3.66.E-02
					14338	A_09_P028731	1.225	1.41.E-03	2.86.E-02
CG9101	CG9101_ESCL1_413	0.522	1.17E-07	1.60.E-03	3504	A_09_P130125	1.018	9.40.E-04	2.36.E-02
CycJ	CG10308_ESCL1_1340	3.664	2.52E-52	5.66.E-48	12139	A_09_P012226	0.854	3.36.E-03	4.25.E-02
					38547	A_09_P012226	0.910	2.63.E-03	3.81.E-02
CycJ	CG10308_ESCL2_1022	2.134	3.10E-28	6.24.E-24	12139	A_09_P012226	0.854	3.36.E-03	4.25.E-02
					38547	A_09_P012226	0.910	2.63.E-03	3.81.E-02
Cyp6a20	CG10245_ESCL1_1472	0.867	1.49E-07	2.03.E-03	41546	A_09_P007841	1.384	2.73.E-04	1.33.E-02
					3199	A_09_P007841	1.437	1.90.E-04	1.17.E-02
					25399	A_09_P132155	1.783	1.03.E-04	9.38.E-03
Cyp6a20	CG10245_ESCL2_1196	0.685	4.85E-12	7.61.E-08	41546	A_09_P007841	1.384	2.73.E-04	1.33.E-02
					3199	A_09_P007841	1.437	1.90.E-04	1.17.E-02
					25399	A_09_P132155	1.783	1.03.E-04	9.38.E-03
dl	CG6667_ESCL3_1972	0.412	9.93E-07	1.30.E-02	9978	A_09_P181510	1.007	8.68.E-04	2.26.E-02
					2378	A_09_P119495	1.441	9.43.E-04	2.36.E-02
					2019	A_09_P041991	1.544	1.20.E-03	2.66.E-02
					45180	A_09_P041991	1.652	8.48.E-04	2.23.E-02
DNApol-alpha73	CG5923_ESCL1_1770	0.635	2.86E-13	4.64.E-09	9531	A_09_P011801	0.768	4.40.E-03	4.85.E-02
fal	CG9670_ESCL1_1561	0.734	6.50E-18	1.15.E-13	14063	A_09_P064906	0.783	4.66.E-03	4.99.E-02
fal	CG9670_ESCL2_1197	0.897	6.07E-32	1.27.E-27	14063	A_09_P064906	0.783	4.66.E-03	4.99.E-02
fs(1)M3	CG4790_ESCL1_5206	1.543	4.97E-26	9.80.E-22	3686	A_09_P011436	4.079	3.78.E-06	5.81.E-03
					26649	A_09_P011436	4.160	4.04.E-06	5.81.E-03
Fsh	CG7665_ESCL1_3068	0.908	1.79E-13	2.92.E-09	28808	A_09_P031551	1.909	3.06.E-05	7.10.E-03
					24107	A_09_P031551	2.001	4.21.E-05	7.50.E-03
gammaTub37C	CG17566_ESCL1_1293	1.416	4.29E-24	8.27.E-20	39685	A_09_P011941	1.848	1.14.E-04	9.57.E-03
Gycalpha99B	CG1912_ESCL2_1952	1.953	2.85E-21	5.31.E-17	23342	A_09_P029986	1.105	4.30.E-03	4.78.E-02
					20483	A_09_P029986	1.122	4.61.E-04	1.66.E-02
hoip	CG3949_ESCL1_318	1.450	1.34E-24	2.59.E-20	30094	A_09_P030816	0.783	4.54.E-03	4.93.E-02
kkv	CG2666_ESCL1_5223	2.460	2.13E-34	4.53.E-30	38044	A_09_P042961	1.727	6.54.E-05	8.27.E-03
					6851	A_09_P042961	1.901	7.46.E-05	8.48.E-03
kkv	CG2666_ESCL2_4662	1.901	1.48E-28	2.99.E-24	38044	A_09_P042961	1.727	6.54.E-05	8.27.E-03
					6851	A_09_P042961	1.901	7.46.E-05	8.48.E-03
l(1)G0196	CG14616_ESCL2_5188	0.718	2.10E-20	3.87.E-16	38469	A_09_P079166	0.921	1.20.E-03	2.66.E-02
					34522	A_09_P180275	0.956	1.66.E-03	3.13.E-02
					29336	A_09_P079166	0.967	9.67.E-04	2.40.E-02

					39621	A_09_P225480	0.990	1.55.E-03	3.03.E-02
l(2)35Df	CG4152_ESCL1_3524	0.656	6.67E-07	8.80.E-03	16061	A_09_P043151	0.813	2.61.E-03	3.80.E-02
					24851	A_09_P043151	0.916	3.60.E-03	4.39.E-02
l(2)35Df	CG4152_ESCL2_3095	0.550	1.31E-17	2.30.E-13	16061	A_09_P043151	0.813	2.61.E-03	3.80.E-02
					24851	A_09_P043151	0.916	3.60.E-03	4.39.E-02
l(2)tid	CG5504_ESCL1_1944	1.301	2.20E-38	4.78.E-34	21765	A_09_P043231	0.754	4.07.E-03	4.66.E-02
l(2)tid	CG5504_ESCL2_1416	1.317	6.68E-32	1.39.E-27	21765	A_09_P043231	0.754	4.07.E-03	4.66.E-02
La	CG10922_ESCL1_1252	1.601	5.75E-41	1.26.E-36	29968	A_09_P013281	0.724	4.30.E-03	4.78.E-02
La	CG10922_ESCL2_1483	1.849	3.69E-41	8.10.E-37	29968	A_09_P013281	0.724	4.30.E-03	4.78.E-02
La	CG10922_ESCL3_1063	1.867	1.61E-44	3.58.E-40	29968	A_09_P013281	0.724	4.30.E-03	4.78.E-02
Lasp	CG3849_ESCL2_1403	0.692	1.69E-08	2.38.E-04	35348	A_09_P001951	0.874	2.26.E-03	3.60.E-02
					4661	A_09_P137855	0.907	2.95.E-03	4.01.E-02
					15322	A_09_P001951	0.952	1.44.E-03	2.91.E-02
lectin-33A	CG16834_ESCL1_264	0.938	1.22E-08	1.73.E-04	43505	A_09_P062351	1.142	1.20.E-03	2.66.E-02
					7649	A_09_P062351	1.234	7.43.E-04	2.09.E-02
metl	CG13929_ESCL1_823	0.386	9.34E-09	1.33.E-04	1968	A_09_P196905	0.959	1.40.E-03	2.85.E-02
mtg	CG7549_ESCL1_1958	2.259	3.56E-37	7.67.E-33	22590	A_09_P072466	1.240	3.87.E-04	1.53.E-02
					7546	A_09_P072466	1.322	2.06.E-04	1.19.E-02
mtg	CG7549_ESCL2_1341	1.253	4.12E-20	7.55.E-16	22590	A_09_P072466	1.240	3.87.E-04	1.53.E-02
					7546	A_09_P072466	1.322	2.06.E-04	1.19.E-02
NaCP60E	CG18506_ESCL2_873	0.995	4.92E-17	8.60.E-13	36318	A_09_P204800	0.782	3.09.E-03	4.09.E-02
NaCP60E	CG9071_ESCL2_1590	0.905	4.61E-12	7.23.E-08	36318	A_09_P204800	0.782	3.09.E-03	4.09.E-02
Nhe3	CG11328_ESCL1_3622	1.229	1.39E-28	2.82.E-24	43568	A_09_P187125	0.781	3.77.E-03	4.47.E-02
					28789	A_09_P065686	0.955	2.32.E-03	3.64.E-02
					34865	A_09_P065686	0.956	2.26.E-03	3.60.E-02
					42615	A_09_P065681	1.084	2.27.E-03	3.60.E-02
					29367	A_09_P065681	1.166	8.39.E-04	2.22.E-02
nht	CG15259_ESCL1_709	1.216	2.55E-15	4.31.E-11	6091	A_09_P111250	1.386	3.35.E-03	4.25.E-02
					28189	A_09_P064001	1.395	1.01.E-03	2.45.E-02
					22204	A_09_P064001	1.447	1.51.E-04	1.05.E-02
nrv3	CG8663_ESCL1_1464	0.822	1.02E-06	1.33.E-02	14276	A_09_P194020	1.142	4.34.E-04	1.61.E-02
					40071	A_09_P194995	1.574	7.46.E-05	8.48.E-03
					11261	A_09_P183115	1.644	1.09.E-04	9.43.E-03
nrv3	CG8663_ESCL2_873	0.991	6.60E-07	8.71.E-03	14276	A_09_P194020	1.142	4.34.E-04	1.61.E-02
					40071	A_09_P194995	1.574	7.46.E-05	8.48.E-03
					11261	A_09_P183115	1.644	1.09.E-04	9.43.E-03
nwk	CG4641_ESCL2_773	0.466	6.81E-08	9.38.E-04	27310	A_09_P054251	2.487	3.54.E-04	1.46.E-02
					23671	A_09_P054251	2.596	4.11.E-04	1.57.E-02
orb	CG10868_ESCL2_2673	0.575	6.20E-11	9.43.E-07	37166	A_09_P011246	0.984	1.09.E-03	2.54.E-02
					20239	A_09_P050141	1.252	2.60.E-04	1.29.E-02
					34906	A_09_P182220	1.611	3.29.E-04	1.45.E-02
					16291	A_09_P011251	1.774	1.57.E-04	1.07.E-02
ovo	CG6824_ESCL1_5416	1.688	2.57E-32	5.37E-28	24176	A_09_P134245	2.995	1.38E-05	6.14.E-03
	CG6824_ESCL2_3972	1.354	1.25E-11	1.94E-07	24176	A_09_P134245	2.995	1.38E-05	6.14.E-03
pex13	CG4663_ESCL2_1191	0.444	5.06E-10	7.50.E-06	41711	A_09_P007186	0.730	4.65.E-03	4.99.E-02
piwi	CG6122_ESCL1_2934	2.811	4.18E-47	9.33.E-43	12346	A_09_P011196	0.727	4.25.E-03	4.75.E-02
piwi	CG6122_ESCL2_2466	3.045	5.30E-60	1.20.E-55	12346	A_09_P011196	0.727	4.25.E-03	4.75.E-02
qin	CG14303_ESCL1_5119	2.644	1.95E-38	4.23.E-34	42962	A_09_P068931	0.821	2.95.E-03	4.01.E-02
					13327	A_09_P068931	0.828	2.29.E-03	3.63.E-02
					27447	A_09_P206515	1.060	9.47.E-04	2.36.E-02
					42009	A_09_P163755	1.145	6.23.E-04	1.92.E-02
qin	CG14303_ESCL2_4520	2.428	2.25E-24	4.36.E-20	42962	A_09_P068931	0.821	2.95.E-03	4.01.E-02
					13327	A_09_P068931	0.828	2.29.E-03	3.63.E-02
					27447	A_09_P206515	1.060	9.47.E-04	2.36.E-02
					42009	A_09_P163755	1.145	6.23.E-04	1.92.E-02
r-l	CG3593_ESCL1_1919	0.370	3.17E-06	4.05.E-02	21963	A_09_P044421	0.811	2.35.E-03	3.64.E-02
r-l	CG3593_ESCL2_1200	0.524	2.54E-14	4.20.E-10	21963	A_09_P044421	0.811	2.35.E-03	3.64.E-02
RecQ4	CG7487_ESCL1_4676	0.688	3.87E-12	6.08.E-08	3236	A_09_P049651	0.802	2.42.E-03	3.69.E-02
RecQ4	CG7487_ESCL2_1393	1.007	1.86E-11	2.88.E-07	3236	A_09_P049651	0.802	2.42.E-03	3.69.E-02
rhi	CG10683_ESCL2_1169	0.457	1.79E-06	2.32.E-02	42902	A_09_P010496	0.869	2.84.E-03	3.95.E-02
					33161	A_09_P010496	0.925	1.49.E-03	2.96.E-02
RpL22-like	CG9871_ESCL1_1090	5.821	6.81E-29	1.38.E-24	32995	A_09_P026846	0.764	3.76.E-03	4.47.E-02
					2427	A_09_P026846	0.823	2.35.E-03	3.64.E-02
RpL22-like	CG9871_ESCL2_317	4.243	2.66E-35	5.68.E-31	32995	A_09_P026846	0.764	3.76.E-03	4.47.E-02
					2427	A_09_P026846	0.823	2.35.E-03	3.64.E-02
RpL22-like	CG9871_ESCL3_1086	4.829	6.25E-28	1.26.E-23	32995	A_09_P026846	0.764	3.76.E-03	4.47.E-02
					2427	A_09_P026846	0.823	2.35.E-03	3.64.E-02
Rpt4R	CG7257_ESCL1_1098	4.614	4.32E-36	9.27.E-32	31156	A_09_P055181	1.722	4.91.E-05	7.55.E-03
					536	A_09_P055181	1.814	6.76.E-05	8.27.E-03

Sip1	CG10939_ESCL1_1577	0.806	4.06E-21	7.55.E-17	2122	A_09_P012691	2.171	1.68.E-05	6.14.E-03
Sip1	CG10939_ESCL2_807	1.064	1.89E-25	3.70.E-21	2122	A_09_P012691	2.171	1.68.E-05	6.14.E-03
SK	CG10706_ESCL2_4724	1.497	2.62E-22	4.94.E-18	5891	A_09_P067206	1.118	1.65.E-03	3.11.E-02
					10575	A_09_P140795	1.198	4.73.E-04	1.67.E-02
					20577	A_09_P067206	1.660	2.43.E-04	1.26.E-02
Sod	CG11793_ESCL1_671	0.366	2.70E-09	3.91.E-05	33844	A_09_P206585	1.224	1.74.E-03	3.20.E-02
Sod	CG11793_ESCL2_346	0.596	1.03E-08	1.46.E-04	33844	A_09_P206585	1.224	1.74.E-03	3.20.E-02
Sodh-2	CG4649_ESCL1_1210	0.452	9.17E-07	1.20.E-02	22800	A_09_P032981	1.154	6.39.E-04	1.94.E-02
Sodh-2	CG4649_ESCL2_1019	1.378	1.39E-45	3.10.E-41	22800	A_09_P032981	1.154	6.39.E-04	1.94.E-02
Spargel	CG9809_ESCL1_4498	1.184	2.84E-22	5.36.E-18	42961	A_09_P036066	0.788	2.64.E-03	3.82.E-02
					15141	A_09_P036066	0.926	1.30.E-03	2.76.E-02
Spargel	CG9809_ESCL3_2983	0.449	1.83E-07	2.47.E-03	42961	A_09_P036066	0.788	2.64.E-03	3.82.E-02
					15141	A_09_P036066	0.926	1.30.E-03	2.76.E-02
tej	CG8589_ESCL1_1847	1.597	1.28E-45	2.84.E-41	43307	A_09_P007621	1.178	3.66.E-04	1.48.E-02
					20904	A_09_P007621	1.200	3.24.E-04	1.44.E-02
tej	CG8589_ESCL2_1599	1.637	1.75E-17	3.07.E-13	43307	A_09_P007621	1.178	3.66.E-04	1.48.E-02
					20904	A_09_P007621	1.200	3.24.E-04	1.44.E-02
TM4SF	CG11303_ESCL1_1417	1.885	1.93E-16	3.33.E-12	13973	A_09_P032271	0.747	4.15.E-03	4.70.E-02
					40692	A_09_P193295	0.748	3.75.E-03	4.46.E-02
Vsx2	CG15783_ESCL1_855	1.049	1.79E-25	3.51.E-21	17887	A_09_P000916	1.027	4.65.E-03	4.99.E-02
wcd	CG7989_ESCL1_1443	0.426	9.67E-11	1.47.E-06	17401	A_09_P032871	0.857	3.46.E-03	4.30.E-02
					3601	A_09_P032871	0.898	2.95.E-03	4.01.E-02
X11Lbeta	CG32677_ESCL1_4803	1.100	3.88E-11	5.94.E-07	20683	A_09_P016181	0.741	3.75.E-03	4.46.E-02
					5628	A_09_P110490	1.196	1.07.E-03	2.52.E-02
					31465	A_09_P183230	1.412	2.33.E-04	1.25.E-02
					27079	A_09_P162075	2.284	8.36.E-05	8.80.E-03
					20516	A_09_P175655	2.529	1.48.E-04	1.05.E-02
yip3	CG13549_ESCL1_476	3.061	1.36E-32	2.85.E-28	15888	A_09_P062256	0.891	4.42.E-03	4.86.E-02

Supplementary Table S7. Primer sequences for cloning *ovo* isoforms and for constructing gRNA expression vectors and EGFP knock-in constructs

Primer name	primer sequence (5'-3')*
ovoA5'-Fw	ATGGTACCATGAACGTCAACAAAAATGATCTTC
ovoA5'-Rv	GCTTTGGATGAGTGGCTTTGTG
ovoA3'-Fw	CATCATTCGCACCATCATGG
ovoA3'-Rv	ATAAGCTTCTAATTGTGGACTGGCATGGG
ovo-probe-Fw	ATGGTACCTGATTACGCTGATATCCACGC
ovo-probe-Rv	ATGAGCTCATTGTGGACTGGCATGGG
svb-probe-Fw	ATGGATCCCGCTTAACTCACCTGGGC
svb-probe-Rv	ATAAGCTTCATTCGACTTCCAATTCCG
ovoA-sp-Fw	ATGGATCCTTGCCCGTTTTGTCTTTGC
ovoA-sp-Rv	ATTCTAGAGCCGGTATTATAGAATCGTGGG
ovoB-sp-Fw	ATGGATCCACAGTTACATAGCAATCGTCCG
ovoB-sp-Rv	ATTCTAGAGCCGGTATTATAGAATCGTGGG
ovoA-egfp-sgRNA-Fw	CTTCTCGTAAGAATATCCGAGAGA
ovoA-egfp-sgRNA-Rv	AAACTCTCTCGGATATTCTTACGA
ovoA-up1kb-Fw	GGGAACAAAAGCTGG*TGTGTGCTGGTGTAATTACAAACATTTTAC*
ovoA-up1kb-Rv	GCCCTTGCTCACCAT*TTGCTGAGCGAACGGATTTG*
ovoA-dwn1kb-Fw	GACGAGCTGTACAAGATGAACGTCAACAAAAATGATCTTCG**C**AAGAA**C**ATCCG**C**<br>GAG**C**G**C***GCGCTTTCAGCTTTGGTTTGAAAAC*
ovoA-dwn1kb-Rv	TATAGGGCGAATTGG*TGGGGATACTCCTTGCGTTGG*

ovoB-egfp-sgRNA-Fw	CTTCGGTATGGGCGGTGGTCGCGA
ovoB-egfp-sgRNA-Rv	AAACTCGCGACCACCGCCCATACC
ovoB-up1kb-Fw	GGGAACAAAAGCTGG*CAGAGCCGCCACCTCCG*
ovoB-up1kb-Rv	GCCCTTGCTCACCAT*ACCACCACCGCCACCG*
ovoB-dwn1kb-Fw	GACGAGCTGTACAAGATGGGCGG**C**GG**C**CGCGA**T***GGCCGAGGGAACTACGGAC*
ovoB-dwn1kb-Rv	TATAGGGCGAATTGG*GCTGCTGCTGCTGATTGCTG*
pEGFP-N1-Fw	ATGGTGAGCAAGGGCGAGG
pEGFP-N1-Rv	CTTGTACAGCTCGTCCATGCCG
pBlue-Fw	CCAGCTTTTGTTCCCTTTAGTGAGG
pBlue-Rv	CCAATTCGCCCTATAGTGAGTCGT

* Recognition sequences of restriction enzyme (*Kpn*I, *Hind*III, *Sac*I, *Bam*HI, and *Xba*I) are underlined. The overlapping ends for Gibson Assembly are double underlined. To avoid re-cutting of the knock-in allele by gRNA and Cas9, synonymous base substitutions were introduced (bold). The sequences annealing to genomic DNA are indicated in italics.

Supplementary Table S8. Primer sequences for qRT-PCR

Primer name	Primer sequence (5'-3')
ovo-A-Fw	TTGCCCGTTTTGTCTTTGC
ovo-A-Rv	TGCTGAGCGAACGGATTTG
ovo-B-Fw *	GACAAATTTCTGAGAATCGCACTTC
ovo-B-Rv *	TATTTTGTGCAGTGGTGGCAG
rp49-Fw	AGCGCACCAAGCACTTCATC
rp49-Rv	GACGCACTCTGTTGTCGATACC
vas-Fw	TGGAAGACCCCAGGTAGTGATTGTA
vas-Rv	AACGAGGTGCCTCCGTAAACAATA
nos-Fw	ACACGATTAAGTACTGCCCCAAGAA
nos-Rv	CGCCCTCTCTAAACCTTCATCTGTT
CG14967-Fw	TCTGAACAAGATTCGTCCTTGCCTA
CG14967-Rv	GCATTGTTGATGACCAGACTTAGGG

* The ovo-B-Fw/ovo-B-Rv primer pair detects two types of *ovo-B* transcripts, ovo-RA (FBtr0070740) and ovo-RD (FBtr0100408), according to FlyBase (http://flybase.org). ovo-RA and ovo-RD encode almost identical proteins, and no functional differences have been reported. Only 53 amino acids is inserted in the protein encoded by ovo-RA.

Supplementary References

1. Gratz, S. J. *et al*. Highly specific and efficient CRISPR/Cas9-catalyzed homology-directed repair in *Drosophila*. *Genetics* **196,** 961–971 (2014).

2. Kondo, S. & Ueda, R. Highly Improved gene targeting by germline-specific Cas9 expression in *Drosophila*. *Genetics* **195,** 715–721 (2013).

3. Kobayashi, S., Amikura, R., Nakamura, A. & Lasko, P. F. Techniques for analysing protein and RNA distribution in *Drosophila* ovaries and embryos at structural and ultrastructural resolution in *A comparative methods approach to the study of oocytes and embryos* (ed. Richter, J. D.) 426–445 (Oxford University Press, 1999).

4. Hayashi, Y., Hayashi, M. & Kobayashi, S. Nanos suppresses somatic cell fate in *Drosophila* germ line. *Proc. Natl. Acad. Sci. U. S. A.* **101,** 10338–10342 (2004).

5. Brown, N. H. & Kafatos, F. C. Functional cDNA libraries from *Drosophila* embryos. *J. Mol. Biol.* **203,** 425–437 (1988).

6. Shigenobu, S., Arita, K., Kitadate, Y., Noda, C. & Kobayashi, S. Isolation of germline cells from *Drosophila* embryos by flow cytometry. *Dev. Growth Differ.* **48,** 49–57 (2006).

7. Hashiyama, K., Hayashi, Y. & Kobayashi, S. *Drosophila Sex lethal* gene initiates female development in germline progenitors. *Science* **333,** 885–888 (2011).

8. Storey, J. D. & Tibshirani, R. Statistical significance for genomewide studies. *Proc. Natl. Acad. Sci. U. S. A.* **100,** 9440–9445 (2003).
